# Enhancing Polyhydroxyurethane Properties via the Formation of Dioxaborolane- and Dioxazaborocane Vitrimers

**DOI:** 10.1021/acs.chemmater.5c01048

**Published:** 2025-09-11

**Authors:** Sergei V. Zubkevich, Arpan Datta Sarma, Oleg N. Antzutkin, Reiner Dieden, Vincent Berthe, Stephan Westermann, Alexander S. Shaplov, Daniel F. Schmidt

**Affiliations:** † 87145 Luxembourg Institute of Science and Technology (LIST), Functional Polymeric and Particulate Materials Unit, 5 Avenue des Hauts-Fourneaux, Esch-sur-Alzette L-4362, Luxembourg; ‡ Chemistry of Interfaces, Luleå University of Technology, Luleå SE-97187, Sweden

## Abstract

While polyhydroxyurethanes (PHUs) have been offered as “greener” alternatives to conventional polyurethanes (PUs) for ∼15 years, their low molecular weights, high hydrophilicity, and water uptake limit practical utility. In this study, we directly address these limitations by coupling pendant hydroxyl groups in tailored thermoplastic PHUs with 1,4-phenylenediboronic acid to form tough, robust PHU vitrimers, whose enhanced viscoelastic properties and reduced moisture sensitivity significantly differentiate them from existing linear and vitrimeric PHUs. Three families of PHU vitrimers incorporate either dioxaborolane or dioxazaborocane moieties based on aromatic or aliphatic amines. A fundamental comparison of structure–property relationships confirmed that dioxazaborocanes stabilized by aliphatic nitrogen atoms improve thermal and hydrolytic stability, as demonstrated through direct observations via solid-state NMR. In contrast to aromatic nitrogens, aliphatic nitrogens form N→B dative bonds, reducing chain mobility and increasing *T*_g_. This significantly enhances mechanical performance (+65–170% tensile strength, +125–300% break strain, ∼4–11× increase in tensile toughness) vs the parent PHUs and allows the resulting vitrimers to maintain their viscoelastic properties even at elevated humidity levels. Moreover, these N-stabilized dioxazaborocane PHU vitrimers are readily recyclable, both mechanically (up to at least 3×) and chemically (using ethanol/NaOH_aq_), without loss of performance.

## Graphical abstract

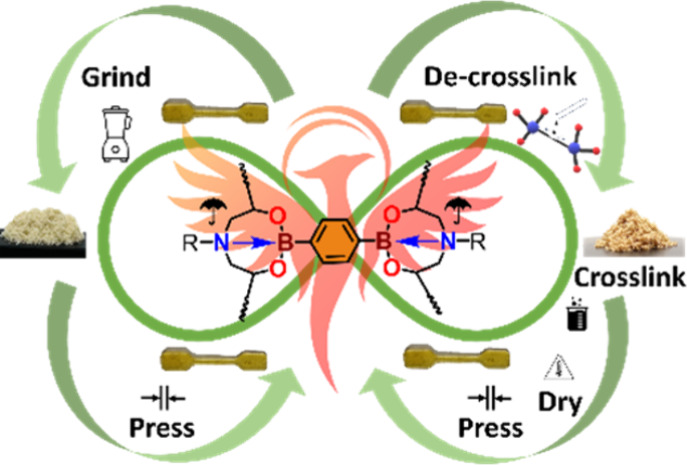

## Introduction

Polyurethanes (PUs) are a diverse family of plastics with a vast range of applications, including foams, coatings, adhesives, automotive parts, medical devices, sporting goods, etc.^1^ Such versatility is enabled by the broad selection of available monomers (isocyanates and polyols) for their synthesis. However, isocyanates are toxic compounds that present respiratory and dermal hazards and can cause chronic illness or even death upon overexposure.^2,3^ Moreover, monomeric diisocyanates used in the production of linear thermoplastic polyurethanes (TPUs), such as 4,4’-methylenediphenyl diisocyanate (MDI) and toluene diisocyanate (TDI), have been placed under REACH restriction since 2020.^4^

Nonisocyanate-based polyurethanes (NIPUs) have emerged as promising, environmentally friendly and potential “greener” alternatives to conventional PUs and have been studied for over 15 years.^5–10^ Poly­(hydroxyurethane)­s (PHUs), which are synthesized through the aminolysis of cyclic carbonates (CCs)^11,12^ represent the most well-known subclass of NIPUs due to the 100% atom economy of the reaction and easily synthesized, minimally toxic CCs. However, despite substantial progress in optimizing PHU synthesis, its commercial viability remains limited. This is primarily due to challenges such as *low molecular weights* due to slow polymerization kinetics and the presence of side reactions,^13,14^ *high hydrophilicity* and *excessive water uptake* due to high concentrations of hydroxyl groups,^7,15,16^ that compromise their mechanical performance.

One potential solution to enhance the properties^17,18^ of PHU-based materials while preserving their recyclability is to convert them into PHU vitrimers. The chemical structures, recycling conditions, and mechanical properties of PHU-based vitrimers reported to date are summarized in Table S5 (see the Supporting Information, SI). Broadly, PHU-based vitrimers can be classified into five subcategories based on the type of dynamic bonds incorporated into their chemical structure: (1) intrinsic PHU networks, which are inherently dynamic due to the presence of carbamate bonds and pendant hydroxyl groups in their structure^19–32^ (Figure 1A, Table S5, entries 1–14), which enable both associative and dissociative transcarbamoylation exchange; (2) PHU networks containing disulfide dynamic bonds (Figure 1B, Table S5, entries 15–19);^33–37^ (3) PHU networks with imine dynamic bonds (Figure 1C, Table S5, entries 19–20);^34,38^ (4) PHU networks incorporating dioxaborolane (Figure 1D, Table S5, entry 21);^39^ and (5) dioxazaborocane dynamic bonds (Figure 1E, Table S5, entry 22).

**Figure 1:**
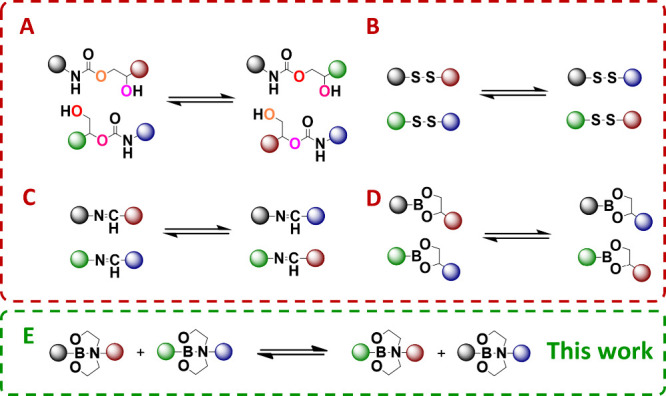
Types of dynamic exchanges reported in PHU-based vitrimers: (A) transcarbamoylation; (B) disulfide exchange; (C) imine exchange; (D) dioxaborolane exchange; and (E) exchange of dioxazaborocanes as suggested in this work.

Most reported PHU-based vitrimers that rely on transcarbamoylation reactions operate without the use of added catalysts. As a result, they typically require relatively high recycling temperatures, ranging from 110 to 200 °C (Table S5, entries 1–14 and Figure 2A). The incorporation of additional tertiary amine groups as internal catalysts has been shown to reduce the recycling temperature to 130 °C^24^ and to shorten the recycling time to 30 min (Table S5, entries 1 and 4). The glass transition temperatures (*T*_g_) of these PHU vitrimers generally fall within the range of 25–55 °C (Table S5 and Figure 2A), but can drop below room temperature when bio-oil-based (e.g., linseed oil^31^ or soybean oil^32^) or oligomeric monomers (such as Jeffamine,^24^ amino-terminated liquid nitrile rubber,^26^ etc.) are used. Notably, only the application of dual dynamic chemistries, specifically the combination of diamines with both biscarbonates and bisepoxides, along with aromatic monomers such as *m*-xylylene diamine, enabled the synthesis of vitrimers with *T*_g_ values as high as 94 °C^29^ (Table S5, entry 6). The recycling conditions of PHU-based vitrimers, specifically recycling temperature and duration, depend on the material’s *T*_g_ and rigidity (Table S5 and Figure 2A). Soft, elastomeric vitrimers typically require shorter recycling times (40–120 min) and lower temperatures (60–140 °C). In contrast, materials with *T*_g_ values above 25 °C are generally recycled at higher temperatures (140–200 °C) over longer durations (120–580 min).^22,23,25,27^ The mechanical properties of low-*T*_g_ (<25 °C) PHU networks are comparable to those of elastomers, characterized by high elongation at break (100–1300%) and average tensile strength (0.5–47 MPa), depending on the network architecture and cross-link density (Table S5 and Figure 2B).^19–24,40^ Only vitrimers with *T*_g_ values above 35 °C demonstrate the high tensile strength (69–103 MPa)^25,27,29–31^ necessary for applications as engineering plastics (Figure 2B). Thus, there is a clear trade-off between the *T*_g_ and mechanical properties of PHU-based vitrimers and their recycling conditions. Improving mechanical properties, often associated with higher *T*_g_, generally results in more demanding recycling conditions, requiring elevated temperatures and extended processing times.

**Figure 2:**
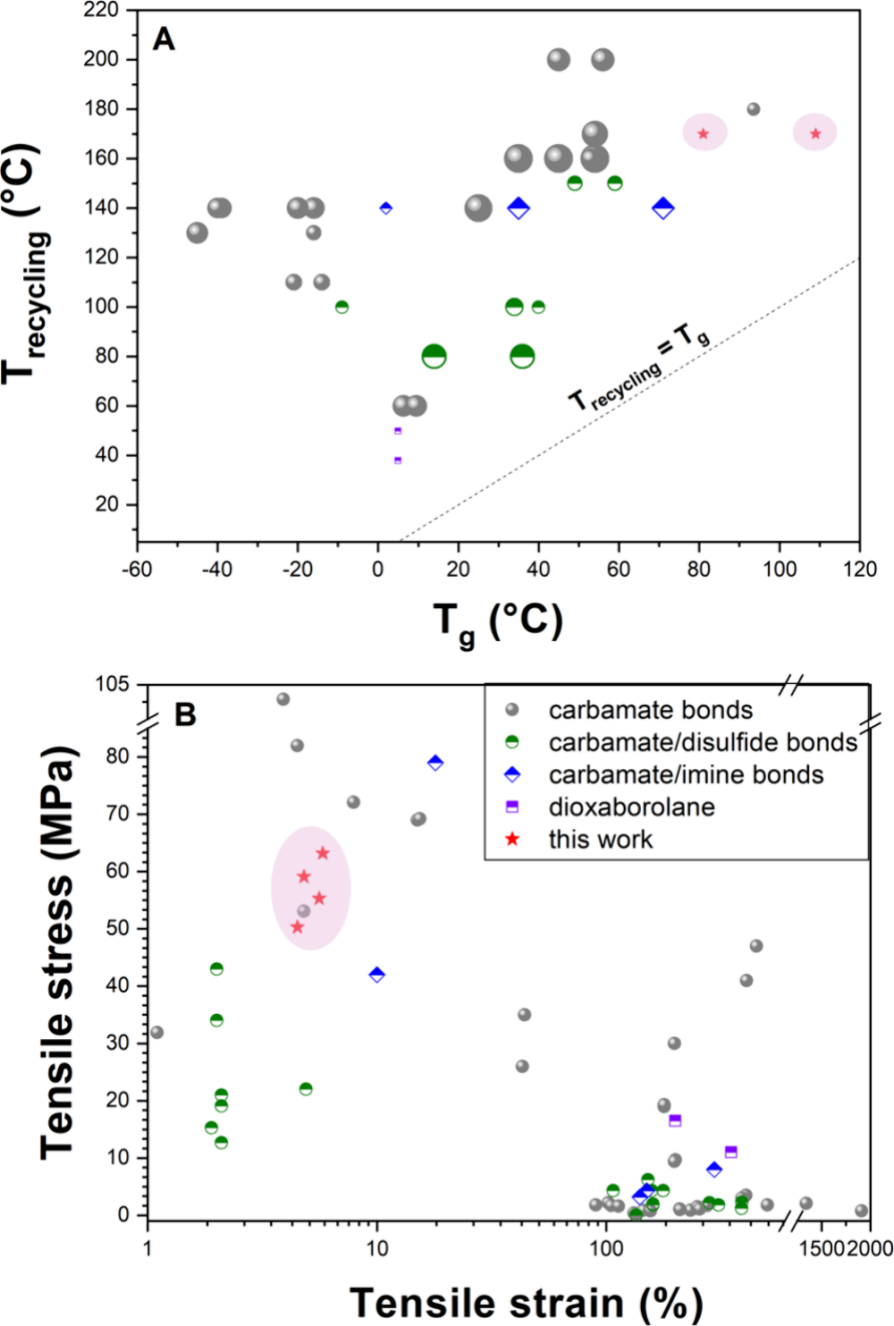
Recycling conditions vs *T*_g_ (A) and tensile properties (B) of PHU-based vitrimers (the size of the symbol is proportional to the logarithm of the recycling time at the specific temperature).

The introduction of disulfide bonds^33–37,41^ (Figure 1B) further enhances the rate of bond exchange, thereby improving the processability of these networks (Table S5). However, disulfide exchange has several disadvantages: a low activation energy (*E*_a_), resulting in extremely fast exchange within the temperature range of −13 to 30 °C; difficulty in handling precursors due to their high melting points; and a tendency to undergo oxidation, which is accelerated by light and heat, thereby deactivating the exchange. Because disulfide exchange occurs at relatively low temperatures, PHU networks with *T*_g_ values below 35 °C (Table S5, entries 16 and 18; Figure 2) can be readily reprocessed at moderately elevated temperatures (80–100 °C). However, at room temperature, these materials exhibit low tensile strength (1–2 MPa) and high elongation at break (150–390%).^36,37^ Increasing the *T*_g_ to 44–59 °C effectively suppresses disulfide exchange at room temperature while significantly enhancing the mechanical properties of the vitrimers, achieving tensile strengths of 13–43 MPa and reduced elongation at break (1.0–2.5%) (Table S5, entries 15 and 17).^33,35^ Reprocessing at 80 °C in this case requires extended times (up to 240 min), whereas raising the temperature to 150 °C enables much faster reprocessing, within 30 min.

To date, only two studies have focused on imine exchange in PHU-based vitrimers (Figure 1C).^34,38^ In the first report,^38^ the *E*_*a*_ values were generally lower (82–102 kJ·mol^–^^1^) compared to PHU vitrimers relying solely on transcarbamoylation (Table S5, entry 20). With *T*_g_ values ranging from 35 to 70 °C, these vitrimers exhibited tensile strengths between 8 and 79 MPa (Figure 2B). The incorporation of dual dynamic bonds reduced the reprocessing temperature to 140 °C and the time required to 60 min. The second study^34^ employed a combination of three dynamic bond types, namely, carbamate, disulfide, and imine, which made it challenging to clearly attribute the resulting properties to any single exchange mechanism.

An alternative approach to PHU vitrimers involves utilizing the pendant hydroxyl groups present in PHUs, which can react with 1,4-phenylenediboronic acid^42^ to form vitrimers with rapid dynamic dioxaborolane exchange^39^ (Figure 1D). This bond type is highly reversible and effective for creating strong vitrimers with good reprocessing capabilities, and has gaining popularity in high-performance polymer systems due to the balance between exchange rate and stability.^42^ In the given example (Table S5, entry 21), the vitrimers exhibited *T*_g_ values in the range of 38–50 °C and relatively high tensile strengths of 11–16.5 MPa (Figure 2B). Notably, these materials can undergo reprocessing at 120 °C within just 5 min. However, these dynamic bonds are highly sensitive to moisture, which can significantly influence the exchange rates and, consequently, the overall properties of the vitrimer network.^42^ This sensitivity compromises the overall stability and performance of the network, particularly at elevated humidity levels.^42,43^

These issues can be potentially avoided by using dioxazaborocane moieties, which are further stabilized by a dative N→B bond (Figure 1E). Dioxazaborocane-based vitrimers are a relatively new class of materials that exhibit dynamic covalent bonding based on boron-associated exchange reactions. They have recently gained attention for their interesting combination of mechanical properties, reprocessability, and stability.^44–51^ The use of such dynamic bonds has been previously reported for designing PU^44^ and polydicyclopentadiene^45^ vitrimers, as well as for modification and vitrimerization of linear epoxy-amine^46–49^ and linear benzoxazine^50,51^ polymers. It was reported^44^ that these moieties exhibit increased thermal and hydrolytic stability vs unstabilized boronate linkages; however, a comprehensive study comparing the differences between dioxaborolane and dioxazaborocane moieties based on both aliphatic and aromatic amines is still lacking. Likewise, we are aware of only a single report looking at the influence of 40% RH on a conventional isocyanate-based polyurethane vitrimer containing dioxazaborocane dynamic bonds.^44^ In contrast, to the best of our knowledge, the influence of moisture on the mechanical properties of nonisocyanate-based polyhydroxyurethane vitrimers containing such dynamic bonds, and their repeated mechanical and chemical recycling, have not been described in the literature.

This study aims to address the limitations of linear PHUs and existing PHU vitrimers by developing high-performance PHU vitrimers with an outstanding combination of physical and mechanical properties, making them competitive with conventional thermoplastic PUs. Here, we present for the first time a method for transforming tailored PHUs into mechanically robust PHU vitrimers using 1,4-phenylenediboronic acid as a dynamic cross-linker (Scheme 1). To elucidate the differences in stability between the resulting dioxaborolane (Scheme 1, **PHU_1_-V**) and dioxazaborocane moieties, which contain aliphatic and aromatic nitrogen donors (Scheme 1, **PHU_2_-V** and **PHU_3_-V**), we have conducted a comprehensive study of their structure using solid-state NMR and have evaluated their mechanical properties in the dry state and under varying humidity levels. Finally, we assessed their mechanical and chemical recyclability and demonstrated that these materials may be successfully recovered in a closed-loop manner.

**Scheme 1:**
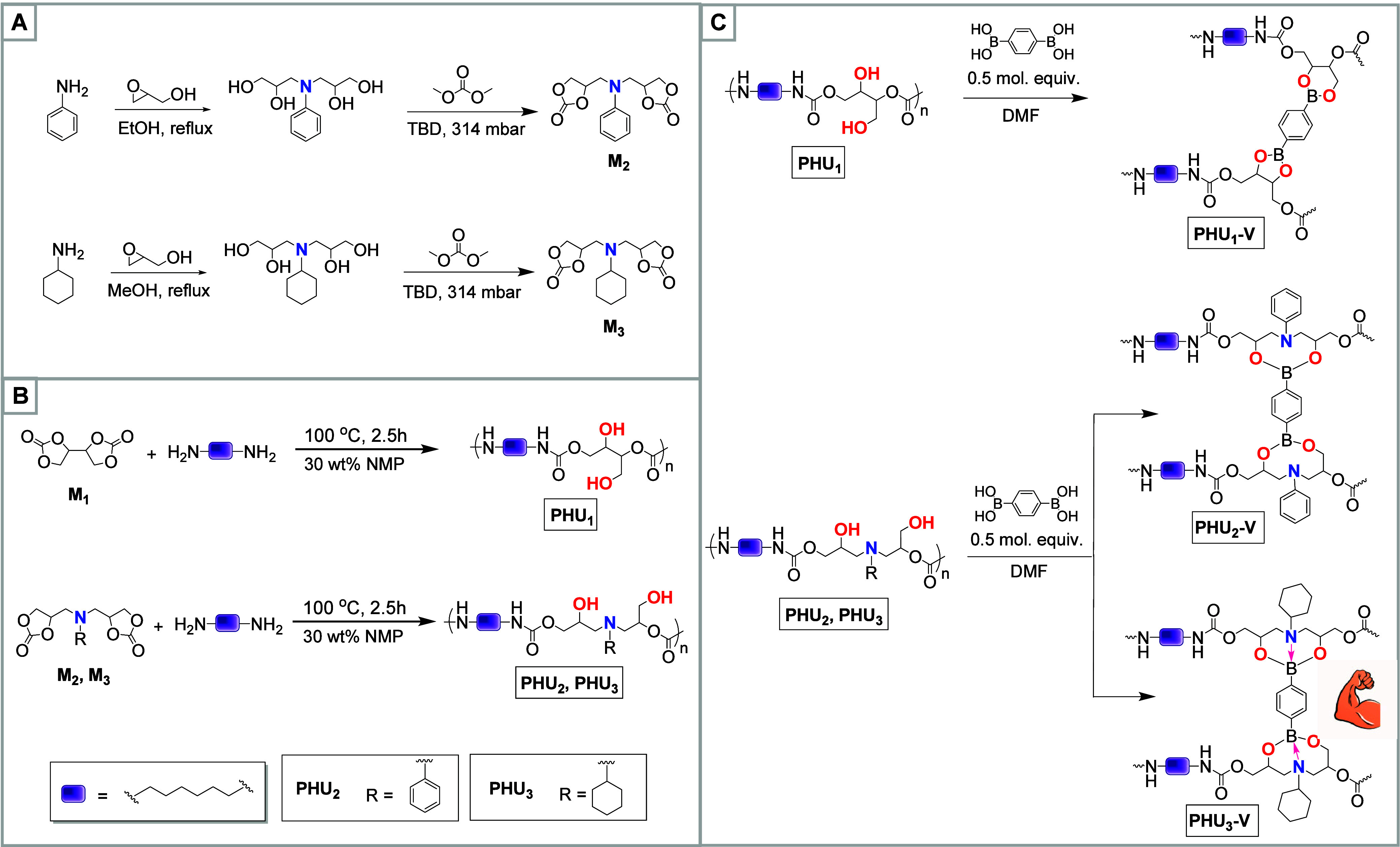
Synthesis of Compounds in the Current Study^a^

a (A) Synthesis of bis­(cyclic carbonate) monomers **M_2_** and **M_3_**; (B) synthesis of **PHU_1_**, **PHU** **_2_**, and **PHU_3_** from corresponding bis­(cyclic carbonate)­s and HMDA (formulas for PHUs are provided in a simplified manner, all possible structural units are shown on Schemes S3 and S4 in the Supporting Information); (C) synthesis of PHU-vitrimers via cross-linking of respective PHUs (with simplified formulas for PHUs) with 1,4-phenylenediboronic acid.

## Experimental Section

The list, origin, and purification of all materials, as well as detailed procedures for the synthesis and characterization of monomers with cyclic carbonates, and the preparation of PHU polymers and PHU vitrimers can be found in the Supporting Information (SI) file.

The Supporting Information file includes detailed characterization procedures and standards, together with NMR, DSC, DMTA, stress–strain, swelling, gel content, and water absorption data for PHU polymers and vitrimers; SEM images of milled vitrimer samples; results for mechanically and chemically recycled **PHU**_**3**_ **-V** specimens; and descriptions of the chemical recycling procedures.

## Results and Discussion

### Synthesis and Characterization

First, two new five-membered cyclic dicarbonate monomers **M**_**2**_ and **M**_**3**_ with a tertiary amine core were synthesized through a two-step process (Scheme 1A). In the first step, either aniline or cyclohexylamine was reacted with an excess of glycidol to form the corresponding tetrol intermediate. The product was then converted into a bis­(cyclic carbonate) following the procedure adapted from Dannecker and Meier.^52^ The structures and purity of the resultant monomers were confirmed using NMR (SI, Figures S1, S2, S4, S5) and elemental analysis.

Three polyhydroxyurethanes (**PHU**_**1**_, **PHU**_**2**_, and **PHU**_**3**_) were synthesized via reactive extrusion using hexamethylenediamine (HMDA) reacted with erythritol dicarbonate (**M**_**1**_), **M**_**2**_, or **M**_**3**_, respectively (Scheme 1B). Polymerization was carried out under optimized conditions: 100 °C, 2.5 h, with the addition of 30 wt % of *N*-methyl-2-pyrrolidone (NMP) as a solubilizing and hydrogen bond disrupting agent, as previously reported by our group.^53^ The resulting PHUs exhibited comparable molecular weights, increasing in the following order: **PHU**_**2**_ (*M*_n_ = 9500 g·mol^–1^; *M*_w_/*M*_n_ = 3.95) < **PHU**_**1**_ (*M*_n_ = 13600 g·mol^–1^; *M*_w_/*M*_n_ = 7.8) < **PHU**_**3**_ (*M*_n_ = 16900 g·mol^–1^; *M*_w_/*M*_n_ = 2.5). Analysis of PHU microstructures by ^1^H NMR revealed similar ratios of primary to secondary hydroxyl groups for **PHU**_**2**_ (OH­(I):OH­(II) = 39:61, SI, Figure S8) and **PHU**_**3**_ (OH­(I):OH­(II) = 37:63, SI, Figure S12). This contrasts with the lower ratio observed in **PHU**_**1**_ (OH­(I):OH­(II) = 25:75)^53^ as well as other PHUs (OH­(I):OH­(II) = 24:76)^54^ prepared via similar methods. This difference may be attributed to the influence of neighboring tertiary nitrogen atom present in **M**_**2**_ and **M**_**3**_, which may alter the electron density of the cyclocarbonate moieties or act as an internal catalyst,^55^ thus affecting the aminolysis reaction.^56^ Also of note, the formation of urea moieties^57,58^ during polymerization was not observed for **PHU**_**2**_ or **PHU**_**3**_ (SI, Figures S8 and S12); in contrast, in **PHU**_**1**_, their concentration was estimated to be ∼1.5 mol %.^53^

In the next step, the **PHU**_**x**_ polymers were dissolved in dimethylformamide (DMF) and cross-linked by adding 0.5 mol equiv. of 1,4-phenylenediboronic acid to form the corresponding **PHU**_**x**_ **-V** vitrimers (Scheme 1C). The molar ratio between the reactive functional groups of the polymers (assuming two OH groups per repeating unit) and the cross-linker was maintained at 1:1 to achieve the maximum theoretical cross-link density. The resulting mixtures were thoroughly dried first in air at 60 °C for 1 h and then at 80 °C for 1 h. Afterward, they were subjected to dynamic vacuum (0.5 mbar) at 100 °C for 10 h and finally at 120 °C for 5 days to ensure the complete removal of all volatile impurities.

In order to elucidate the chemical structure of the networks and to directly observe the effect of N-stabilization of dioxazaborocane moieties, **PHU**_**1**_ **-V**, **PHU**_**2**_ **-V**, and **PHU**_**3**_ **-V** vitrimers were studied using multinuclear natural abundance ^13^C, ^15^N, and ^11^B solid-state magic-angle-spinning (MAS) NMR.^59^ First, natural abundance (1.08 at. %) ^13^C CP-MAS NMR spectra confirmed the presence of 1,4-phenylenediboronic acid in those materials to which it was added. The resonance lines at 133–134 ppm, attributed to the aromatic carbons of the cross-linker, are present in the spectra of **PHU**_**1**_ **-V** (Figures S29 and S30 in the SI), **PHU**_**2**_ **-V** (Figures S33 and S34), and **PHU**_**3**_ **-V** (Figure S37), but are notably absent in the spectra of the corresponding non-cross-linked polymers: **PHU_1_** (Figure S21), **PHU_2_** (Figures S23 and S24), and **PHU_3_** (Figure S26). Moreover, the ^13^C chemical shifts of the 1,4-phenylenediboronic acid in **PHU**_**1**_ **-V**, **PHU**_**2**_ **-V**, and **PHU**_**3**_ **-V** are approximately 0.6, 0.8, and 1.5 ppm shifted (more shielded C-sites), respectively, compared to the chemical shift of the neat reagent (134.7 ppm, Figures S18–S20), consistent with chemical bond formation. Interestingly, the carbonyl carbon sites of the main polymer chain, assigned to the resonance line at 157.7 ppm in **PHU_1_**, **PHU_2_**, and **PHU_3_** polymers, exhibit an additional shielding of ca. 0.5–0.7 ppm in the corresponding vitrimers as well. Additionally, a minor carbonyl carbon resonance at 163.1 ppm (in **PHU**_**1**_ **-V**) and 163.3 ppm (in **PHU**_**2**_ **-V** and **PHU**_**3**_ **-V**) most likely corresponds to >C=O groups hydrogen-bonded with HN< groups in the nearby polymer chains after cross-linking, reminiscent of hydrogen bonding in polyurethanes,^60^ polyamides^61^ and peptides and proteins.^62^ A corresponding minor resonance line for >NH groups hydrogen-bonded with the aforementioned carbonyl carbons was also observed in natural abundance (0.38 at. %) ^15^N CP-MAS NMR spectra of the vitrimers: Note a resonance line at δ­(^15^N) = 68 ppm (Figure 3) adjacent to the major resonance line at 44.8 ppm, which is present in all polymers and vitrimers studied (Figure 3). Dative N→B bonds, which further stabilize the cross-linked structures in vitrimers, were detected only in the **PHU**_**3**_ **-V** vitrimer. This was evidenced by the decrease in the relative integral intensity of the N-*c*-hex site at −2.4 ppm from 1.0 in PHU_3_ to 0.33 (representing unreacted groups) in **PHU**_**3**_ **-V**, along with the appearance of two new resonance lines at 22.3 and 36.2 ppm, with relative integral intensities of 0.23 and 0.44, respectively, in the ^15^N CP-MAS NMR spectrum of this vitrimer. Given that the ratio of primary and secondary hydroxyl groups in **PHU**_**3**_ was found to be (OH­(I):OH­(II) = 37:63) and the ratio of integral intensities of resonances at 22.3 and 36.2 ppm is equal to 0.23:0.44 = 34:66, we hypothesize that these resonances correspond to structures with varying strengths of dative N→B bonds. These differences in deshielding effects on the nitrogen atoms caused by the chemical bond with boron are likely governed by steric effects given the potential to form different moieties based on primary and/or secondary hydroxyl groups during cross-linking of **PHU**_**3**_. The solid-state ^11^B MAS NMR spectrum of **PHU**_**3**_ **-V** revealed a line shape characteristic of quadrupolar nuclei (*I* = 3/2), which was not fully averaged out by MAS (Figure S36 in the SI). This line shape was narrower compared to the other systems in this study, with a distinct feature near 6 ppm, characteristic of tetrahedrally coordinated boron sites. This observation indirectly supports the hypothesis of a dative N→B bond formation in **PHU**_**3**_ **-V** as well.

**Figure 3:**
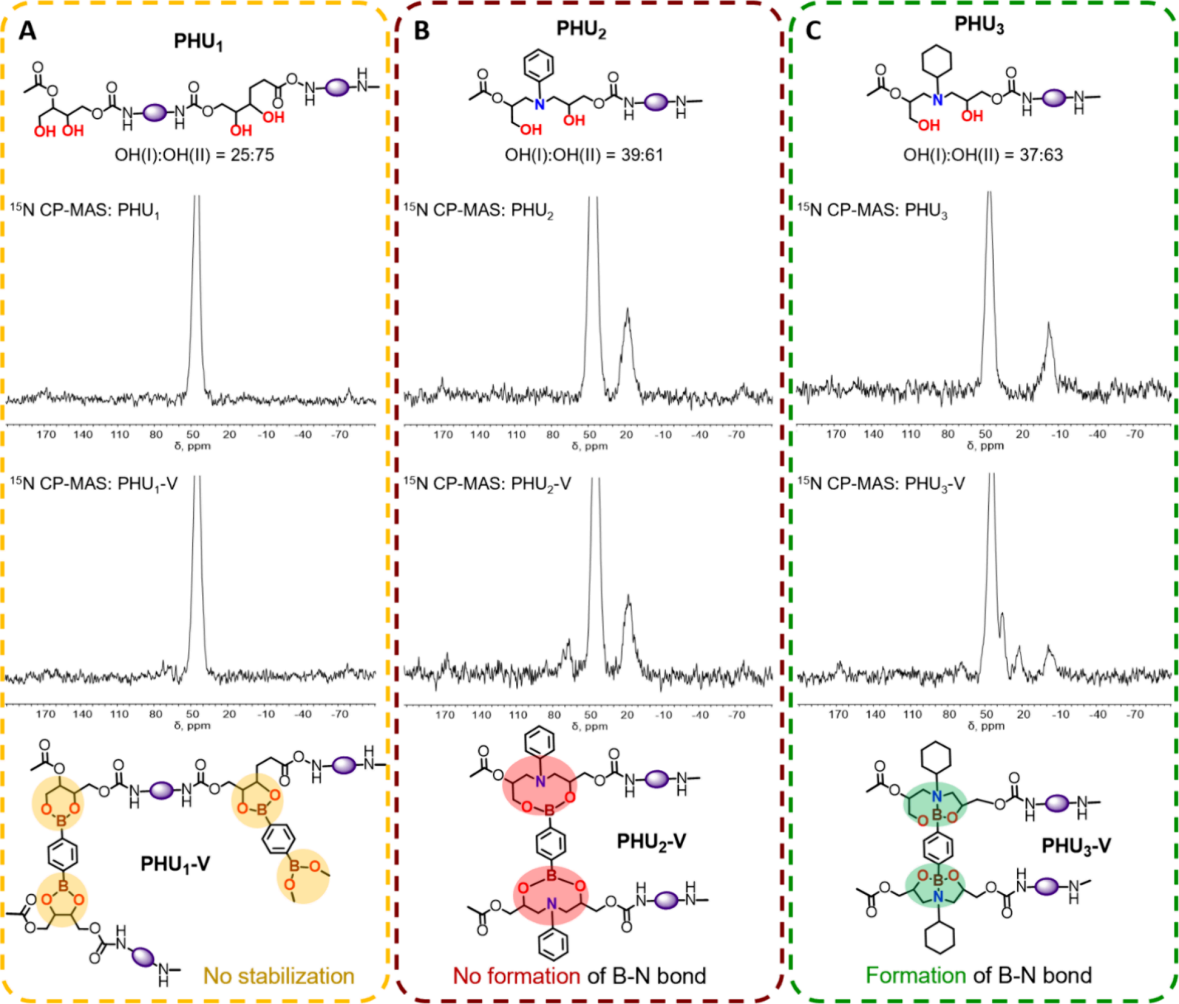
^15^N CP-MAS NMR spectra and structural features of **PHU_1_** and **PHU_1_-V** (A), **PHU_2_** and **PHU_2_-V** (B), and **PHU_3_** and **PHU_3_-V** (C).

### Thermomechanical Properties

All newly synthesized PHU polymers and vitrimers were characterized (Table 1) using DSC (Figures S39–S43) and TGA methods. The characterization of **PHU**_**1**_ was previously reported by our group;^53^ the same data are reused here for comparison purposes.

**Table 1:** Properties of **PHU**_**x**_ **-V** Vitrimers versus Pristine **PHU**_**x**_ Polymers

	powders		melt processed bars								melt processed dumbbells		
					water uptake vs RH (wt %)^d^			G’ vs RH(MPa)^e^					
sample	*T*_g_ (°C)^a^	*T*_onset_(°C)^b^	*T*_α_ (°C)^c^	E’ @ RT(MPa)^c^	45%	65%	85%	45%	65%	85%	σ_t_(MPa)^f^	ε_t_(%)^f^	E(MPa)^f^
PHU_1_ ^g^	50^h^	185	69	1850	2.0	3.7	7.5	1600	360	54	20.7 ± 1.1	1.8 ± 0.3	1580 ± 45
PHU_2_	53	175	68	1220	0.9	1.9	4.8	1020	628	307	21.8 ± 1.4	1.2 ± 0.1	2020 ± 60
PHU_3_	47	175	63	1670	1.0	2.6	4.6	710	363	192	33.6 ± 2.7	2.5 ± 0.3	1700 ± 45
PHU_1_–V	80	175	130	1440	2.8	5.4	9.2	817	688	365	50.3 ± 4.2	4.5 ± 0.1	1375 ± 65
PHU_2_–V	81	180	121	1920	3.4	5.6	9.9	844	584	360	59.1 ± 4.3	4.8 ± 0.1	1510 ± 125
PHU_3_–V	109	175	167	1060	3.0	5.4	8.8	782	743	560	55.3 ± 2.3	5.6 ± 0.6	1170 ± 35

a By DSC at a heating rate of 5 °C/min.

b By TGA in air at a heating rate of 5 °C/min.

c Storage modulus (*E*’) at 25 °C and *T*_α_ (as determined by the maximum in the loss modulus (*E*’’) curve) measured using DMTA at a heating rate of 3 °C/min and 1 Hz frequency.

d Determined after 14 days of conditioning at a selected humidity level.

e Storage modulus (*G*’) at 0.1 Hz measured and 0.1% shear strain via torsional rheometry after conditioning at a selected humidity level and 22 °C for 3 days.

f Tensile strength (σ_t_), elongation at break (ε_t_), and Young’s modulus (*E*).

g For comparison, data from prior work.^53^

h Two melting peaks with *T*_m_ = 97 °C and 130 °C were also observed and were attributed to the 14.8% degree of crystallinity estimated via DSC,^53^ and *T*_m_ was taken as peak maximum.

Thermal stability was found to be similar for all PHU polymers and vitrimers, with *T*_*onset*_ point ranging between 175 and 185 °C (Table 1). The glass transition temperature (*T*_g_) of **PHU**_**2**_ was slightly higher than that of **PHU**_**1**_ (53 °C for PHU_2_ vs. 50 °C for PHU_1_), likely due to the presence of rigid phenyl rings attached to the polymer backbone. In contrast, the *T*_g_ of **PHU**_**3**_ was the lowest among the three PHUs (47 °C), which can be attributed to the presence of a flexible cyclohexyl moiety. Both **PHU**_**2**_ and **PHU**_**3**_ were completely amorphous, while **PHU**_**1**_ exhibited two melting peaks and crystallinity of 14.8% when characterized via DSC in powder form.^53^

Cross-linking PHU polymers with 1,4-phenylenediboronic acid significantly increased the *T*_g_ values of the resulting vitrimers – by approximately 30 °C for **PHU**_**1**_ **-V** and **PHU**_**2**_ **-V**, and by 62 °C for **PHU**_**3**_ **-V**, compared to the original PHU polymers. The substantial difference in *T*_g_ between **PHU**_**2**_ **-V** and **PHU**_**3**_ **-V** may serve as indirect evidence of the formation of dative N→B bonds, leading to the formation of more rigid dioxazaborocanes in **PHU**_**3**_ **-V**. This stabilization, however, was not observed in **PHU**_**2**_ **-V**, as confirmed by solid state NMR.

The synthesized PHU vitrimers were ball-milled (estimates of average PHU vitrimer particles size by SEM are provided in Table S2 and Figures S45–S47). Subsequently, PHU polymers and ball-milled PHU vitrimers were hot-pressed into bar-shaped specimens (approximate length × width × thickness = 20 × 5 × 1 mm; see the SI) and analyzed by DMTA in tension mode (Table 1, Figure S48). The observed *T*_α_ values (as determined by the maximum in the loss modulus (*E*’’) curve) were following the same tendency as the *T*_g_ values obtained by DSC. The *T*_α_ values for **PHU**_**1**_ (69 °C) and **PHU**_**2**_ (68 °C) were almost identical, while **PHU**_**3**_ exhibited a lower *T*_α_ of 63 °C, which was ascribed to the presence of the more flexible cyclohexyl pendant groups. For the vitrimer samples, the *T*_α_ values increased in the following order: **PHU**_**2**_ **-V** (121 °C) < **PHU**_**1**_ **-V** (130 °C) ≪ **PHU**_**3**_ **-V** (167 °C). This trend aligns with the reduction in size of the dioxaborolane cycles, from 9/10-membered in **PHU**_**2**_ **-V** to two connected 5/6-membered cycles in **PHU**_**3**_ **-V** (Figure 4A), resulting in an overall decrease in chain mobility within the network.

**Figure 4:**
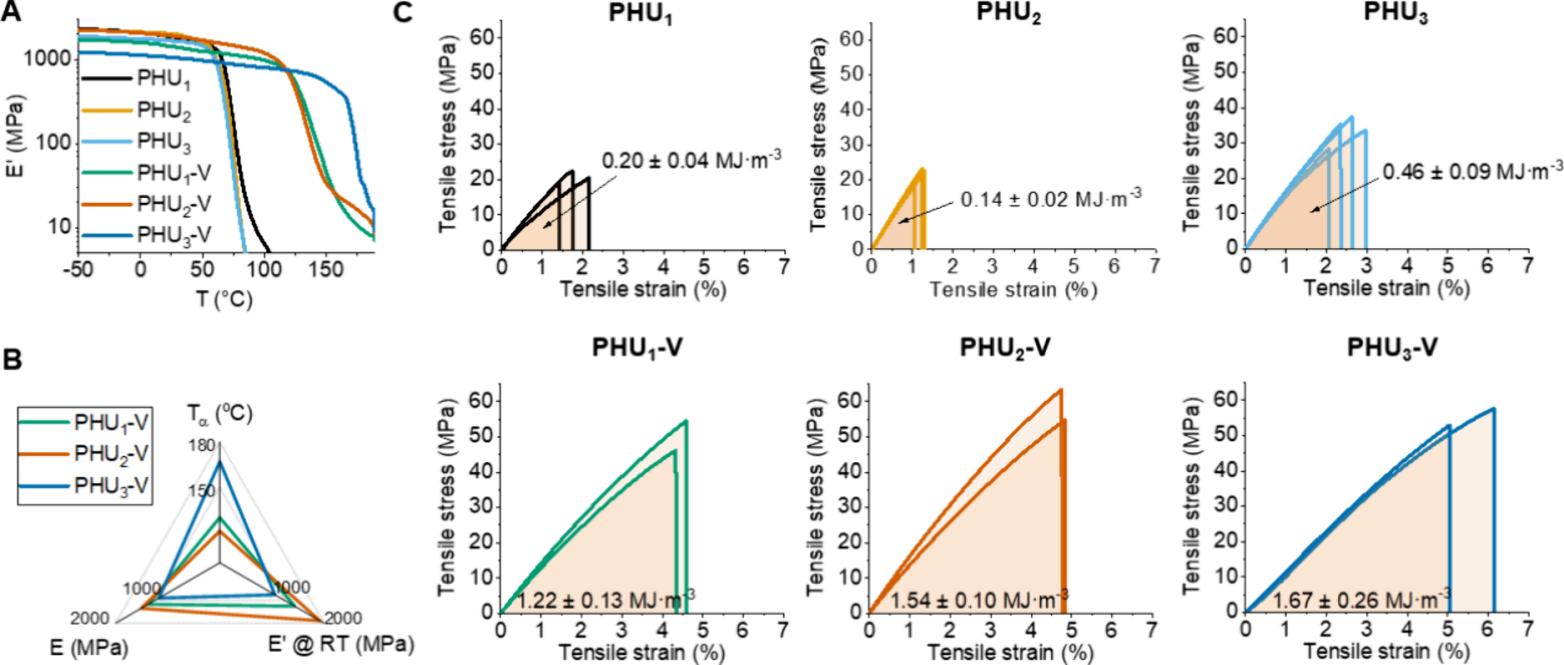
(A) Storage moduli (*E*’) for PHU_*x*_ polymers and corresponding PHU_*x*_-V vitrimers measured by DMTA; (B) comparison of thermomechanical properties of PHU_*x*_-V vitrimers and (C) tensile stress–strain plots for PHU_*x*_ polymers and corresponding PHU_*x*_-V vitrimers.

The stiffness of these materials at room temperature was evaluated using storage modulus values measured by DMTA (Figure 4A, Figure S48) and also via tensile measurements performed using dumbbells (ISO 37 type 4) (Figure 4C), with all values reported in Table 1. Considering this set of data as a whole, two observations may be made. When considering the **PHU**_**x**_ series, the stiffness trends are reversed depending on which set of data is considered. In contrast, when considering the **PHU**_**x**_ **-V** series, it is consistently observed that the stiffness of these materials goes as **PHU**_**2**_ **-V** > **PHU**_**1**_ **-V** > **PHU**_**3**_ **-V**. These results further emphasize the rationale behind the work: in the case of the **PHU**_**x**_ series, the combination of low glass transition temperatures and significant water absorption renders the stiffnesses of these materials exceptionally sensitive to small changes in water content and highly inconsistent as a result. In contrast, in the **PHU**_**x**_ **-V** series, while the materials remain capable of absorbing water, their much higher glass transition temperatures ensure that their stiffnesses at room temperature are much less affected. When comparing the vitrimers with their parent polymers, a third trend becomes apparent – namely, a tendency to observe a reduction in room temperature stiffness upon vitrimer formation. While this might seem counterintuitive initially, this can be explained by significant reductions in intermolecular hydrogen bonding as the hydroxyl groups are effectively consumed during the formation of dioxaborolane/dioxazaborocane cycles. The expected decrease in solubility parameter and cohesive energy density and the observed decrease in glassy modulus are consistent with prior observations linking the two, both for amorphous polymers in general^63^ and cross-linked networks with similar concentrations of hydroxyl groups in particular.^64^

Having dealt with the question of stiffness, we turn now to the tensile strength (σ_*t*_) and elongation at the break (ε_*t*_). Here, all vitrimers exhibited σ_*t*_ values within the range 50–60 MPa, while ε_*t*_ values varied in the range of 4–6%. In contrast, the neat PHUs showed significantly lower σ_*t*_ (20–34 MPa) and ε_*t*_ (1–2.5%) values. Tensile toughness, measured by integrating the area under the stress–strain curves, also increased with the cross-linking of PHUs. The most significant increase in tensile toughness was observed in the **PHU**_**2**_ **/PHU**_**2**_ **-V** system, where the formation of stabilized dioxazaborocanes in **PHU**_**2**_ **-V** gave a tensile toughness ∼11 times higher than the parent **PHU**_**2**_. In contrast, the other vitrimers showed increases in tensile toughness of ∼4–6 times that of the parent PHUs. Moreover, a comparison of the tensile properties of the vitrimers developed in this study, particularly **PHU**_**2**_ **-V** and **PHU**_**3**_ **-V**, with previously reported PHU-based vitrimers (Table S5 and Figure 2B) clearly demonstrates their superior performance. With tensile strengths in the range of 50–60 MPa and low elongation at break values (4–6%), these materials stand out as among the top three highest-performing PHU vitrimers reported to date. These notable improvements in mechanical properties in the PHU-based vitrimers position them as a promising matrix material for future composite applications^65,66^ while highlighting the benefits of stabilized dioxazaborocane formation in particular.

### Viscoelastic Properties and Water Absorption at Various Humidity Levels

One of the significant drawbacks of nonstabilized dioxaborolane moieties is their moisture sensitivity. To investigate how dioxazaborocane formation and N→B stabilization affect the moisture sensitivity of PHU vitrimers, a detailed study was conducted of their water absorption and mechanical properties as a function of humidity. The results were compared with those of the original PHU polymers.

First, all materials were conditioned for 14 days at three different humidity levels – 45, 65, and 85% relative humidity (RH). Water uptake (by weight) was measured periodically (Table 1, Figure S44). The results showed that **PHU**_**2**_ and **PHU**_**3**_ absorbed less water (4.8 and 4.6 wt %, respectively) compared to **PHU**_**1**_ (7.5 wt %). This decrease in water uptake can be attributed to the structural differences in **PHU**_**2**_ and **PHU**_**3**_, where the lower density of hydroxyl groups (∼5 mmol of OH/g in both cases), separated by *N*-alkyl or *N*-aryl moieties, reduces water absorption compared to the more hydroxyl-rich structure of **PHU**_**1**_ (∼7 mmol of OH/g) (see SI).

PHU-vitrimers exhibited more consistent water uptake behavior compared with linear polymers. When vitrimer samples were conditioned at 85% RH for 14 days, water uptake ranged from 8.8 wt % (for **PHU**_**3**_ **-V**) to 9.9 wt % (for **PHU**_**2**_ **-V**). These higher values compared to the parent PHUs are likely due to the dioxaborolane and dioxazaborocane moieties, whose polar, hydrogen bond accepting nature would be expected to facilitate additional water uptake.

The room temperature (25 °C) moduli of PHU polymers and vitrimers were studied using torsional rheometry (Figure 5A) after conditioning them for 3 days at three different humidity levels – 45, 65, and 85% RH. The storage modulus (*G*’) values of pristine PHU polymers showed a significant decrease with increasing humidity. The most pronounced effect was observed for **PHU**_**1**_, where the storage modulus was reduced by nearly 2 orders of magnitude when moving from 45 to 85% RH. For **PHU**_**2**_ and **PHU**_**3**_, the decrease of storage modulus was only 3 to 3.5-fold when moving from 45 to 85% RH. These results align with the greater hydroxyl content and moisture uptake associated with **PHU**_**1**_ than with other members of the PHU series, which led to the greatest degree of hydro-plasticization of the polymer (Table S1).

**Figure 5:**
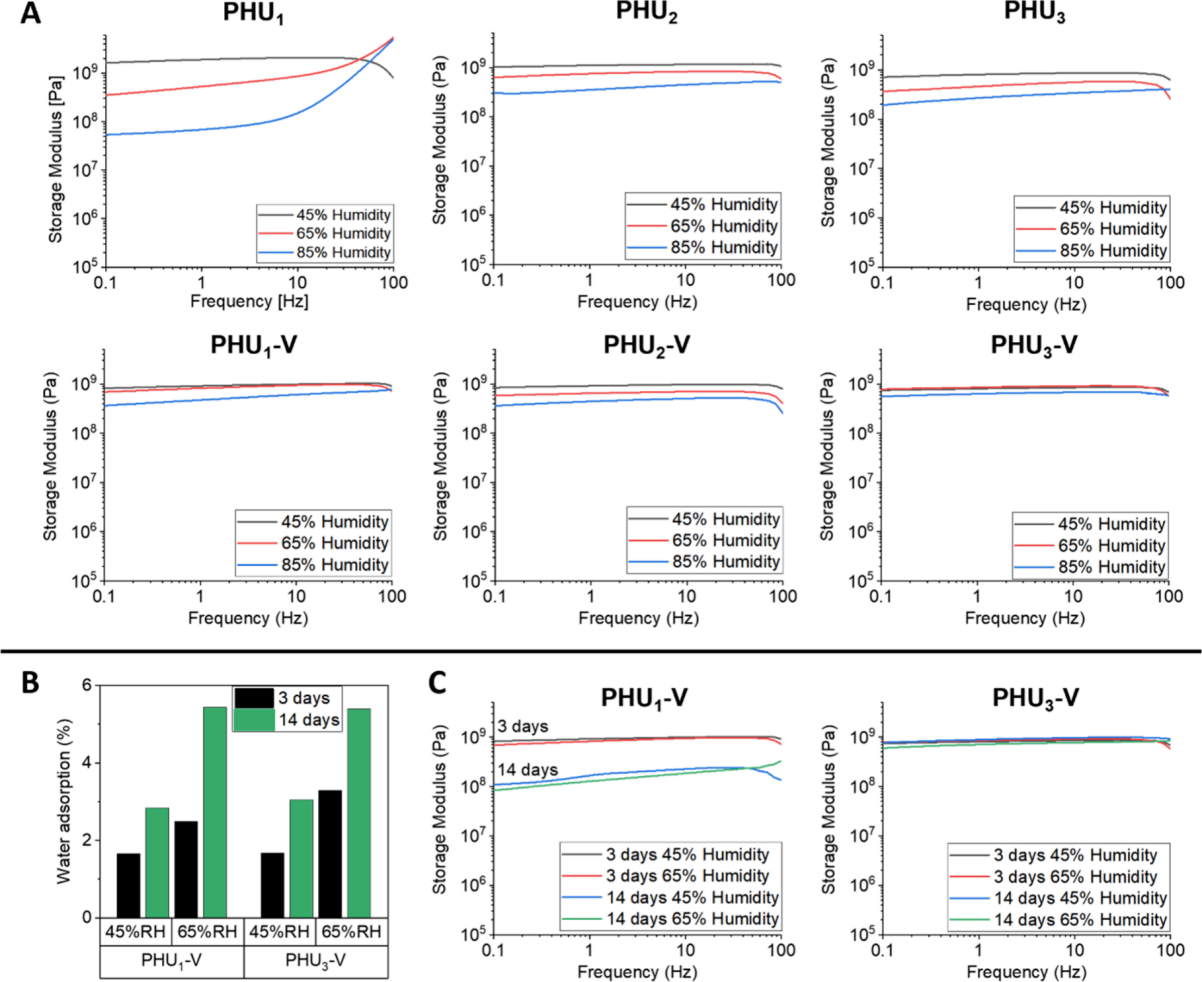
(A) Frequency dependence of storage moduli (G) at 25 °C for PHU_*x*_ polymers and PHU_*x*_-V vitrimers measured following conditioning at different humidity levels for 72 h; (B) water uptake of **PHU_1_-V** and **PHU_3_-V** after 72 h and 14 days of conditioning at 45% and 65% relative humidity (RH); (C) frequency dependence of storage moduli (*G*’) at 25 °C for **PHU_1_-V** and **PHU_3_-V** measured after conditioning at 45% and 65% relative humidity (RH) for 72 h and 14 days.

In contrast, the room temperature moduli of PHU vitrimers showed much less sensitivity to moisture uptake at elevated humidity levels (Figure 5A). While the vitrimer specimens absorbed more moisture compared to their thermoplastic counterparts (20% more for **PHU**_**1**_ **-V** vs **PHU**_**1**_ and 100% more for **PHU**_**2**_ **-V** vs **PHU**_**2**_ at 85% RH after 14 days), their stiffness was much less affected. For instance, only a 2-fold decrease in storage modulus was observed when humidity increased from 45 to 85% RH, a modest decrease considering the amount of moisture absorbed. Even more notably, the N-stabilized **PHU**_**3**_ **-V** exhibited the smallest reduction in mechanical performance among the materials studied, with its storage modulus dropping by just 30% between 45% and 85% RH. This suggests that the vitrimer networks are better able to retain their mechanical strength under moist conditions compared to their thermoplastic counterparts. This is likely explained by the higher glass transition temperatures of the vitrimer networks, which are expected to remain well above room temperature even following significant water uptake (8.8–9.9 wt %), as borne via predictions based on the Fox equation (see Table S1).

Our previous report showed that 3 days of conditioning prior to rheological measurements was sufficient to reach near- equilibrium conditions for thermoplastic samples.^53^ PHU vitrimers, however, exhibited different behavior, as water uptake significantly increased both for the nonstabilized (**PHU**_**1**_ **-V**) and N-stabilized (**PHU**_**3**_ **-V**) networks after 14 days of conditioning (Figure 5B). The degree and rate of water uptake were similar for all PHU networks (Table 1, Figure 5B, and Figure S44). Despite this, rheological measurements revealed significant differences in the mechanical properties of **PHU**_**1**_ **-V** and **PHU**_**3**_ **-V** after 14 days of conditioning compared to 3 days (Figure 5C). In particular, **PHU**_**1**_ **-V** showed a decrease in storage modulus of about 1 order of magnitude for samples conditioned at both 45 and 65% RH. In contrast, **PHU**_**3**_ **-V** exhibited remarkable stability, with its storage modulus remaining virtually unchanged even after extended exposure to high humidity levels. This indicates that **PHU**_**3**_ **-V** is much more resistant to the effects of moisture, maintaining its mechanical integrity despite the absorbance of similar amounts of water. The stark difference in behavior between **PHU**_**1**_ **-V** and **PHU**_**3**_ **-V** provides further evidence that the **PHU**_**3**_ **-V** network, containing dioxazaborocane moieties stabilized by dative N→B bonds, has significantly greater stability and performance compared to **PHU**_**1**_ **-V**, which contains dioxaborolane moieties.

It was also important to assess the hydrolytic stability of the best performing **PHU**_**3**_ **-V** network. To investigate this, a **PHU**_**3**_-V film was immersed in boiling distilled water, and its appearance was monitored over the course of 1 h (Figure S55). After just 5 min in boiling water, the film began to lose its shape and became significantly elongated, likely due to the temperature approaching its glass transition point (*T*_g_ = 109 °C, Table 1) and, in part, to plasticization. However, even after 1 h of immersion, no mass loss was observed, the FTIR spectra (Figure S56) remained virtually unchanged, and the characteristic C=O stretching band from the urethane group at 1687 cm^–^^1^ was still present, indicating the excellent hydrolytic stability of stabilized **PHU**_**3**_ **-V** polymer.

### Stress–Relaxation Behavior of PHU Vitrimers

To investigate the vitrimeric properties of the PHU networks described here, the stress–relaxation behavior was studied by using torsional rheometry on bar-shaped samples (approximate length × width × thickness = 20 × 5 × 1 mm), as shown in Figure 6.

**Figure 6:**
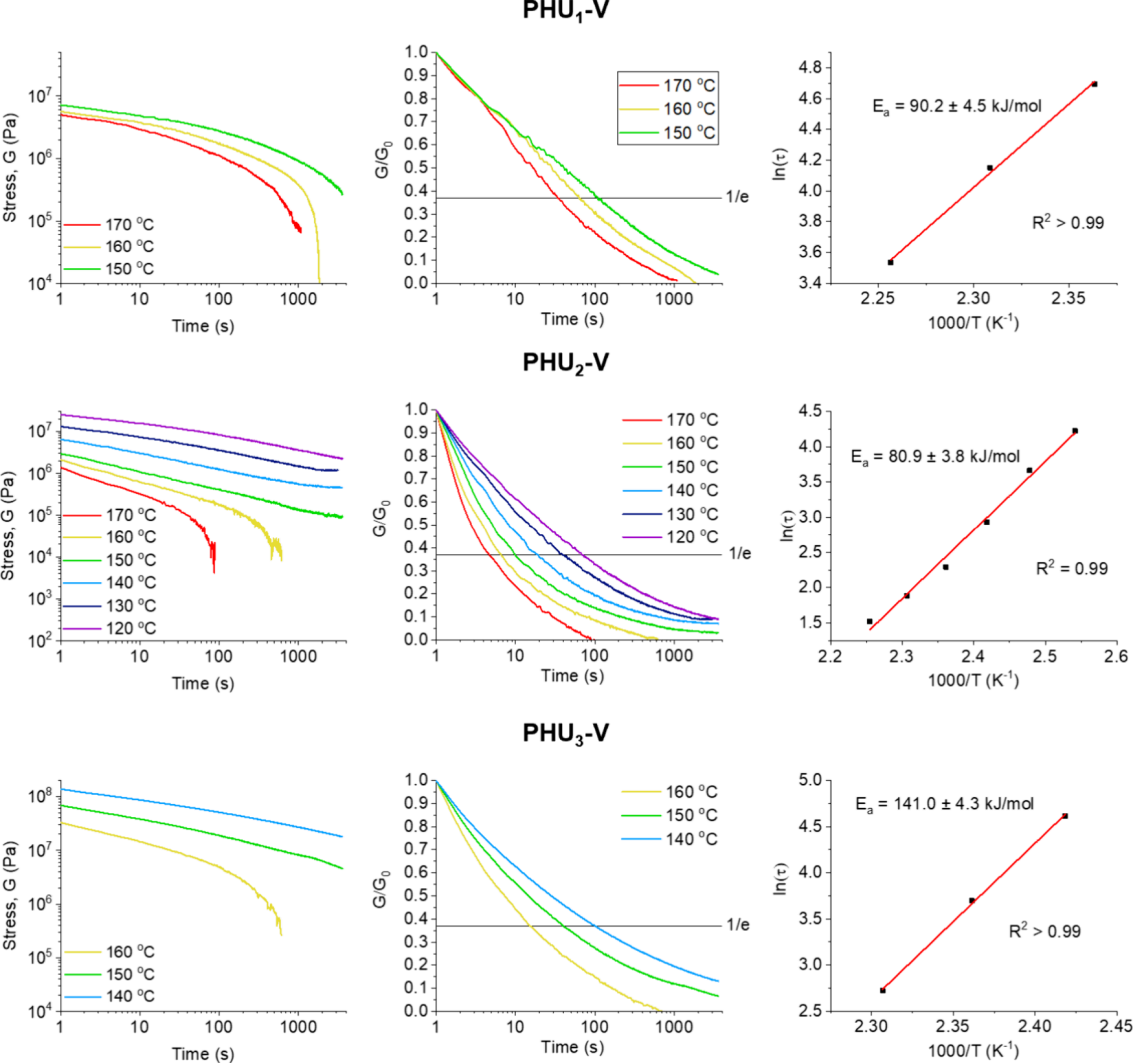
Non-normalized (left column) and normalized (center column) stress–relaxation plots at various temperatures; linear regression of the natural log of relaxation time versus inverse temperature (right column) for PHU_*x*_-V vitrimers.

As observed from the non-normalized plots (Figure 6, left column) and the plots normalized at 1 s after the start of the test (Figure 6, center column), all networks exhibited rapid stress relaxation across the full range of temperatures studied. The relaxation times (τ), determined at 37% (1/e) of the initial stress level at *t* = 1 s,^67^ decreased rapidly as the experimental temperature increased, indicating that higher temperatures facilitate stress relaxation through bond exchange processes. When comparing the networks at 160 °C, measured relaxation times increased in the following order: **PHU**_**2**_ **-V** (τ = 6.6 s) < **PHU**_**3**_ **-V** (τ = 15.2 s) ≪ **PHU**_**1**_ **-V** (τ = 63.5 s). The faster relaxation observed for **PHU**_**2**_ **-V** compared to that of **PHU**_**3**_ **-V** was ascribed to the structural difference in the dioxazaborocane moieties: in the case of **PHU**_**3**_ **-V**, the dioxazaborocane is stabilized by a nitrogen atom, reducing the rate of bond exchange and increasing the relaxation time. Unexpectedly, the slowest relaxation was observed for the nonstabilized **PHU**_**1**_ **-V**. The fact that **PHU**_**3**_ **-V** relaxes faster than **PHU**_**1**_ **-V** suggests that the stabilization mechanism introduced by the nitrogen atom in **PHU**_**3**_ **-V** may provide additional stability but only up to a certain temperature threshold. In this work, the stress–relaxation experiments were conducted at temperatures above 140 °C due to the high glass transition temperatures of the vitrimers studied. At these elevated temperatures, it is possible that the dative N→B bond in **PHU**_**3**_ **-V** significantly weakens^68^ or even dissociates, transforming the two rigid 5/6-membered cycles into one large 9/10-membered cycle, similar to that in **PHU**_**2**_ **-V** (Scheme 1C). This would explain why the relaxation times for **PHU**_**2**_ **-V** and **PHU**_**3**_ **-V** are quite similar, while **PHU**_**1**_ **-V** relaxes more slowly.

There are reports in the literature indicating that relaxation times for vitrimers based on dioxaborolane bonds can be significantly lower^43,69,70^ than those reported for **PHU**_**1**_ **-V** in this work. However, considering the hydrolytically unstable nature of these bonds, even traces of water can accelerate the bond exchange rate by reducing the cross-link density and activating an associative mechanism in addition to metathesis, which would otherwise be the only possible mechanism in a fully cross-linked network.

The activation energies (*E*_*a*_) for bond exchange reactions were calculated using an Arrhenius equation:

$$\ln\;\tau =\ln\;\tau_{0}+\frac{E_{a}}{RT}$$ 1

by plotting the natural logarithm of relaxation time (ln τ) vs 1000/T (Figure 6, right column). Here, τ_0_ describes the time between collisions of functional groups relevant for bond exchange, while *E*_*a*_ dictates the probability of thermally activated bond exchange once such a collision has occurred. The lowest *E*_*a*_ of 80.9 kJ/mol was observed for **PHU**_**2**_ **-V**, which is consistent with the presence of large, unstabilized dioxazaborocane moieties present in this material. While a relatively similar *E*_*a*_ value (90.2 kJ/mol) was observed for **PHU**_**1**_ **-V**, a much higher *E*_*a*_ value of 141 kJ/mol was observed for **PHU**_**3**_ **-V**, once more highlighting the significance of dioxazaborocane stabilization through the formation of N→B dative bonds. This value is in a perfect agreement with the activation energies reported for similar bonds in other vitrimer networks.^44,45^ Also of note are changes in the pre-exponential factor (τ_0_) in these systems. For **PHU**_**1**_ **-V** and **PHU**_**2**_ **-V**, the Arrhenius fits yield statistically indistinguishable (ln τ_0_) values of −20.9 ± 1.3 and −20.5 ± 1.1, respectively. In contrast, for **PHU**_**3**_ **-V**, the associated (ln τ_0_) value is −36.4 ± 1.2. While it has been demonstrated that changes in the concentration of dynamic bonds can directly impact τ_0_,^71^ the magnitude of the observed change is far too large to be explained by this factor alone, especially given the similar concentrations of dynamic bonds in all three systems.

Steric constraints have been implicated in changes in the pre-exponential factor in such systems,^72^ but here the change occurs in the opposite direction vs what would be expected when, in **PHU**_**3**_ **-V**, the formation of the N→B dative bond results in the conversion of a single 9/10 membered cycle into two 5/6 membered cycles. One possible explanation for this relates to the general observation that dative bonds in uncharged complexes tend to display increased stability as the polarity of their surroundings increases.^73^ In this view, low temperatures and in the presence of moisture render the environment more polar, enhancing charge transfer and stabilizing the N→B dative bond, thus counteracting the usual effects of water uptake on polymer properties. At temperatures relevant for bond exchange, on the other hand, the moisture in the system is driven away, the environment is significantly less polar, and the weakening of the N→B dative bond has an activating effect rather than a stabilizing effect, encouraging bond exchange by readily switching from one aliphatic nitrogen to another and significantly decreasing the pre-exponential factor in the process. While further work is needed to confirm “pre-exchange” of the aliphatic nitrogens, this view is in line with the aforementioned discussions concerning weakening of the N→B dative bond as an explanation for the trends in relaxation times of the **PHU**_**x**_ **-V** materials.

### Mechanical Recycling of PHU Vitrimers

Based on the analysis of mechanical properties, hydrolytic stability, and stress relaxation studies, **PHU**_**3**_ **-V** demonstrates high stability and strong potential for rapid recyclability at elevated temperatures. This formed the basis for its selection as the system for an investigation of mechanical recycling. Mechanical recycling studies were conducted consecutively using the same batch of vitrimer. At each stage, the dumbbells used for tensile testing (Figures 7A and 8A) were ball-milled in ambient conditions (25 ± 3 °C and 50 ± 5% RH) using a CryoMill. Estimates of average particle size by SEM are provided in Table S2 and Figure S47. After milling, the powder was dried under reduced pressure (0.5–1 mbar) at 100 °C for 24 h and then was reshaped into the desired form (dumbbell or bar) at 170 °C under 0.25 tons of pressure for 10 min. The bars used for DMTA analysis were excluded from the recycling process because they were heated to 190 °C during characterization, 20 °C higher than the recycling conditions and sufficient that degradation and/or the formation of permanent cross-links due to side reactions become concerns. In contrast, the recycled samples showed no change in color or clarity (Figure 8B).

**Figure 7:**
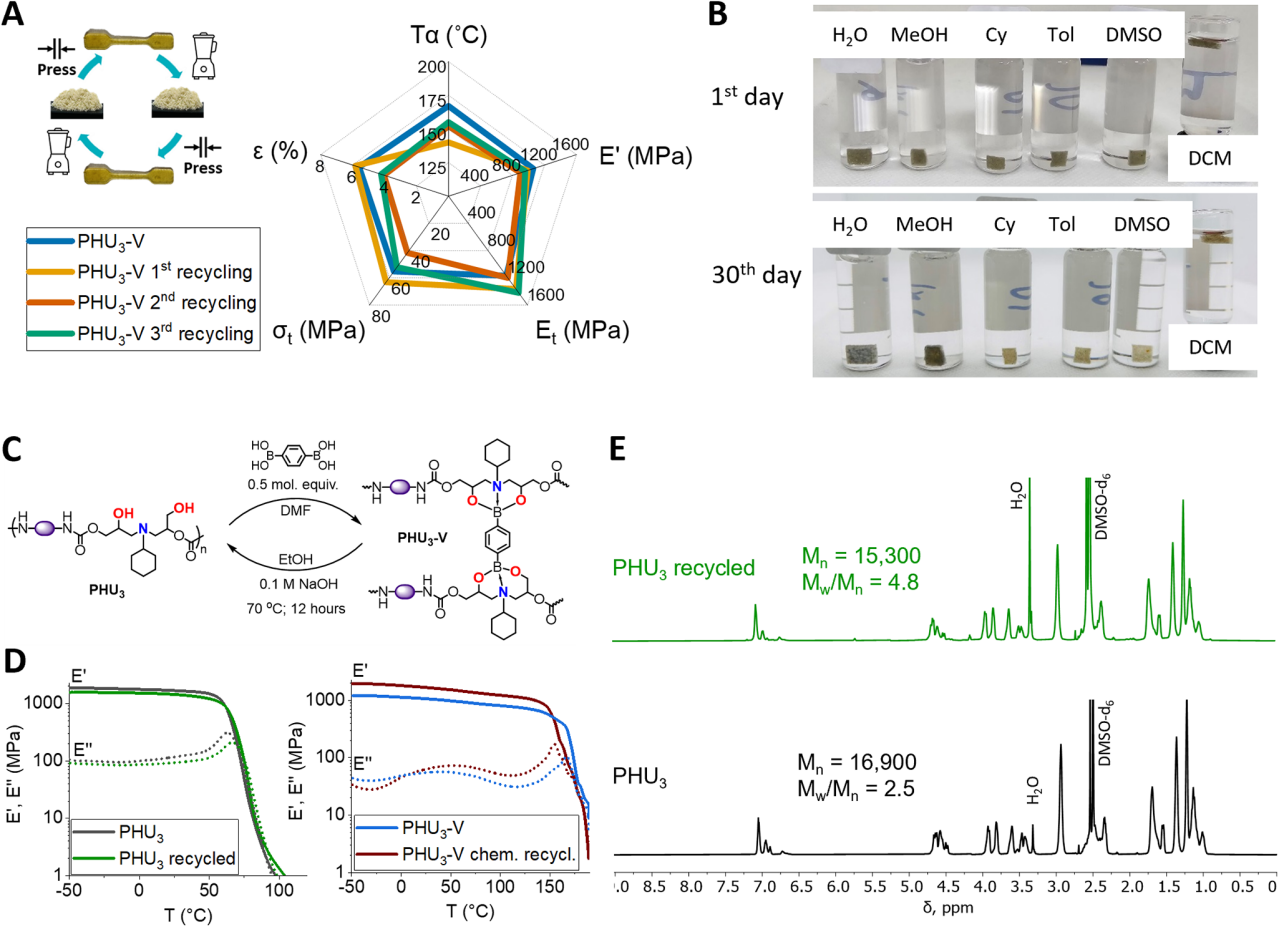
(A) Comparison of *T*_α_ values, storage moduli (*E’*) at 25 °C and 1 Hz frequency, Young’s moduli (*E*_*t*_), tensile strength (σ_*t*_) and elongation at break (ε_*t*_) for original and mechanically recycled PHU_3_–V; (B) photos of **PHU_3_-V** samples after 1 and 30 days of immersion in selected solvents (MeOH – methanol, Cy – cyclohexane, Tol – toluene, DMSO – dimethyl sulfoxide, DCM – dichloromethane); (C) scheme for chemical recycling of PHU_3_ polymer from PHU_3_–V and regeneration of PHU_3_–V vitrimer from the recycled materials; (D) storage (*E’*) and loss (*E’’*) moduli for original and chemically recycled PHU_3_, along with original and regenerated **PHU_3_-V**; (E) ^1^H NMR spectra of original and chemically recycled PHU_3_.

The properties of the mechanically recycled samples are summarized in Table 2. As shown by the DMTA data (Table 2, Figure S49), the recycled samples exhibited storage moduli very similar to those of the original samples, ranging from 900 to 990 MPa compared to 1060 MPa for the original **PHU**_**3**_ **-V** (Table 2, Figure 7A). The *T*_α_ value initially dropped to 140 °C after the first recycle, but subsequent recycling steps resulted in *T*_α_ values of 152 and 155 °C, respectively (Table 2, Figure 7A). This suggests that the structure and properties of these materials was maintained with little or no degradation occurring during the recycling process.

**Table 2:** Comparison of Properties of **PHU_3_-V** before and after Mechanical (at 170 °C) and Chemical Recycling

degree of recycling	*T*_α_ (°C)^c^	*E’* (MPa)^a^	σ_t_ (MPa)^b^	ε_t_ (%)^b^	*E* (MPa)^b^
original **PHU**_**3**_ **-V**	167	1060	55.3 ± 2.3	5.6 ± 0.6	1170 ± 3
1st mech. recycling	140	990	63.2 ± 8.6	5.8 ± 0.7	1365 ± 125
2nd mech. recycling	152	900	41.6 ± 5.2	4.0 ± 0.6	1200 ± 95
3rd mech. recycling	155	960	52.2 ± 5.3	4.2 ± 0.9	1420 ± 140
chemical recycling	156	1670	^c^	^c^	^c^

a Storage modulus (*E’*) at 25 °C and *T*_α_ (determined based on the maximum in the loss modulus (*E’’*) curve) measured via DMTA at heating rate of 3 °C/min and 1 Hz frequency;

b Tensile strength (σ_*t*_), elongation at break (ε_*t*_), and Young’s modulus (*E*) at 22 °C;

c Not determined.

**Figure 8:**
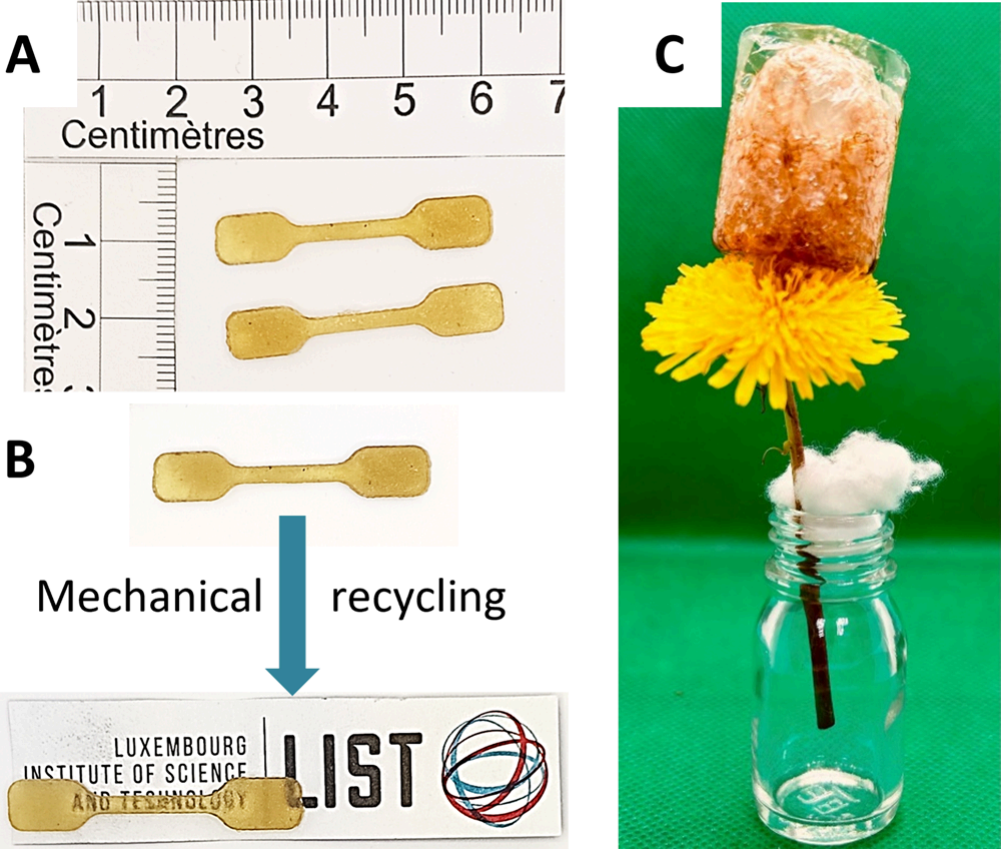
(A) Visual appearance of dumbbells prepared from **PHU_3_-V**; (B) visual appearance of dumbbells prepared from **PHU_3_-V** in its initial state and following the first recycle; (C) lightweight foamed **PHU_3_-V** sample placed on top of the dandelion. Logo is present with the permission of the Luxembourg Institute of Science and Technology (LIST) as the copyright holder.

The tensile properties were largely preserved over three mechanical recycling cycles as well (Table 2, Figures 7A and S49). The tensile strength (σ_*t*_) of the recycled samples after the first (63.2 ± 8.6 MPa) and third (52.2 ± 5.3 MPa) recycling cycles remained comparable to that of the original sample (55.3 ± 2.3 MPa). While Young’s moduli (*E*) varied slightly following the various recycling steps, they consistently ranged between 1200 and 1420 MPa and were always higher than the original sample’s value of 1170 MPa. The only parameter that decreased compared to the original sample was the elongation at break (ε_*t*_). After the first recycle, a value of 5.8 ± 0.7% was measured, statistically indistinguishable from the prerecycling value of 5.6 ± 0.6%. However, subsequent recycling steps gave similarly reduced values of 4.0 ± 0.6% and 4.2 ± 0.9%, respectively. One possible explanation for this would be small increases in cross-link density due to multiple heating cycles during the recycling process; this would explain the parallel increases observed in *T*_α_. A second possibility could be contamination of the material during multiple recycling processes, introducing flaws into the material and reducing elongation at break; this would explain the parallel decrease in tensile strength compared to the results obtained following the first recycling step. Nevertheless, these changes are sufficiently modest that, taken together, the presented data (Table 2, Figure 7A) indicate that **PHU**_**3**_ **-V** retains excellent recyclability for at least three cycles.

Reprocessing PHU-based vitrimers at 170 °C may potentially induce urethane bond exchange and promote the formation of urea bonds. To evaluate the possible formation of urea linkages under these conditions, infrared spectroscopy was conducted on the **PHU**_**3**_ **-V** system before mechanical recycling and after each of three recycling cycles (Figure S50). The FTIR spectra remained effectively unchanged throughout with the urethane peak (∼1690 cm^–^^1^) being particularly strong and broad. This dominant signal obscures the region where urea absorption (∼1650 cm^–^^1^)^74^ would typically appear, making detection via FTIR inconclusive. To complement this analysis, ^1^H NMR spectroscopy was performed on swollen samples (Figure S51) to provide an alternate method for assessing urea content. Neither **PHU**_**3**_ nor **PHU**_**3**_ **-V** exhibited urea signals prior to vitrimerization and processing, respectively (Figures S12 and S51). However, after extended cryogenic grinding, **PHU**_**3**_ **-V** was found to contain 3.3 mol % urea (Figure S51). This content increased only slightly to 3.8 mol % after two mechanical recycling cycles, suggesting that the primary mechanism of recyclability is the dynamic exchange of dioxazaborocane covalent bonds. Apparently, the presence of free hydroxyl groups is necessary for the transcarbamoylation reaction to occur in PHUs. In **PHU_3_-V,** nearly all hydroxyl groups are involved in the formation of dioxazaborocane moieties, and upon heating to 170 °C, only dynamic exchange between the dioxazaborocane units takes place without affecting the carbamate bonds.

### Chemical Recycling of PHU-Vitrimers

Before the chemical recyclability of **PHU**_**3**_ **-V**, its solvent resistance and swelling behavior were examined. Samples were immersed in 6 different solvents for 30 days at 22 °C: water, methanol, cyclohexane, toluene, dimethyl sulfoxide (DMSO), and dichloromethane (Figure 7B, Table S3). All samples remained intact, showing no visible signs of degradation (Figure 7B). It was observed that vitrimer samples swelled in polar solvents (Table S3), with the most swelling in methanol (+86 wt %) and a moderate swelling in DMSO (+67 wt %). In contrast, nonpolar solvents did not penetrate the network, as evidenced by the lower extent of swelling of +2 wt % for cyclohexane and +7 wt % for toluene. The gel content values, measured after drying the samples, were above 97% for all solvents except methanol (85%) and water (90%), which is explained by the fact that both can react with dioxazaborocane moieties and extract 1,4-phenylenediboronic acid.

Several methods for the chemical recycling of dioxazaborocane vitrimers have been reported in the literature. These methods used THF/pinacol mixture or *n*-butylphenylboronic ester^48^ and triethanolamine/DMF,^44^ relying on dioxazaborocane associative transesterification to break down the dynamic moieties into smaller molecules or to lose chain ends. Additionally, it has been reported that B–N coordinated boronic esters are pH-responsive, with the B–N bond dissociating when the pH is either lower than the p*K*_a_ of the acid or higher than the p*K*_a_ of the amine.^75^ Building on this knowledge and the results of solvent resistance tests, we proposed a new approach for recycling of dioxazaborocane **PHU**_**3**_ **-V** vitrimers using ethanol solutions with various acid and base catalysts (Table S4). Ethanol was chosen as it is a nontoxic and widely available alcohol. Most systems were able to break down the vitrimer into small particles at 70 °C within 12 h; however, only the use of 0.1 M NaOH_aq_/ethanol resulted in complete dissolution of the network (Figure 7C). After dissolution, the ethanol was evaporated and the recovered **PHU**_**3**_ was collected, purified, and analyzed (see SI).

It was previously reported that the combination of ethanol and 30 wt % NaOH at 90 °C can lead to the hydrolysis of carbamate moieties in polymers.^76^ However, under the much milder conditions of this study, using a more diluted base and lower temperature, no polymer degradation was observed. According to ^1^H NMR analysis, the structure of recycled **PHU**_**3**_ was identical to that of pristine **PHU**_**3**_ (Figure 7E). Furthermore, the molecular weight of the recycled **PHU**_**3**_ (15 300 g/mol), as measured by GPC, was very close to that of the original sample (16,900 g/mol) (Figure 7E). The only observed change was an increase in the *M*_w_/*M*_n_ ratio from 2.5 to 4.8 after recycling (Figure 7E, Figure S53), which may indicate some branching due to incomplete removal of the cross-linker. The successful recovery of **PHU**_**3**_ was further supported by recent study of Chen et al.,^77^ which reported almost no polymer degradation when using EtOH/*t*-BuOK. In contrast, substituting ethanol with methanol led to complete depolymerization.

The recycled **PHU**_**3**_ was subsequently used to resynthesize **PHU**_**3**_ **-V** following the same procedure as previously described. Both the chemically recycled **PHU**_**3**_ and **PHU**_**3**_ **-V** samples were processed into bars (approximate length × width × thickness = 20 × 5 × 1 mm) using the methods detailed earlier and analyzed using DMTA (Figure 7D, Figure S52). The recycled **PHU**_**3**_ exhibited *T*_α_ and storage modulus values nearly identical to those of the original **PHU**_**3**_ sample (Figure 7D, left). However, recycled **PHU**_**3**_ **-V** showed a higher room temperature storage modulus (1670 vs 1060 MPa) and a slightly lower *T*_α_ (156 vs 167 °C) compared to the pristine **PHU**_**3**_ **-V** sample (Figure 7D, right). This is consistent with a reduction in the efficiency of dioxazaborocane cross-linking, with a lower cross-link density reducing *T*_α_ while in parallel the unreacted hydroxyl groups increase the cohesive energy density and therefore the glassy modulus. Nevertheless, these results clearly demonstrate successful chemical recycling and highlight the potential for further process optimization.

### Foaming of PHU Vitrimers

To further demonstrate the potential utility of the PHU vitrimers described in this study, a lightweight foam from **PHU**_**3**_ **-V** (Figure 8C) was prepared following a process previously developed for thermoplastic PHUs.^54^ The vitrimer sample was swelled in ethanol, which served three purposes: it acted as a plasticizer to reduce the polymer’s *T*_α_, as a reagent potentially capable of temporarily decreasing the cross-link density of the vitrimer (via bond exchange with the dioxazaborocane moieties; see Figure 7C) during the foaming process, and as a physical blowing agent. The swollen **PHU**_**3**_ **-V** was foamed using a vacuum compression molding device at 100 °C and a pressure below 0.5 mbar for 4 h followed by further drying at 120 °C for 8 h. Such prolonged heating was necessary to remove all traces of ethanol from the system and to restore the highly cross-linked nature of the network. Prolonged heating was necessary to remove all residual ethanol from the system and restore the highly cross-linked structure of the network (the presence of ethanol in significant amounts could potentially induce limited bond exchange between the dioxazaborocane moieties and ethanol, thereby slightly reducing the cross-link density; once the ethanol is fully evaporated, the dioxazaborocane moieties can reform, leading to the recovery of the original cross-link density.) The rapid evaporation of the majority of ethanol under these conditions, combined with fast bond exchange within the system, allowed for the production of foam with a density of ≈ 0.07 g/cm^3^. While the optimization of the foaming process and the study of the structure–processing–properties relations of the resulting foams is beyond the scope of the current report, this preliminary result provides a clear demonstration of the wase with which these materials may be foamed. The ability to accomplish this with ethanol is significant as well, given a number of clear advantages vs industrially utilized physical blowing agents: (1) no ozone depletion potential (as compared to HCFCs); (2) lower flammability (as compared to volatile hydrocarbons); (3) biobased sourcing.

## Conclusions

PHUs are often derived from renewable resources and are consistently presented as safer and more sustainable than traditional isocyanate-based polyurethanes. However, they remain uncompetitive from a performance standpoint due to a combination of low molecular weights, insufficient viscoelastic/mechanical properties, and significant moisture absorption. The development of reprocessable, recyclable PHU vitrimers with excellent mechanical performance and hydrolytic stability addresses these shortcomings and provides a means of realizing the promise of PHUs in practice.

In this work, we suggest the use of specifically designed cyclic carbonate monomers to enable the formation of PHUs with proximal hydroxyl groups to allow vitrimer formation via cross-linking with 1,4-phenylenediboronic acid. Using this approach, three PHU vitrimers were synthesized, incorporating nonstabilized dioxaborolane (**PHU**_**1**_ **-V**), nonstabilized dioxazaborocane (**PHU**_**2**_ **-V**) and nitrogen-stabilized (**PHU**_**3**_ **-V**) dioxazaborocane moieties. For the first time, using solid-state natural abundance ^15^N CP-MAS NMR spectroscopy, it was demonstrated that aliphatic nitrogen atoms in **PHU**_**3**_ **-V** can form dative N→B bonds, leading to enhancements in stability and performance. In contrast, the analogous vitrimer **PHU**_**2**_ **-V** containing aromatic nitrogen atoms was found to lack dative N→B bonds, yielding larger 9- or 10-membered rings and displaying reduced stability, even compared to the dioxaborolane-based **PHU**_**1**_ **-V** network. The advantages of thermoplastic PHU modification via formation of boron-containing cross-links can be summarized as follows: (1) an increase in glass transition temperature (by ∼60–105 °C, to ∼110–170 °C, see Figure 2A); (2) an enhancement in tensile strength (by ∼65%, to ∼55 MPa, see Figure 2B) and elongation at break (by ∼120%, to ∼6%, see Figure 2B); (3) a reduction in the sensitivity of mechanical properties to humidity and moisture absorption (∼70% retention of storage modulus when moving from 45 to 85% RH); (4) significantly improved tensile toughness (∼11 times greater); (5) mechanical recyclability over multiple recycles without significant changes in appearance or loss of properties; (6) short reworking times (10 min) due to rapid bond exchange; (7) chemical recyclability using an ethanol/NaOH_aq._ mixture, including network decomposition, recovery of the base PHU polymer, and regeneration of the associated vitrimer without loss of properties; (8) foamability using ethanol as a physical blowing agent.

In summary, the enhanced thermal and hydrolytic stability and improved mechanical properties of PHUs achieved using nitrogen-stabilized dioxazaborocane moieties represent an attractive means of PHU vitrimerization. The formation of neutral complexes containing dative bonds that provide a stabilizing effect under use conditions/in the presence of moisture and an activating effect at elevated temperatures when bond exchange is desired (i.e., for recycling and/or foaming) represents a new strategy with relevance to vitrimer design more broadly. The resulting ability to control properties, realize mechanical and chemical reprocessability, and enhance durability highlights the potential of this approach in terms of both fundamental science and practical application.

## Supplementary Material



## Notes

Materials and methods, synthesis and characterization of cyclic carbonates, PHU polymers and PHU vitrimers, solid state NMR spectra, DSC plots of PHU polymers and vitrimers, water absorption plots, SEM images of milled vitrimer samples, DMTA plots, swelling and gel content data for **PHU_3_-V**, DMTA and stress–strain plots for mechanically recycled **PHU_3_-V** specimens, chemical recycling procedures and GPC and DMTA plots for chemically recycled **PHU_3_-V** ^78–82^ (PDF)
