# Supplementary material for: Enhancing Polyhydroxyurethane Properties via the Formation of Dioxaborolane- and Dioxazaborocane Vitrimers
Source: Chem Mater. 2025 Sep 11;37(18):7049–63. doi: 10.1021/acs.chemmater.5c01048 (PMC12461831; doi:10.1021/acs.chemmater.5c01048)
Supplement: Supplementary file 1 [file cm5c01048_si_001.pdf]

Supporting information for

Enhancing polyhydroxyurethane properties via the formation of dioxaborolane- and dioxazaborocane vitrimers

Sergei V. Zubkevich*,†, Arpan Datta Sarma†, Oleg N. Antzutkin‡, Reiner Dieden†, Vincent Berthe†, Stephan Westermann†, Alexander S. Shaplov*,† and Daniel F. Schmidt*,†

† Luxembourg Institute of Science and Technology (LIST), 5 Avenue des Hauts-Fourneaux, L-4362 Esch-sur-Alzette, Luxembourg

‡ Chemistry of Interfaces, Luleå University of Technology, SE-97187 Luleå, Sweden

Table of Contents

I. Materials 2

II. Methods 2

III. Synthesis and characterization of cyclic carbonates. 6

III.1. 4,4'-((phenylazanediyl)bis(methylene))bis(1,3-dioxolan-2-one) (**M_2**). 6

III.2. 4,4'-((cyclohexylazanediyl)bis(methylene))bis(1,3-dioxolan-2-one) (**M_3**). 8

IV. Synthesis and characterization of PHU polymers. 11

IV.1. General procedure for synthesis. 11

IV.2. PHU based on **M_2** and HMDA (**PHU_2**). 11

IV.3. PHU based on **M_3** and HMDA (**PHU_3**). 14

IV.4. Calculation of hydroxyl group content 16

V. Synthesis and characterization of PHU vitrimers. 17

V.1. General synthetic procedure. 17

V.2. **PHU_1-V**. 17

V.3. **PHU_2-V**. 17

V.4. **PHU_3-V**. 18

VI. Solid state NMR 19

VII. DSC plots of PHU polymers and vitrimers. 30

VIII. Water absorption plots. 33

IX. SEM of milled **PHU_1-V**, **PHU_2-V** and **PHU_3-V**. 34

X. DMTA plots 37

XI. Solvent resistance and swelling. 38

XII. Mechanical recycling. 39

XIII. Chemical recycling. 43

XIV. Evaluation of PHU_3-V hydrolytic stability in boiling water. 46

XV. Comparison of thermos-mechanical properties and reprocessability of PHU vitrimers 48

I. Materials

Reagents: *meso*-Erythritol (>99%, TCI Chemicals), diphenyl carbonate (99%, Sigma-Aldrich), cyclohexylamine (>99%, TCI Chemicals), glycidol (96%, Sigma-Aldrich), dimethyl carbonate (DMC, >98%, TCI Chemicals), 1,5,7-triazabicyclo[4.4.0]dec-5-ene (TBD) (97%, BLD Pharm), 1,4-phenylenediboronic acid (contains varying amounts of anhydride, TCI Chemicals), 2,3-butanediol (98%, Sigma-Aldrich), phosphorus pentoxide (immobilized on silica, with indicator, Sicapent®, Sigma-Aldrich), lithium bis(trifluoromethanesulfonyl)imide (LiTFSI, 99.9%, Solvionic), silica gel (high-purity grade, average pore size 60 Å (52-73 Å), 70-230 mesh, 63-200 µm, for column chromatography, Sigma-Aldrich), sodium hydroxide (extra pure, pellets, Acros Organics), potassium hydroxide (>85%, pellets, Carl Roth), ammonium hydroxide (25% solution in water, Acros Organics) were used as received.

Hexamethylene diamine (HMDA, 98%, Sigma-Aldrich) and aniline (ACS reagent, ≥99.5%, Sigma-Aldrich) were purified by vacuum distillation over NaOH and were kept under argon in the dark place prior to use. Anhydrous magnesium sulfate was prepared in-house from $MgSO_4 \cdot 7H_2O$ ($MgSO_4$, >99%, Aldrich) by heating a saturated aqueous solution of magnesium sulfate in a stainless-steel pan (~500 mL) using a hot plate (Severin 1500 W) set to maximum heating. Following evaporation of all liquid water, heating was continued for another ~6-8 h (T > 200 °C) to obtain an anhydrous cake. The cake was allowed to cool to ~70 °C, broken into ~1 cm sized pieces, then stored in a sealed glass jar prior to use. Calcium sulfate (Drierite™, 8 mesh, with indicator, Sigma-Aldrich) was dried at 230 °C for 2 hours in the oven prior to use. Erythritol dicarbonate (**M_1**) and **PHU_1** polymer from the reaction of M_1 and HMDA were synthesized in accordance with published procedures.[1]

Solvents: Ethanol (>95%, Fisher Scientific (Acros Organics)), methanol (>99.5%, Fisher Scientific (Acros Organics)), tetrahydrofuran (THF, 99.6%, Fisher Scientific (Acros Organics)), dichloromethane (>99.5%, Fisher Scientific (Acros Organics)), toluene (99+%, SLR, Extra Pure, Fisher Scientific (Acros Organics)), cyclohexane (SLR, Extra Pure, Fisher Scientific(Acros Organics)) diethyl ether (99+%, Fisher Scientific (Acros Organics)), *n*-hexane (SLR, Extra Pure, Fisher Scientific (Acros Organics)), ethyl acetate (>99.5%, Fisher Chemical), N,N-Dimethylformamide (DMF, ACS reagent, ≥99.8%, Sigma-Aldrich), dimethylsulfoxide (DMSO, ≥99.5%, for synthesis, Carl Roth), 1-methyl-2-pyrrolidone (NMP, 99.5%, Extra Dry, AcroSeal, Fisher Scientific (Acros Organics)) were used as received. Ultrapure deionized water was obtained using Sartorius Arium® Comfort smart station.

NMR solvents and standards: Dimethyl sulfoxide-d_6 (DMSO-d_6, 99.9 at% D, Sigma-Aldrich) was stored over 3 Å molecular sieves.

II. Methods

II.1. Solution nuclear magnetic resonance (NMR) spectra were recorded on Avance III HD 600MHz (Bruker) spectrometer (^1H NMR at 600 MHz, ^{13}C NMR at 151 MHz and ^{19}F at 565 MHz) at 25°C (unless stated otherwise) in the indicated deuterated solvent and are listed in ppm. The signals corresponding to the residual protons and carbons of the deuterated solvent were used as an internal standard for ^1H and ^{13}C NMR, respectively. Traces of common non-deuterated solvents and impurities were identified according to *Fulmer et al.*[2]

[1] Zubkevich, S. V; Makarov, M.; Dieden, R.; Puchot, L.; Berthé, V.; Westermann, S.; Shaplov, A. S.; Schmidt, D. F. Unique Method for Facile Postsynthetic Modification of Nonisocyanate Polyurethanes. *Macromolecules* **2024**, *57* (5), 2385–2393. https://doi.org/10.1021/acs.macromol.3c02232

[2] Fulmer, G. R.; Miller, A. J. M.; Sherden, N. H.; Gottlieb, H. E.; Nudelman, A.; Stoltz, B. M.; Bercaw, J. E.; Goldberg, K. I. *Organometallics* **2010**, *29* (9), 2176–2179. https://doi.org/10.1021/om100106e

II.2. Solid-state (^{15}N, ^{13}C and ^{11}B) nuclear magnetic resonance (ssNMR) spectra were obtained under magic-angle-spinning (MAS) conditions on a Bruker Ascend Aeon WB 400 (Bruker BioSpin AG, Fällanden, Switzerland) NMR spectrometer with a superconducting "zero-helium boil-off" Aeon magnet (B_0 = 9.4 T). A double-channel high-power "Varian" 4 mm-MAS probe was tuned on ^{1}H (400.21 MHz) in one (H) channel and either on ^{15}N (40.56 MHz), or ^{13}C (100.64 MHz), or ^{11}B (128.40 MHz) NMR resonance frequencies in the other (X) channel. Ca. 40 - 70 mg of polymers (packed in sealed plastic bags immediately after the synthesis and opened and crushed in a mortar to ca. 1 mm size at RH < 20% just prior to analysis) or a fine white powder (for solid reagent(s)) were packed in a 4 mm thin-wall zirconia rotor with tightly closed (with Teflon) caps and spun at 5 kHz (for ^{15}N), 5 – 5.6 kHz (for ^{13}C) and from 3 to 10 kHz (for ^{11}B). ^{11}B MAS NMR spectra were recorded using the direct-excitation single-pulse experiment without proton decoupling (22.5°-pulse, 1.2 μs pulse-width) with a recycling delay of 3 s and signal transient accumulations from 1336 to 5336 for different samples of varied density and, therefore, different weights. ^{15}N and ^{13}C CP-MAS NMR spectra were obtained under MAS conditions with cross-polarization (CP) from protons using ramping of the rf-field in the X-channel from 70 to 100 % of the maximum amplitude and 1.5 or 2 ms contact times for ^{1}H-^{15}N or ^{1}H-^{13}C, respectively, using a 76 kHz "Spinal-64" sequence[3] for proton decoupling with a train of rf-pulses of different phases in the ^{1}H channel. Here, the excitation 90°(^{1}H)-pulse of 3.3 μs pulse-width was followed by the CW contact time pulse and then ^{1}H-decoupling during the signal acquisition in the X-channel, all at the same rf-power in the ^{1}H-channel. A recycling delay of 3 s (10 s in ^{13}C NMR experiments for reagents with slowly relaxing aromatic carbons) and signal transient accumulations from 1464 to 20000 (for ^{13}C) or from 26778 to 50000 (for ^{15}N) were needed to reach signal-to-noise levels better than ten-to-one and for justified quantification of relative integral intensities of different N-sites in ^{15}N CP-MAS NMR spectra of vitrimers. ^{11}B NMR spectra were externally referenced using a liquid sample of $Et_2O{\cdot}BF_3$ (0 ppm), which was placed in a small capillary (1 mm in diameter) and inserted into an empty 4 mm rotor to minimize the differences in the magnetic susceptibilities between solid samples and the liquid reference. For ^{13}C NMR spectra, solid adamantine (38.48 ppm from TMS, $\delta(^{13}C)$ = 0 ppm) was used as an external reference. ^{15}N NMR spectra were also externally referenced using a polycrystalline sample of ^{15}N-enriched (98%) $^{15}NH_4Cl$ (0 ppm). The latter sample was also used for setting of the Hartmann-Hahn condition for the rf-power in the X-channel during the contact time, adjusted for the maximum signal intensity in the ^{1}H-^{15}N cross-polarization NMR experiment. All measurements were performed at 293 K. NMR spectra were processed using the Bruker Topspin 3.5 software.

II.3. Size exclusion chromatography (SEC) / gel permeation chromatography (GPC) was used to determine number-average molecular weights ($M_{n(GPC)}$) and M_w/M_n ratios. Analyses were performed on a 1200 Infinity gel permeation chromatograph (Agilent Technologies) equipped with PLgel 5μm MIXED-D column (Agilent Technologies), PLgel 5μm (Agilent Technologies) pre-column and an integrated refractive index detector. The system was operated at 50 °C and 1.0 mL/min flow using 0.1 M $Li(CF_3SO_2)_2N$ (LiTFSI) solution in DMF as an eluent. Poly(methyl methacrylate) standards (EasiVial PM, Agilent Technologies, M_p = 550 - 1558×10^3) were used for calibration.

II.4. Thermal gravimetric analysis (TGA) was carried out in air on a TGA 2 STARe System (Mettler Toledo) applying a heating rate of 5 °C/min. The onset weight loss temperature (T_{onset}) was determined as the point in the TGA curve at which a significant deviation (0.5-1.0%) from the horizontal was observed. The resulting temperature was then rounded to the nearest 5 °C.

II.5. Differential Scanning Calorimetry (DSC) was performed on a DSC 300 Caliris Select differential calorimeter (Netzsch). At least two heat/cool cycles were carried out under N_2 at 5 °C/min for each

[3] Fung, B. M.; Khitrin, A. K.; Ermolaev, K. An Improved Broadband Decoupling Sequence for Liquid Crystals and Solids. *J. Magn. Reson.* **2000**, *142*, 97−101.

sample. Glass transition (T_g) and the melting temperatures (T_m) were determined from the second heating curve, while crystallization temperatures (T_{cr}) were identified from the second cooling curve. For simplicity T_m and T_{cr} were taken from the peak maxima.

II.6. Scanning Electron Microscopy (SEM) was performed using a Quanta 200 Field Emission Scanning Electron Microscope (FE-SEM) from Philips-FEI. The microscope was equipped with a Genesis XM 4i Energy Dispersive Spectrometer (from EDAX) system for chemical analysis. The pictures were acquired in "Composition mode" using the backscattered electron detector (BSED) with the following parameters: pressure – 60 Pa, acceleration voltage – 20 kV, spot size parameter – 5, working distance – ≈ 9 mm. Particle sizes were calculated using the Image J software package.

II.7. Thermomechanical analysis, water adsorption and swelling studies

Sample preparation. (A) Thermoplastic samples. Polymer bars (typical length × width × thickness = 20 × 5 × 1.5 mm) were prepared using a MeltPrep (VCM Essentials, MeltPrep GmbH) vacuum compression molding apparatus. Polymer powder (~ 0.15 g) was loaded into a mold and heated for 10 minutes at 120 °C for amorphous samples or at 140 °C for crystalline polymers under a vacuum of ~ 0.1 mbar. Afterwards the mold was allowed to cool down to room temperature, opened, and the polymer bar was released. Then samples were conditioned for at least 24 h at 21 ± 2°C in the opened vials inside a desiccator filled with anhydrous $CaSO_4$ (Drierite™).
(B) Vitrimer samples. Polymer bars (typical length × width × thickness = 20 × 5 × 1.5 mm) were prepared using MeltPrep (MeltPrep GmbH) molds and a Carver manual press (model 3851). Vitrimer powder (~ 0.17 g) was molded for 10 minutes at 170 °C with a force of 0.25 tons. Afterwards the mold was allowed to cool down to room temperature without pressure, opened, and the vitrimer bar was released. The samples were then conditioned for at least 24 h at 21 ± 2°C in the opened vials inside a desiccator filled with anhydrous $CaSO_4$ (Drierite™).

Dynamic Mechanical Thermal Analysis (DMTA) was carried out on polymer bars (typical length × width × thickness = 20 × 5 × 1.5 mm) using a GABO Eplexor 500N instrument (Netzsch-Gerätebau GmbH) operating in tension mode (dynamic strain: 0.25 %, pretension (preload force): 0.8 N, static strain of 1%). Experiments were performed at a frequency of 1 Hz with a heating rate of 3 °C/min in temperature ranges of -50 to 120 °C for thermoplastic samples and -50 to 190 °C for vitrimer samples. The apparatus provided the storage and loss moduli (E' and E''), with the damping parameter or loss factor (tan δ) defined as the ratio tan $\delta = E''/E'$. While it is a common practice to determine T_α based on the tan δ peak, here T_α is derived from the loss modulus peak, in line with multiple reports specifically recommending this as the more appropriate approach for properly defining the main relaxation temperature.[4]

Rheology

Frequency sweep measurements were performed in torsion mode with bar specimens using an Anton Paar Physica MCR 302 rheometer equipped with a CTD 180 temperature control device and an MHG100 humidity controller. Polymer samples in a form of bars (typical length × width × thickness = 20 × 5 × 1.5 mm) were stored for 72 h at the specified relative humidity level (45%, 65% or 85%) before measurement and then loaded directly into the rheometer. The samples were tested in frequency sweep mode from 0.1 to 100 Hz with an imposed shear strain of 0.1%, ensuring that both G' and G'' were obtained in the linear viscoelastic regime. All measurements were carried out at 25 °C and constant humidity (45%, 65% or 85% RH) as maintained by the MHG100 humidity controller.

Relaxation experiments were performed in torsion mode with bar specimens using an Anton Paar Physica MCR 302 rheometer equipped with a CTD 450 cell and EVU 20 temperature controller.

[4] (a) Rieger, J. *Polym. Test.* **2001**, *20* (2), 199–204. https://doi.org/10.1016/S0142-9418(00)00023-4; (b) Lei, Z.; Xing, W.; Wu, J.; Huang, G.; Wang, X.; Zhao, L. *J. Therm. Anal. Calorim.* **2014**, *116* (1), 447–453. https://doi.org/10.1007/s10973-013-3526-0

Polymer samples in the form of bars (typical length × width × thickness = 20 × 5 × 1.5 mm) were stored for 72 h in a desiccator over P_2O_5 before measurement and then loaded directly into the rheometer. All measurements were carried out in air. The sample was allowed to equilibrate at the appointed temperature for 5 min and then a constant strain (0.1%) was applied. The relaxation time (in s) was defined as the time at which the stress decayed to 37% (1/e) of the value observed at 1 s after the start of the measurement[5].

Water absorption or water uptake was measured for each polymer sample using bars (typical length × width × thickness = 20 × 5 × 1.5 mm) at three humidity levels (45%, 65% and 85% RH) according to ASTM E337-15. Bars were weighed after specified periods of time (24 h, 72 h, 7 days, and 11 days) and water absorption was calculated using the following equation:

$$\text{Water uptake (wt\%)} = \frac{\text{Weight of bar (stored at specified humidity for X h)}}{\text{Weight of bar (right after preparation)}} \times 100\% \qquad \text{(Eq. S1)}$$

Swelling degree and gel content values were measured by submerging a square piece cut from a vitrimer bar in the selected solvent at 25°C in a closed vial. The sample was allowed to swell for 30 days, then removed from the solvent, wiped with a paper towel to remove the excess solvent from the surface, and weighed. The extent of swelling was calculated using the following equation:

$$\text{Swelling (wt\%)} = \frac{\text{Weight of bar (conditioned for 30 days)} - \text{Weight of bar (before conditioning)}}{\text{Weight of bar (before conditioning)}} \times 100\% \qquad \text{(Eq. S2)}$$

The sample was then dried in vacuum (< 0.1 mbar) at 120 °C for 4 hours and at 150 °C for 24 hours and weighed. Gel content values were calculated using the following equation:

$$\text{Gel content (wt\%)} = \frac{\text{Weight of bar (dried in vacuum)}}{\text{Weight of bar (before conditioning)}} \times 100\% \qquad \text{(Eq. S3)}$$

II.8. Tensile testing

Sample preparation. (A) Thermoplastic samples. Polymer dumbbells (ISO 37 type 4) were prepared using a MeltPrep (VCM Essentials, MeltPrep GmbH) vacuum compression molding apparatus. Polymer powder (~ 0.15 g) was loaded into a mold and heated for 10 minutes at 120 °C for amorphous samples or at 140 °C for crystalline polymers under a vacuum of ~ 0.1 mbar. Afterwards, the mold was allowed to cool down to room temperature, opened, and the polymer dumbbell was released. Samples were then conditioned for at least 24 h at 21 ± 2 °C in the opened vials inside a desiccator filled with anhydrous $CaSO_4$ (Drierite™).
(B) Vitrimer samples. Polymer dumbbells (ISO 37 type 4) were prepared using MeltPrep (MeltPrep GmbH) molds and a Carver manual press (model 3851). Vitrimer powder (~ 0.17 g) was molded for 10 minutes at 170 °C with a force of 0.25 tons. Afterwards, the mold was allowed to cool down to room temperature without pressure, opened, and the vitrimer bar was released. The samples were then conditioned for at least 24 h at 21 ± 2 °C in the opened vials inside a desiccator filled with anhydrous $CaSO_4$ (Drierite™).

Tensile strength, elongation at break and toughness determination. The quasi-static mechanical properties of polymers were studied using an Instron 68SC-1 universal testing machine (Illinois Tool Works Inc.) operated in tension mode and equipped with a 1 kN load cell and manual clamps (2716-016). Mechanical tests were performed at room temperature (22 ± 1 °C) and a relative humidity of 50 ± 5 %. The crosshead speed was maintained at 2 mm/min during all experiments. To achieve statistically valid results a set of three specimens was tested for each polymeric material. The tensile modulus (Young's modulus) was measured from stress-strain plots within the Hookean region (0.1 – 0.2 % strain)

[5] Capelot, M.; Unterlass, M. M.; Tournilhac, F.; Leibler, L. Catalytic Control of the Vitrimer Glass Transition. *ACS Macro Lett.* **2012**, *1* (7), 789–792. https://doi.org/10.1021/mz300239f

and was calculated using Bluehill Universal (v4.09) software. The tensile toughness was calculated as an integrated area under the stress strain curve and reported in units of MJ·m^{-3}.

II.9. Mechanical recycling.

PHU$_3$-V bars and dumbbells were broken into chunks and milled at room temperature using a Retsch CryoMill ball milling instrument equipped with a 50 mL stainless steel grinding jar with one 20 mm stainless steel ball. The milling was performed at 30 Hz for 2 minutes at temperature < 0°C. The obtained fine powder was dried at 120°C/0.5-1 mbar in a B-585 oven (Buchi Glass Drying Oven, Buchi) over P_2O_5 for 2 days. The milled samples were stored in the plastic zip bags inside a desiccator filled with anhydrous Ca_2SO_4 (Drierite™). The preparation of new bars and dumbbells was performed according to the methods disclosed above.

III. Synthesis and characterization of cyclic carbonates.

III.1. 4,4'-((phenylazanediyl)bis(methylene))bis(1,3-dioxolan-2-one) (M$_2$).

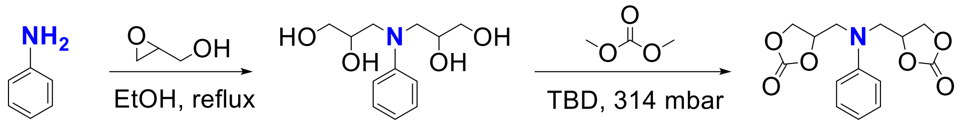

Scheme S1. Synthesis of 4,4'-((phenylazanediyl)bis(methylene))bis(1,3-dioxolan-2-one) (**M$_2$**).

Aniline (30 g, 0.322 mol) and glycidol (2.5 equiv., 59.66 g, 0.805 mol) were dissolved in ethanol (100 mL) in a 250 mL round-bottom flask equipped with a magnetic stirrer and a condenser. The solution was refluxed for 8 hours, then cooled to room temperature. The ethanol was evaporated under reduced pressure, and THF (150 mL) was added to dissolve the oily residue. This solution was precipitated into 700 mL of n-hexane, allowed to settle, and the solvent was carefully decanted, leaving a whitish oil at the bottom of the flask. The oil was dried by stirring at 100°C under dynamic vacuum (<0.5 mbar) overnight, yielding the desired intermediate (77 g, 99 %) as a transparent, colorless oil, which was used in the next step without additional purification.

Next, DMC (250 mL, 8.1 equiv.) and TBD (2.22 g, 0.016 mol, 5 mol%) were added to the intermediate, and the mixture was placed on a rotary evaporator. Following a procedure similar to that reported by *Dannecker & Meier*[6], the mixture was maintained at 60°C and 314 mbar for ~1 hour, until no further methanol distillation was observed. The solution was then allowed to cool to room temperature, prompting the slow crystallization of 4,4′-((phenylazanediyl)bis(methylene))bis(1,3-dioxolan-2-one) (**M$_2$**) as white, needle-shaped crystals. The crystals were collected by filtration, rinsed with a minimal amount of DMC, and dried at 100°C under dynamic vacuum (<0.5 mbar) overnight. Yield: 34.8 g (37%).
Melting point (DSC, 5°C/min) T_m = 188.5°C. Anal. calcd. for $C_{14}H_{15}NO_6$ (293.28): C, 57.34%; H, 5.16%; N, 4.78%. Found: C, 57.21%; H, 5.07%; N, 4.66%.
^{1}H NMR (600 MHz, DMSO-d$_6$) δ 7.19 (tt, J = 7.2, 2.3 Hz, 2H), 6.88 (ddt, J = 19.3, 7.2, 1.1 Hz, 2H), 6.70 (tq, J = 7.3, 0.9 Hz, 1H), 4.99 (dddd, J = 17.1, 8.5, 7.0, 3.3 Hz, 2H), 4.58 (dt, J = 11.0, 8.3 Hz, 2H), 4.17 (ddd, J = 8.4, 7.0, 3.3 Hz, 2H), 3.90 – 3.79 (m, 2H).
^{13}C NMR (151 MHz, DMSO-d$_6$) δ 154.88, 146.73, 129.34, 117.01, 112.88, 112.57, 75.12, 67.30, 53.40.

[6] Dannecker, P.; Meier, M. A. R. Facile and Sustainable Synthesis of Erythritol Bis (Carbonate), a Valuable Monomer for Non-Isocyanate Polyurethanes (NIPUs). *Sci. Rep.* **2019**, 9, 9858. https://doi.org/10.1038/s41598-019-46314-5

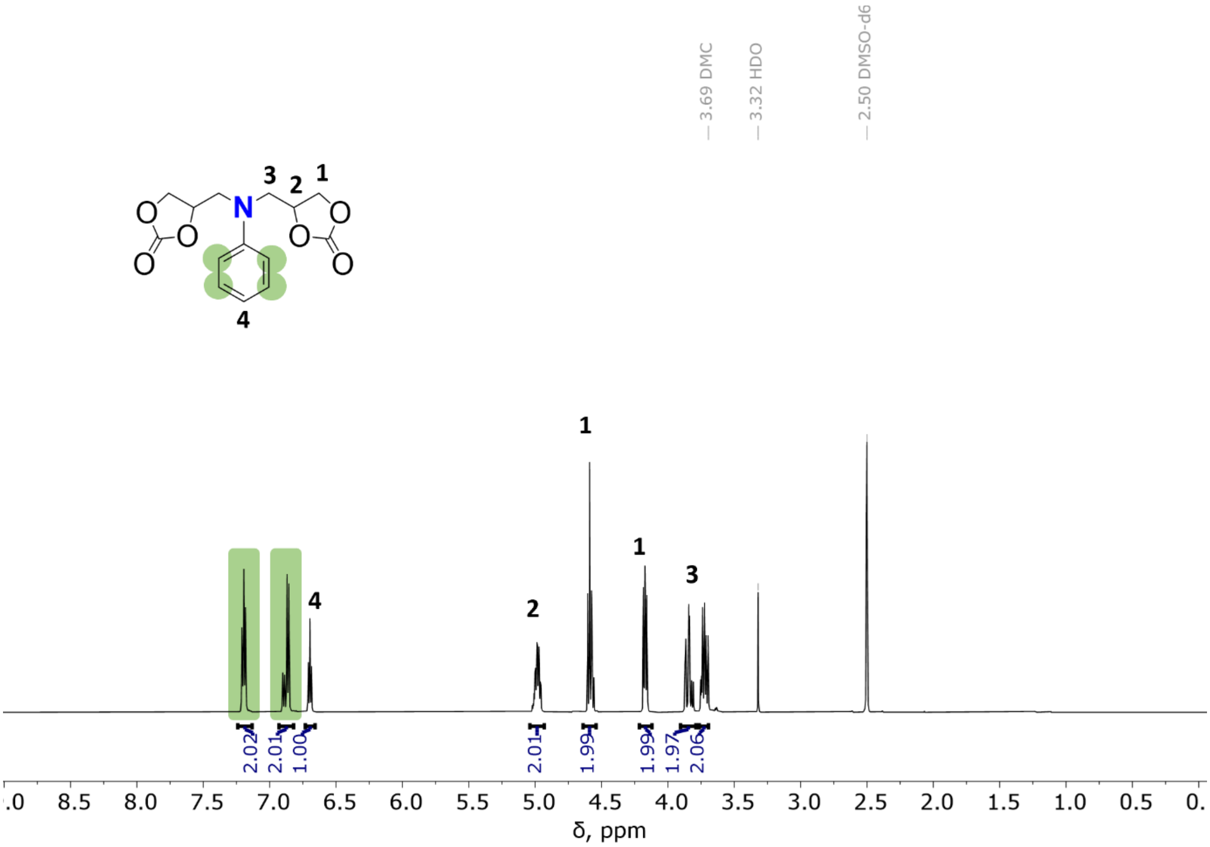

Figure S1. ^{1}H NMR spectrum of $\mathbf{M_2}$ in DMSO-d_6.

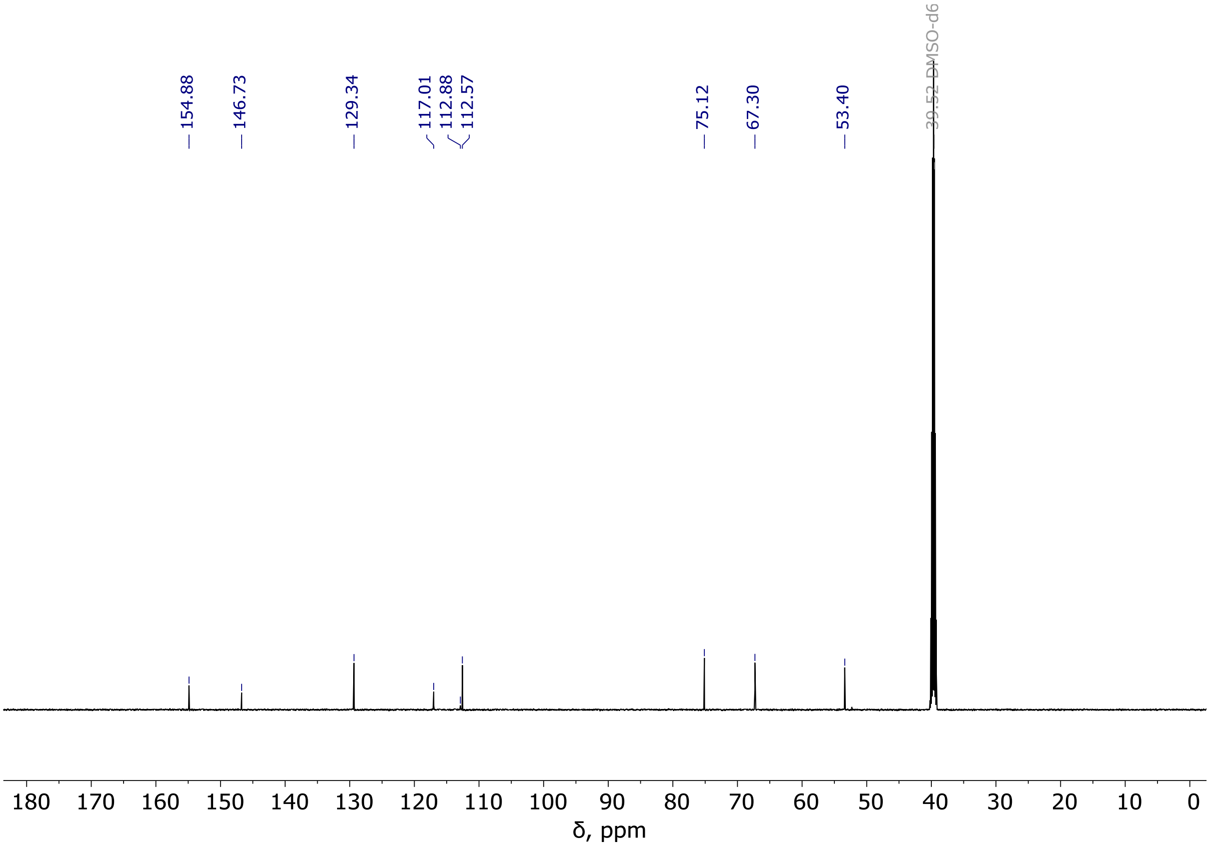

Figure S2. ^{13}C NMR spectrum of $\mathbf{M_2}$ in DMSO-d_6.

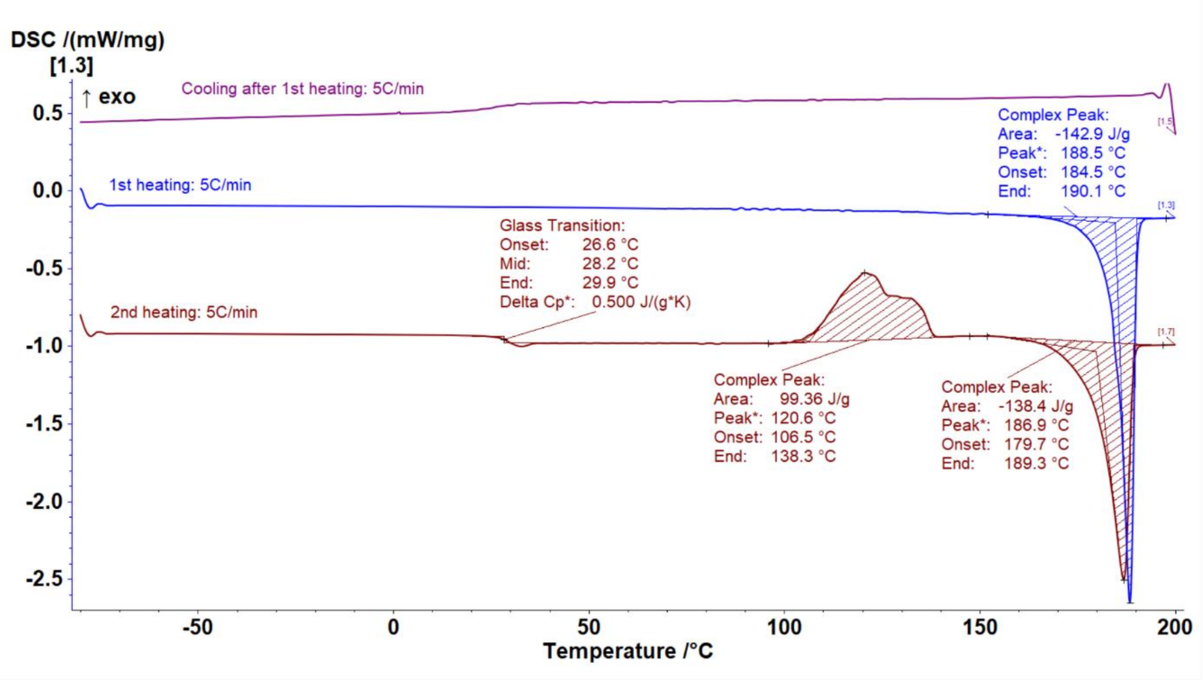

Figure S3. DSC plots of **M₂**.

III.2. 4,4'-((cyclohexylazanediyl)bis(methylene))bis(1,3-dioxolan-2-one) (**M₃**).

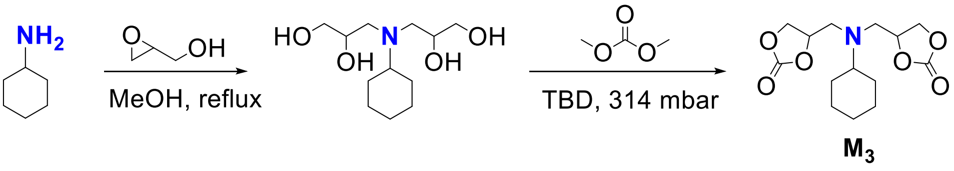

Scheme S2. Synthesis of 4,4'-((cyclohexylazanediyl)bis(methylene))bis(1,3-dioxolan-2-one) (**M₃**).

Cyclohexylamine (15 g, 0.151 mol) and glycidol (2.5 equiv., 28.01 g, 0.378 mol) were dissolved in methanol (60 mL) in a 250 mL round-bottom flask equipped with a magnetic stirrer and a condenser. The solution was refluxed for 8 hours, then allowed to cool to room temperature. Most of the methanol was subsequently removed under reduced pressure, and THF (50 mL) was added to dissolve the resulting oily mixture. This solution was then precipitated into 300 mL of n-hexane. After the mixture was left to settle, the supernatant was carefully decanted, leaving a whitish oil at the bottom of the flask. The oil was dried under dynamic vacuum (<0.5 mbar) with stirring at 60°C for 2 hours and then at 100°C overnight, yielding the desired intermediate (37 g, 99 %) as a transparent, colorless oil, which was used in the next step without further purification.

Next, DMC (216 mL, 15 equiv.) and TBD (1.04 g, 0.0075 mol, 5 mol%) were added to the intermediate, and the mixture was placed on a rotary evaporator. The synthesis was carried out as described for **M₂**. After methanol distillation ceased, the vacuum was adjusted to remove any residual DMC. The crude mixture was then redissolved in a minimal volume of dichloromethane and filtered through a pad of silica, which was subsequently washed with approximately 350 mL of dichloromethane to ensure complete recovery of the target compound. The dichloromethane solution was concentrated to about 50 mL, and the target compound was recrystallized from a dichloromethane/diethyl ether mixture in the freezer. The resulting white powder was collected by filtration, rinsed with diethyl ether, and dried under dynamic vacuum (<0.5 mbar) at 60°C overnight to afford pure 4,4′-((cyclohexylazanediyl)bis(methylene))bis(1,3-dioxolan-2-one) (**M₃**). Yield: 18.24 g (41 %).

Melting point (DSC, 5°C/min) T_m = 86.1°C. Anal. calcd. for $C_{14}H_{21}NO_6$ (299.32): C, 56.18%; H, 7.07%; N, 4.68%. Found: C, 56.00%; H, 7.03%; N, 4.60%.

^{1}H NMR (600 MHz, DMSO-d$_6$) δ 4.78 (dq, J = 7.8, 6.1 Hz, 2H), 4.52 (td, J = 8.2, 2.8 Hz, 2H), 4.14 (ddd, J = 8.4, 6.9, 1.6 Hz, 2H), 2.88 – 2.75 (m, 4H), 2.46 – 2.39 (m, 1H), 1.78 – 1.61 (m, 4H), 1.56 (d, J = 12.7 Hz, 1H), 1.18 (q, J = 11.6 Hz, 4H), 1.03 (d, J = 12.2 Hz, 1H).

^{13}C NMR (151 MHz, DMSO-d$_6$) δ 154.85, 76.17, 67.55, 61.05, 52.83, 29.03, 28.27, 27.54, 25.64, 25.59, 25.55, 25.53.

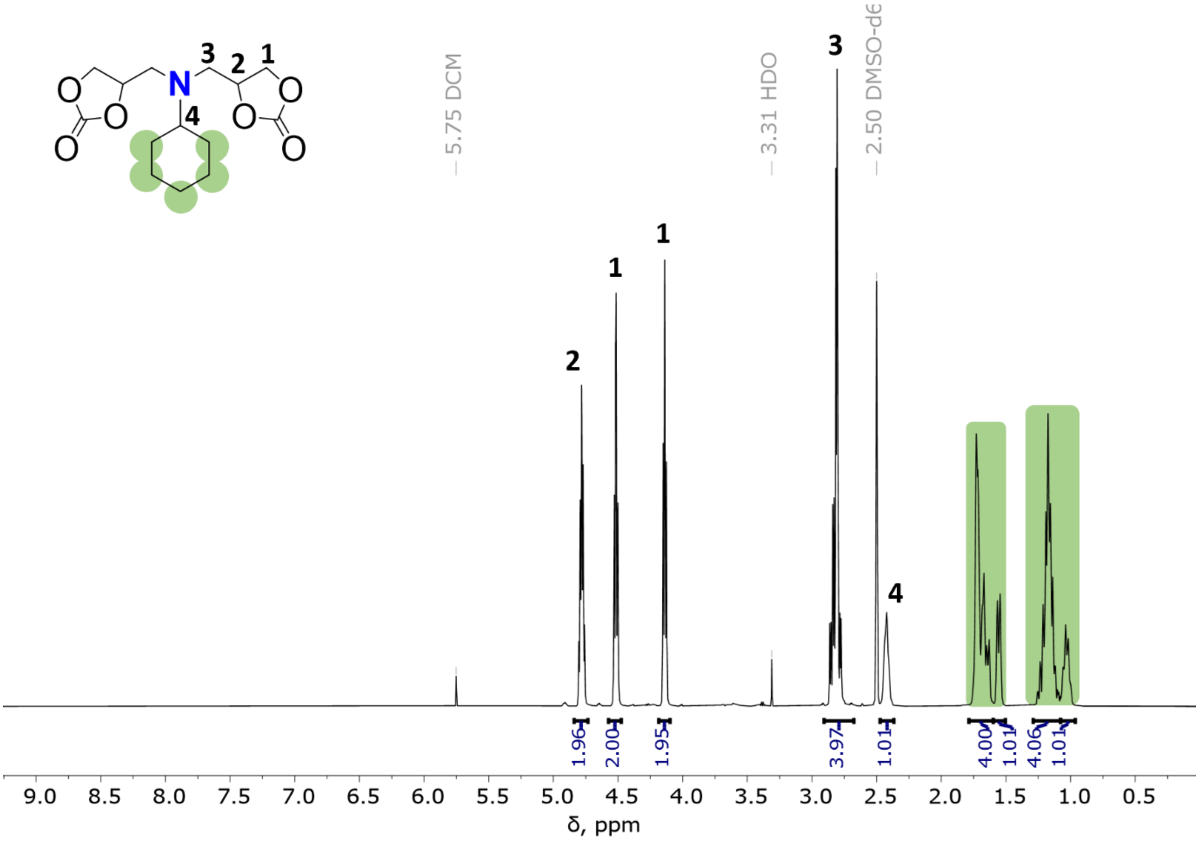

Figure S4. ^{1}H NMR spectrum of **M_3** in DMSO-d$_6$.

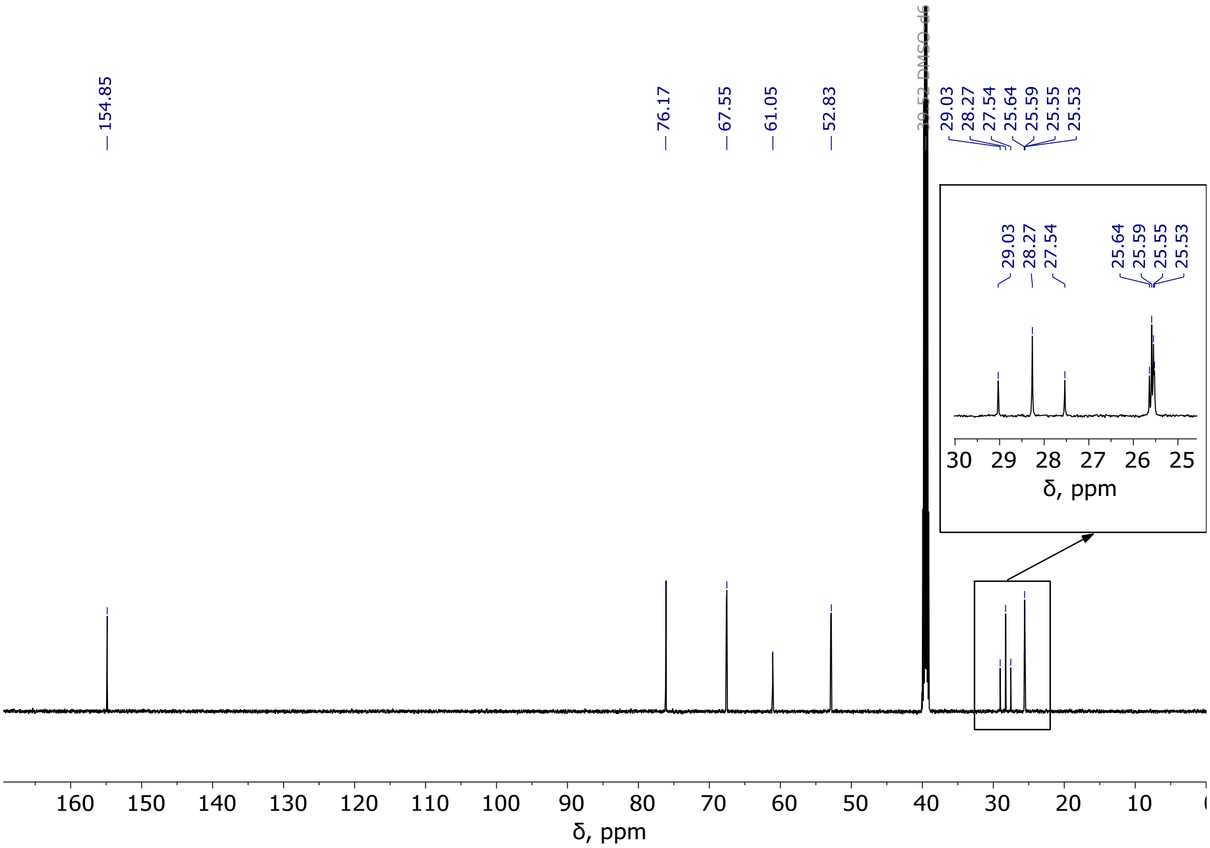

Figure S5. ^{13}C NMR spectrum of $\mathbf{M_3}$ in DMSO-d_6.

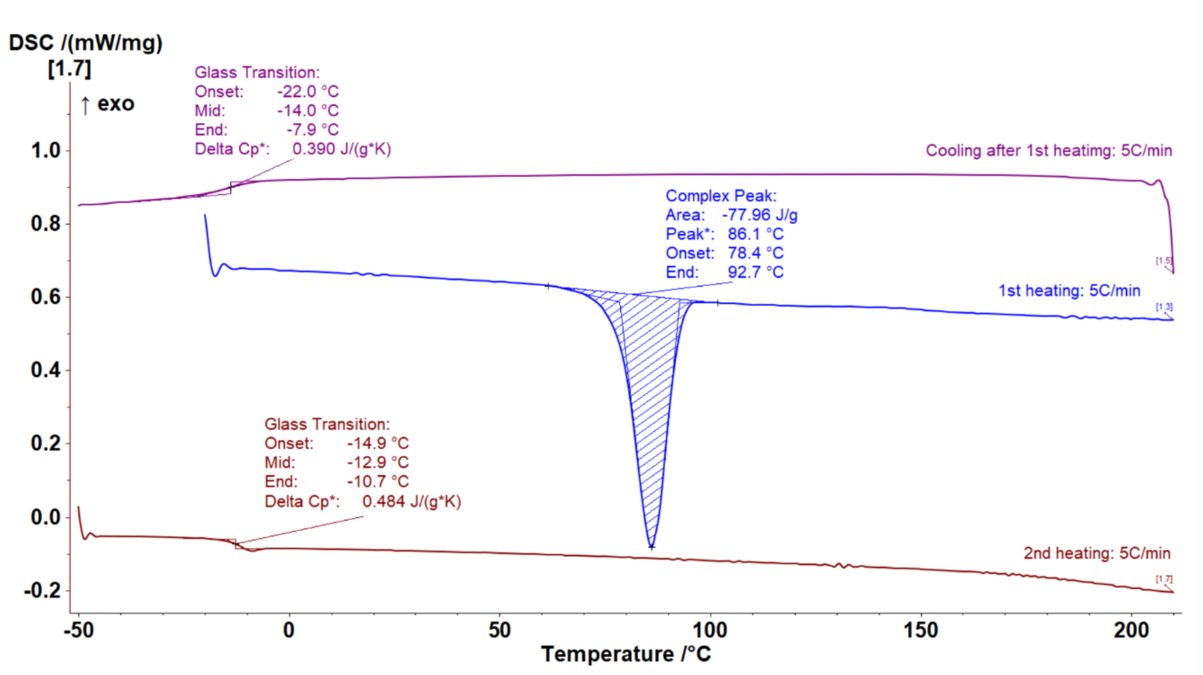

Figure S6. DSC plots of $\mathbf{M_3}$.

IV. Synthesis and characterization of PHU polymers.

IV.1. General procedure for synthesis.

The procedure used to synthesize PHU polymers was adapted from our previous studies.[7] A microcompounder (Figure S7) was preheated to 100°C and purged with argon for 5 minutes. The screws were then set to rotate at 120 rpm. The appropriate cyclic dicarbonate and diamine (in a 1:1 molar ratio) were quickly mixed with N-methyl-2-pyrrolidone (NMP, 30 wt%) to form a paste, which was injected into the microcompounder through a funnel. The funnel was then removed, and the injection inlet was sealed with a stainless-steel cap. The reaction proceeded at 100°C for 2.5 hours under an inert atmosphere, maintaining the screw speed at 120 rpm.

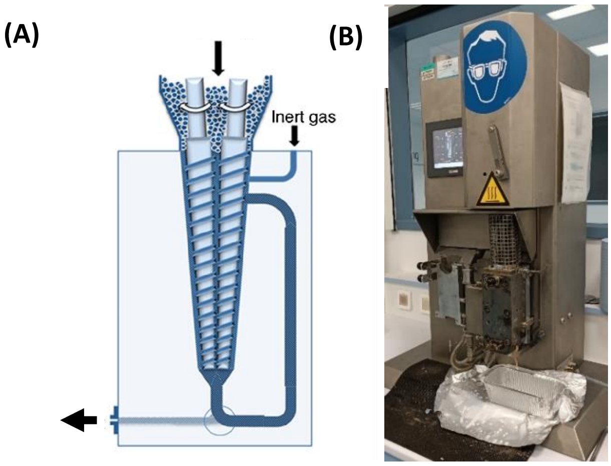

Figure S7. Schematic representation (**A**) and photo (**B**) of twin-screw microcompounder Xplore MC15, that was used for reactive extrusion synthesis of PHU_1, PHU_2 and PHU_3 polymers.

After the reaction was complete, the outlet die at the bottom of the microcompounder was opened, and the viscous reaction mass was collected in a Teflon cup. Any remaining material was scraped from the barrel once the microcompounder was opened and combined with the initial batch. The combined mass was dissolved in 35 mL of DMSO and precipitated into 200 mL of ethyl acetate. The precipitated polymer was allowed to settle, the solvent was carefully decanted, and the remaining polymer was thoroughly washed with ethyl acetate before being dried at 80°C under dynamic vacuum (<1 mbar) overnight.

PHU_1 polymer with HMDA was synthesized according to the previously published procedure.[6a]

IV.2. PHU based on M_2 and HMDA (PHU_2).

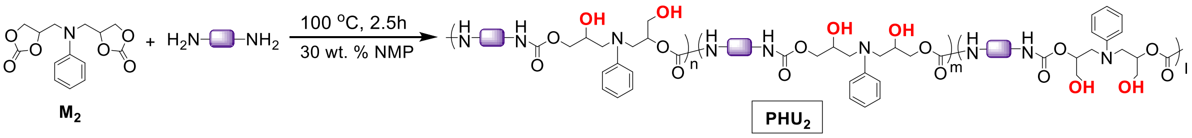

Scheme S3. Synthesis of **PHU_2**.

4,4'-((phenylazanediyl)bis(methylene))bis(1,3-dioxolan-2-one) **M_2** (8 g, 27.3 mmol), HMDA (3.17 g, 27.3 mmol) and anhydrous NMP (3.5 ml, 30wt%) were applied in accordance with the general synthetic method described above. Yield: 6.7 g (60%). M_n (GPC) = 9500 g·mol^{-1}, M_w/M_n = 3.95; T_g (DSC, 5°C/min) = 53°C, T_g (DSC, 10°C/min) = 55°C; T_{onset} (TGA, 5°C/min) = 175°C; Anal. calcd. for $C_{20}H_{31}N_3O_6$ (409.48): C, 58.66%; H, 7.63%; N, 10.26%. Found: C, 57.67%; H, 7.6%; N, 10.14%.

^{1}H NMR (600 MHz, DMSO-d_6) δ 7.24 – 6.96 (m, 4H), 6.89 – 6.77 (m, 1H), 6.71 (dd, J = 32.1, 8.3 Hz, 2H), 6.57 (p, J = 6.7 Hz, 1H), 5.13 – 4.97 (m, 1H), 4.96 – 4.85 (m, 1H), 4.85 – 4.73 (m, 1H), 3.96 – 3.80 (m, 4H), 3.65 – 3.39 (m, 4H), 3.07 – 2.83 (m, 4H), 1.47 – 1.28 (m, 4H), 1.28 – 1.14 (m, 4H).

^{13}C NMR (151 MHz, DMSO-d_6) δ 156.27, 156.21, 155.95, 155.93, 155.90, 148.02, 147.76, 147.72, 147.58, 147.54, 128.97, 115.41, 115.30, 115.13, 111.67, 71.99, 71.65, 71.63, 68.99, 66.39, 66.11, 66.07,

[7] (a) Zubkevich, S. V; Makarov, M.; Dieden, R.; Puchot, L.; Berthé, V.; Westermann, S.; Shaplov, A. S.; Schmidt, D. F. Unique Method for Facile Postsynthetic Modification of Nonisocyanate Polyurethanes. *Macromolecules* **2024**, *57* (5), 2385–2393. https://doi.org/10.1021/acs.macromol.3c02232; (b) Datta Sarma, A.; Zubkevich, S. V; Addiego, F.; Schmidt, D. F.; Shaplov, A. S.; Berthé, V. Synthesis of High-T_g Nonisocyanate Polyurethanes via Reactive Extrusion and Their Batch Foaming. *Macromolecules* **2024**, *57* (7), 3423–3437. https://doi.org/10.1021/acs.macromol.4c00222

66.02, 64.02, 63.93, 60.93, 60.78, 55.23, 54.72, 54.57, 54.18, 51.76, 50.95, 40.43, 40.21, 29.45, 29.39, 26.01.

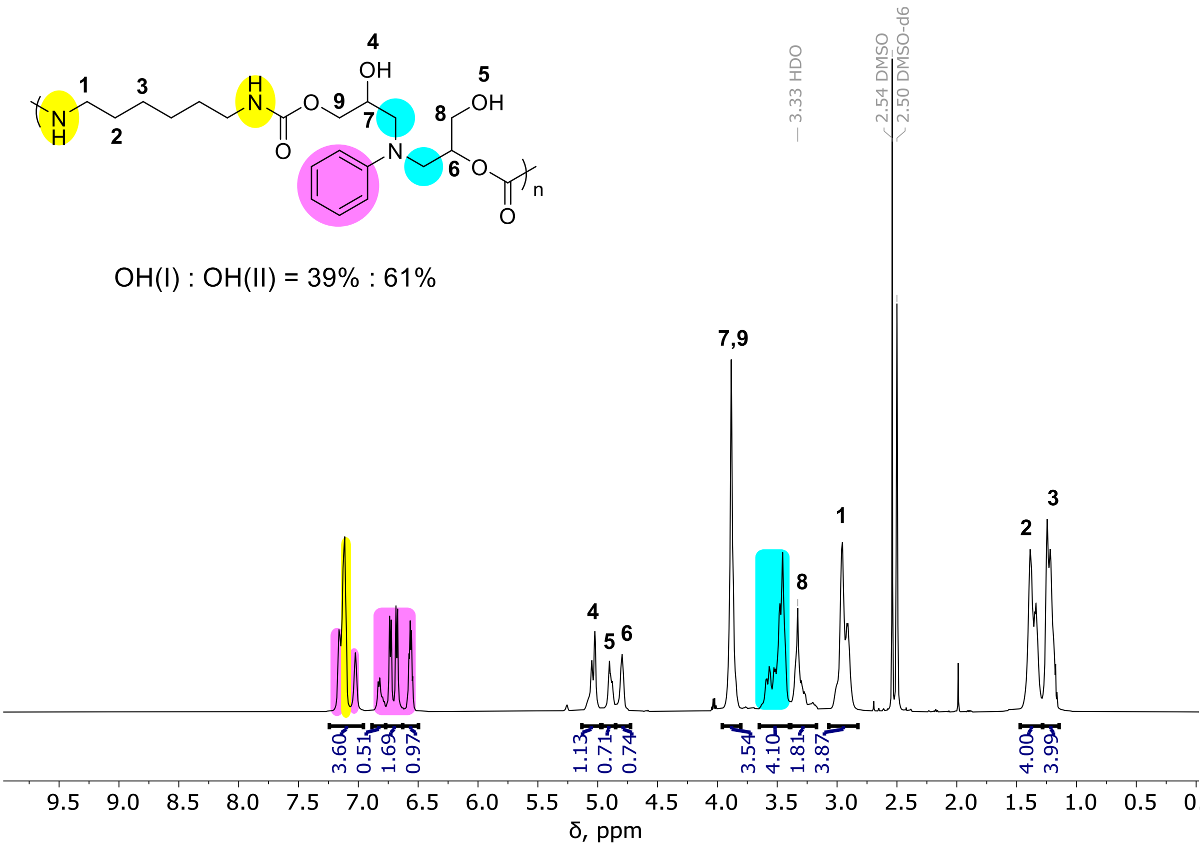

Figure S8. ^{1}H NMR spectrum of **PHU$_2$** in DMSO-d$_6$. The ratio of primary OH(I) **5** and secondary OH(II) **4** groups was calculated from the ratio of corresponding signals.

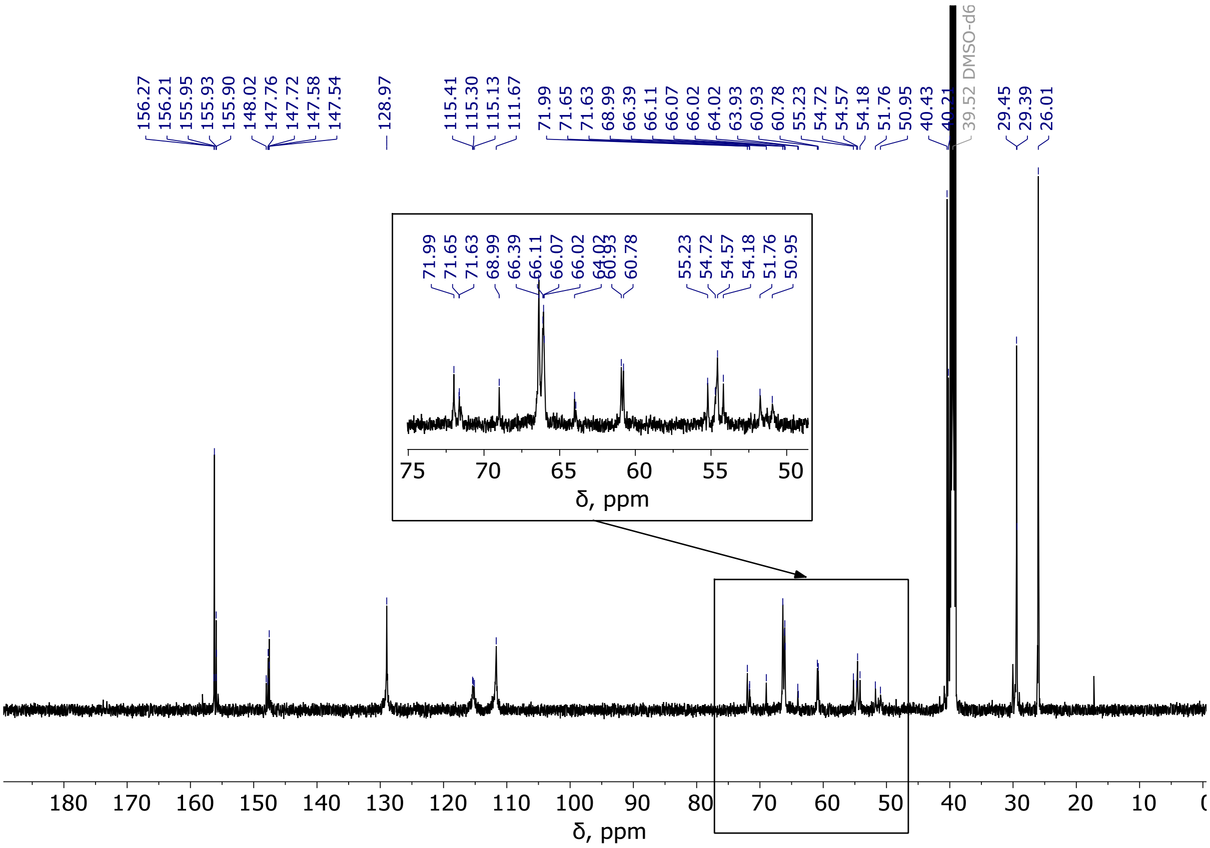

Figure S9. ^{13}C NMR spectrum of **PHU$_2$** in DMSO-d$_6$.

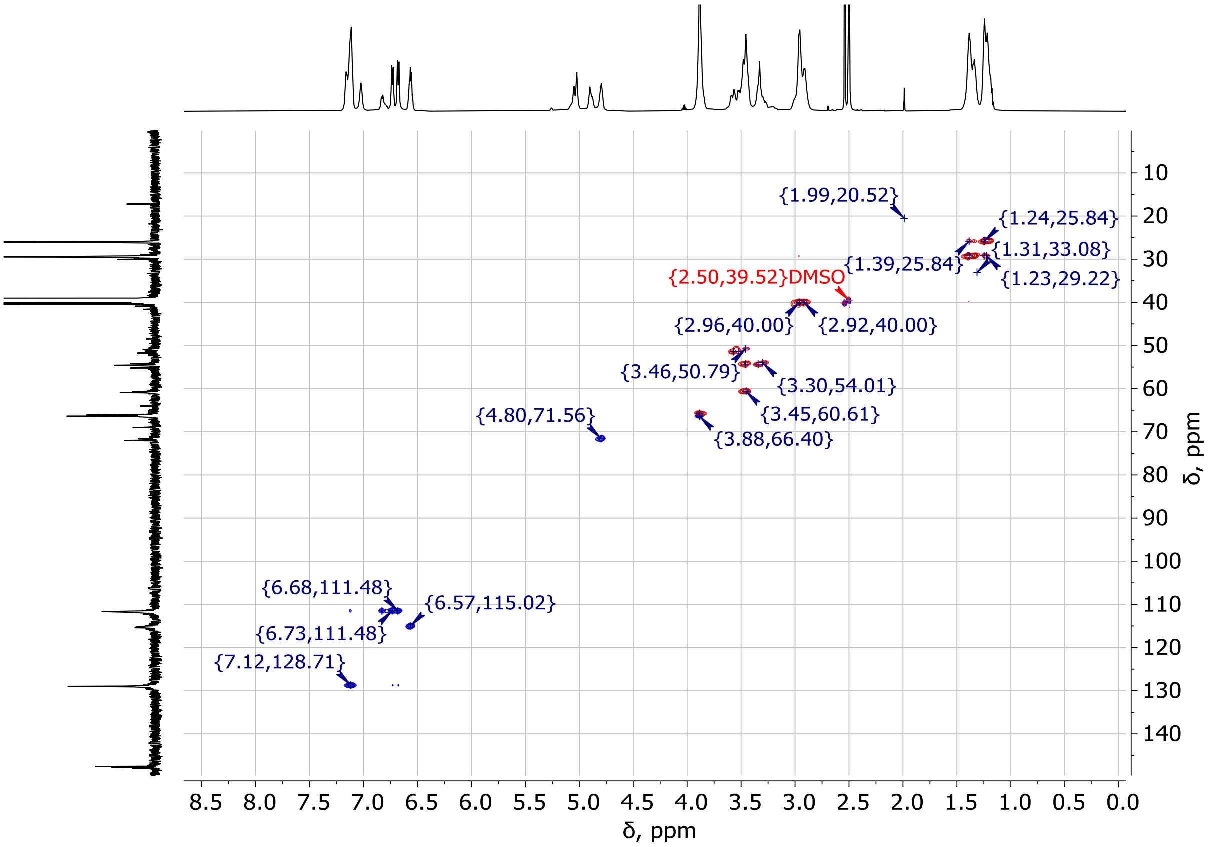

Figure S10. HSQC NMR spectrum of **PHU$_2$** in DMSO-d$_6$.

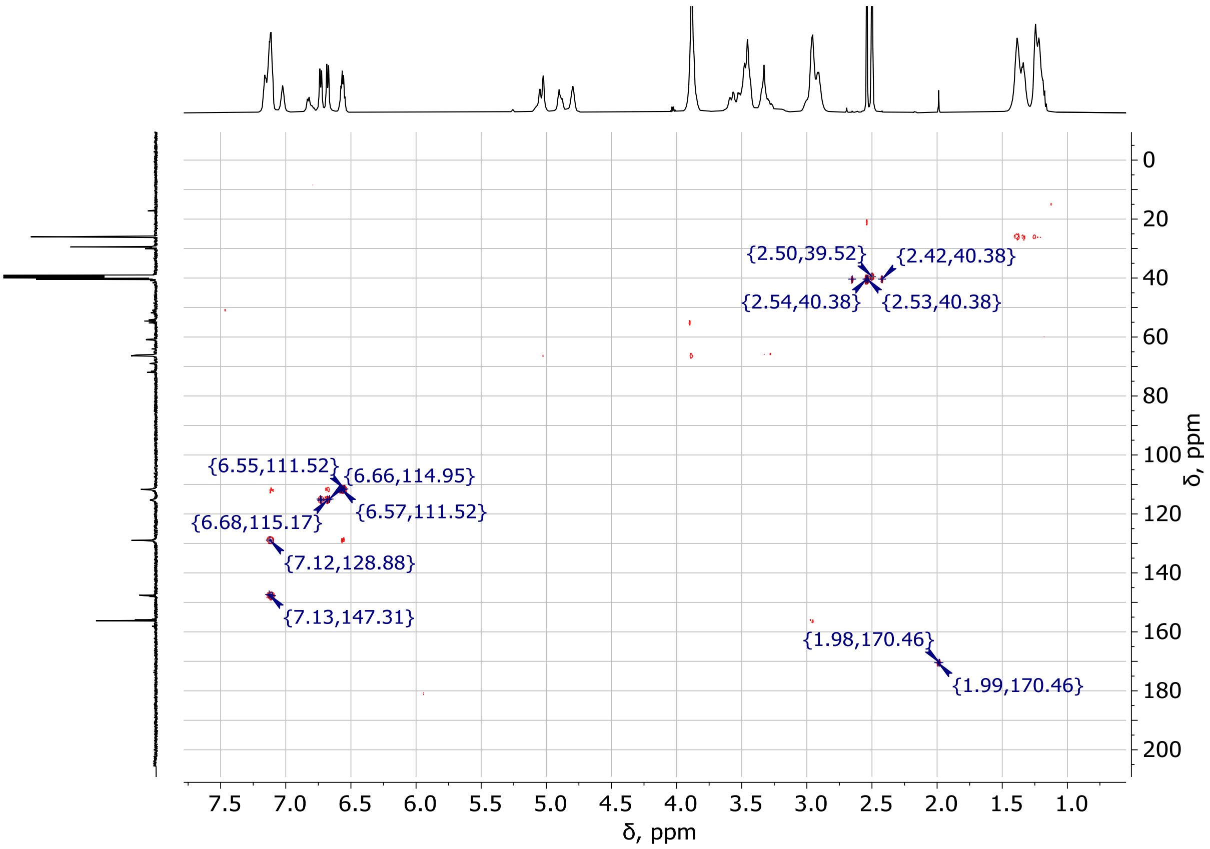

Figure S11. HMBC NMR spectrum of **PHU$_2$** in DMSO-d$_6$.

IV.3. PHU based on **M₃** and HMDA (**PHU₃**).

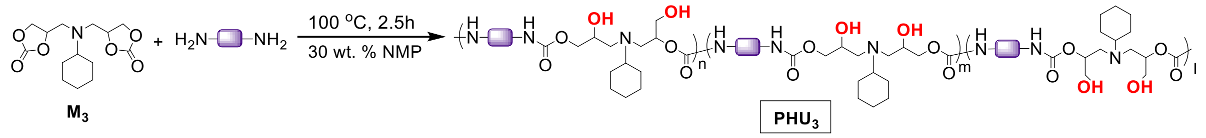

Scheme S4. Synthesis of **PHU₃**.

4,4'-((cyclohexylazanediyl)bis(methylene))bis(1,3-dioxolan-2-one) **M₃** (8 g, 26.7 mmol), HMDA (3.11 g, 26.7 mmol) and anhydrous NMP (3.5 ml, 30wt%) were applied in accordance with the general synthetic method described above. Yield: 8.5 g (76.5%). M_n (GPC) = 16900 g·mol^{-1}, M_w/M_n = 2.5; T_g (DSC, 5°C/min) = 47°C, T_g (DSC, 10°C/min) = 49°C; T_{onset} (TGA, 5°C/min) = 175°C; Anal. calcd. for $C_{20}H_{37}N_3O_6$ (415.53): C, 57.81%; H, 8.98%; N, 10.11%. Found: C, 56.76%; H, 8.74%; N, 10.06%.

^{1}H NMR (600 MHz, DMSO-d$_6$) δ 7.14 – 6.57 (m, 2H), 4.72 – 4.61 (m, 1H), 4.61 – 4.56 (m, 1H), 4.56 – 4.46 (m, 1H), 4.04 – 3.89 (m, 1H), 3.88 – 3.77 (m, 1H), 3.60 (s, 1H), 3.51 – 3.37 (m, 2H), 3.03 – 2.86 (m, J = 6.9 Hz, 4H), 2.66 – 2.55 (m, 1H), 2.48 – 2.28 (m, 3H), 1.77 – 1.49 (m, 5H), 1.36 (t, J = 7.0 Hz, 4H), 1.22 (t, J = 5.6 Hz, 4H), 1.18 – 0.94 (m, 5H).

^{13}C NMR (151 MHz, DMSO-d$_6$) δ 156.36, 156.32, 156.23, 73.80, 73.43, 73.24, 68.17, 67.46, 66.29, 61.35, 61.24, 60.98, 60.76, 60.39, 59.68, 54.88, 54.06, 53.91, 52.35, 51.79, 51.32, 51.09, 40.43, 40.15, 29.44, 28.41, 28.30, 28.06, 26.01, 25.85, 25.75, 25.70.

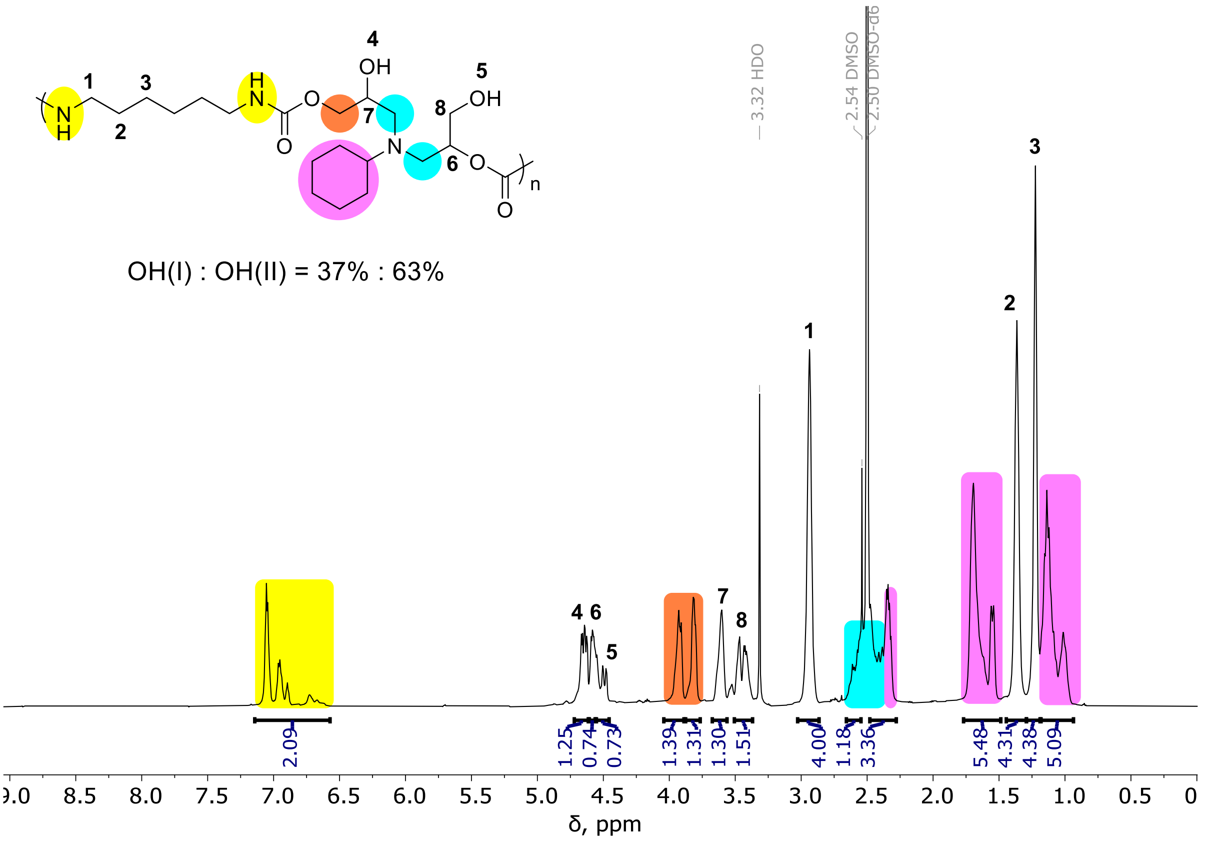

Figure S12. ^{1}H NMR spectrum of **PHU₃** in DMSO-d$_6$. The ratio of primary OH(I) **5** to secondary OH(II) **4** groups was calculated from the ratio of corresponding signals.

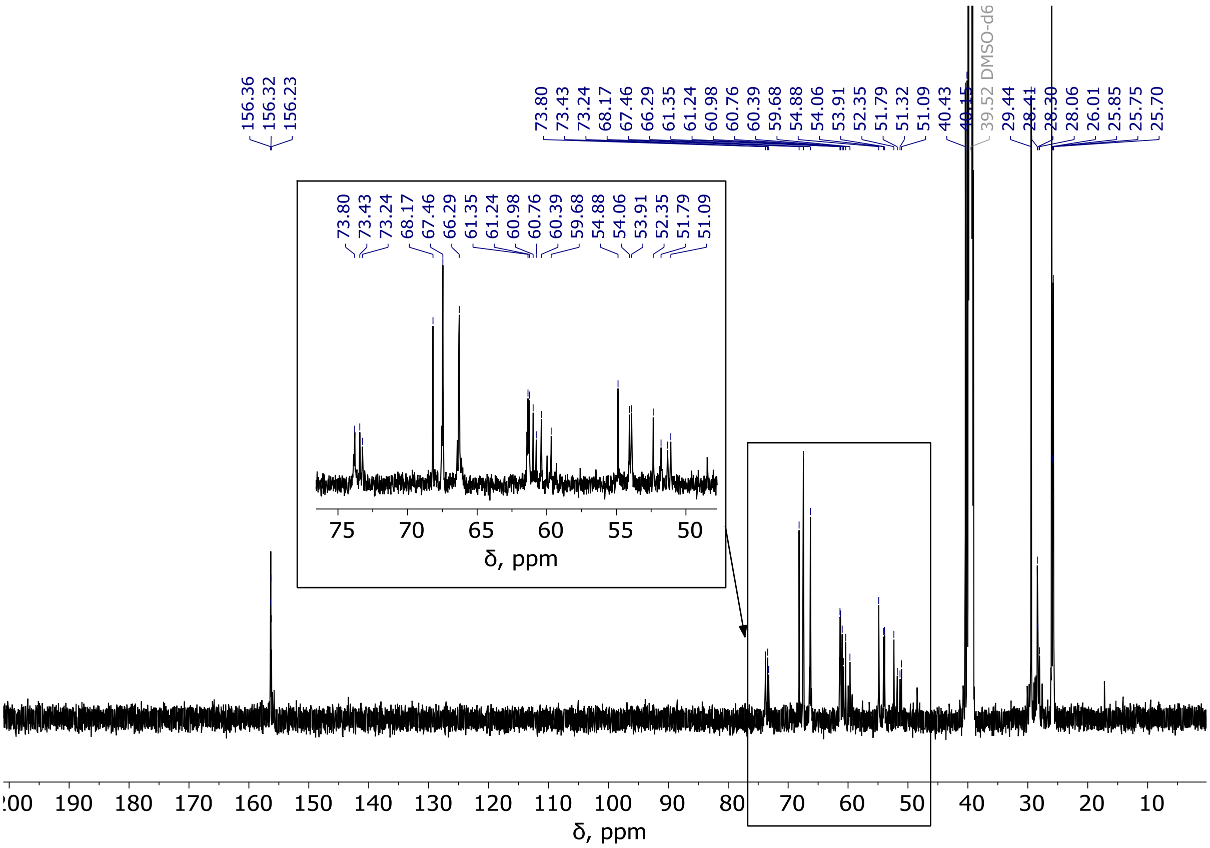

Figure S13. ^{13}C NMR spectrum of **PHU$_3$** in DMSO-d$_6$.

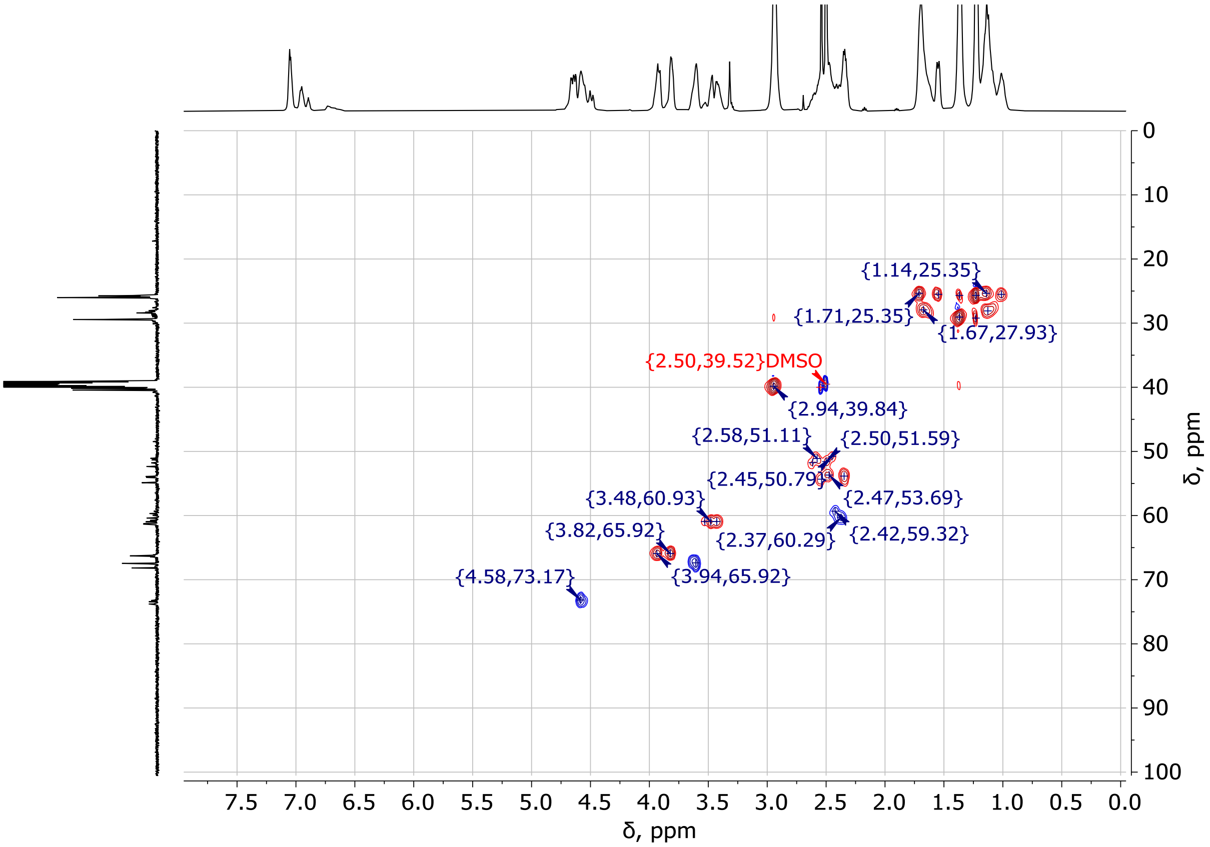

Figure S14. HSQC NMR spectrum of **PHU$_3$** in DMSO-d$_6$.

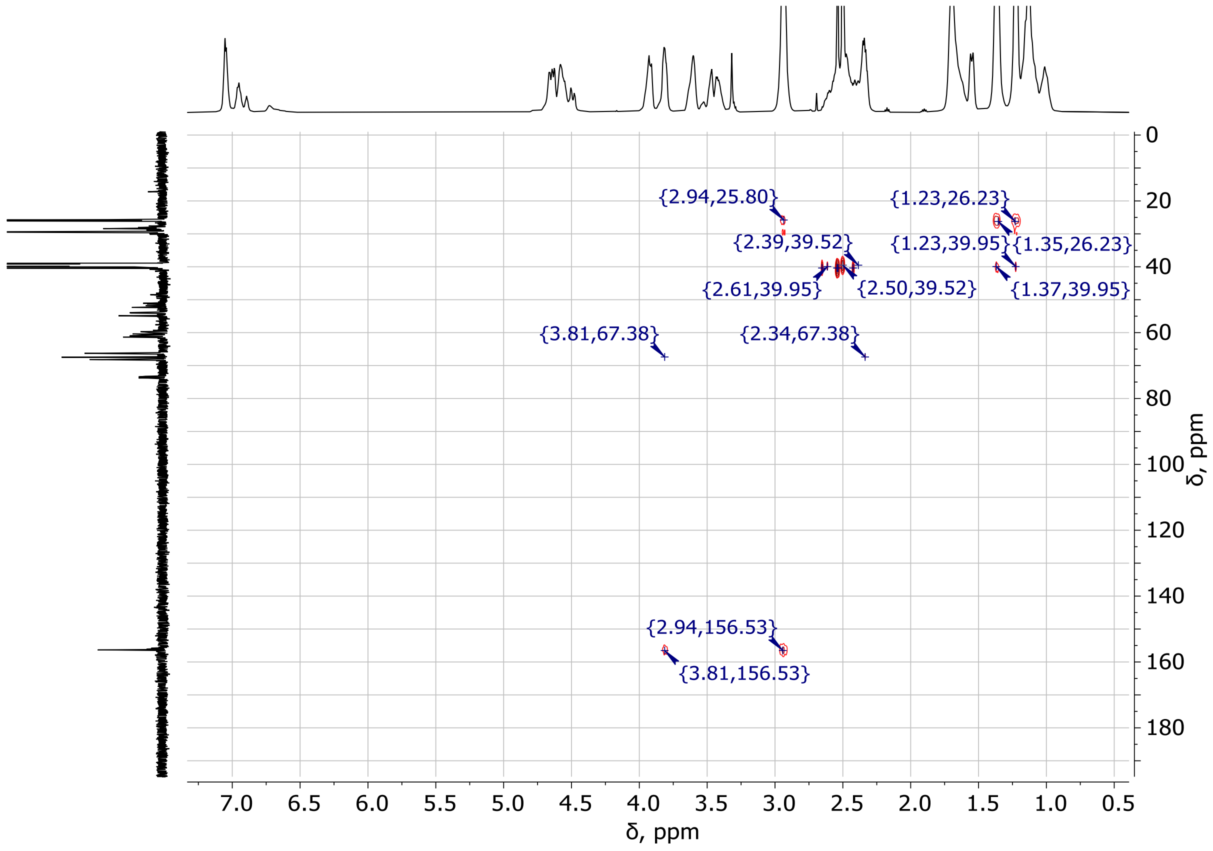

Figure S15. HMBC NMR spectrum of **PHU₃** in DMSO-d₆.

IV.4. Calculation of hydroxyl group content

In accordance with their chemical formulas, the molecular weights (MWs) of the PHU repeating units are calculated as follows:

PHU_1: $C_{12}H_{22}N_2O_6$ = 290.316 g/mol

PHU_2: $C_{20}H_{31}N_3O_6$ = 409.483 g/mol

PHU_3: $C_{20}H_{37}N_3O_6$ = 415.531 g/mol

With two hydroxyl groups per repeating unit, the OH content is calculated as follows:

PHU_1: 2/290.316 = 0.0069 mol/g = 6.9 mmol/g

PHU_2: 2/409.483 = 0.0049 mol/g = 4.9 mmol/g

PHU_3: 2/415.531 = 0.0048 mol/g = 4.8 mmol/g

V. Synthesis and characterization of PHU vitrimers.

V.1. General synthetic procedure.

Each PHU polymer was dissolved in 15 mL of DMF in a 50 mL round-bottom flask. A calculated amount of 1,4-phenylenediboronic acid (0.5 mol equivalent per PHU repeating unit) was then dissolved in 5 mL of DMF and added dropwise to the stirred polymer solution at room temperature. During this addition, the **PHU_1** and **PHU_3** solutions gelled immediately, whereas the **PHU_2** solution remained liquid but cloudy. The resulting gels (**PHU_1** and **PHU_3**) and the cloudy **PHU_2** solution were transferred to PTFE Petri dishes and dried in air at 60°C for 2 hours, followed by 80°C for an additional 2 hours to remove most of the solvent. The cross-linked films were then dried at 100°C under dynamic vacuum (<1 mbar) for 10 hours, and subsequently at 120°C/0.5–1 mbar in a Buchi B-585 oven (Buchi Glass Drying Oven, Buchi) filled with P_2O_5 for 5 days to ensure complete solvent removal. The vitrimer samples obtained were ball-milled at room temperature using a Retsch CryoMill equipped with a 50 mL stainless steel grinding jar and one 20 mm stainless steel ball, operated at 30 Hz for 2 minutes. The resulting fine powder was dried again at 120°C and 0.5–1 mbar in B-585 oven overnight. Finally, the samples were stored in sealed vials in a desiccator containing anhydrous $CaSO_4$ (Drierite™).

V.2. **PHU_1-V**.

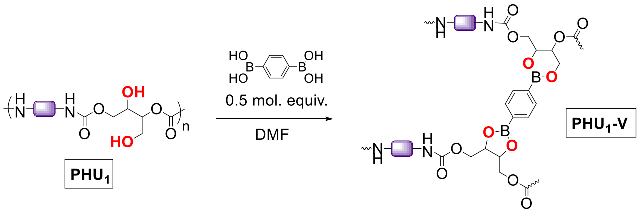

Scheme S5. Synthesis of **PHU_1-V**.

PHU_1 (2 g, 6.90 mmol) and 1,4-phenylenediboronic acid (0.572 g, 3.45 mmol) were used. Yield: 2.25 g (97%). T_g (DSC, 10°C/min) = 80°C, T_g (DSC, 15°C/min) = 84°C, T_g (DSC, 20°C/min) = 88°C; T_{onset} (TGA, 5°C/min) = 175°C; Anal. calcd. for $C_{30}H_{44}N_4O_{12}B_2$ (674.318): C, 53.44%; H, 6.58%; B, 3.21%. Found: C, 53.23%; H, 6.75%; B, 3.19%.

V.3. **PHU_2-V**.

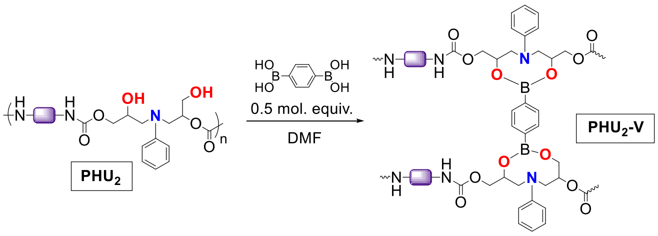

Scheme S6. Synthesis of **PHU_2-V**.

PHU_2 (2 g, 4.88 mmol) and 1,4-phenylenediboronic acid (0.405 g, 2.44 mmol) were used. Yield: 2.18 g (98%). T_g (DSC, 10°C/min) = 81°C, T_g (DSC, 15°C/min) = 85°C, T_g (DSC, 20°C/min) = 87°C; T_{onset} (TGA, 5°C/min) = 180°C;

V.4. **PHU$_3$-V**.

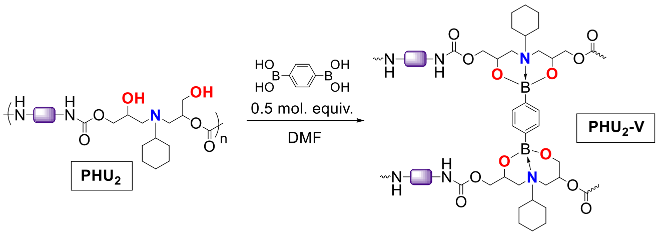

Scheme S6. Synthesis of **PHU$_3$-V**.

PHU$_3$ (2 g, 4.81 mmol) and 1,4-phenylenediboronic acid (0.399 g, 2.41 mmol) were used. Yield: 2.17 g (97%). T_g (DSC, 10°C/min) = 109°C, T_g (DSC, 15°C/min) = 108°C, T_g (DSC, 20°C/min) = 111°C; T_{onset} (TGA, 5°C/min) = 175°C;

VI. Solid state NMR.

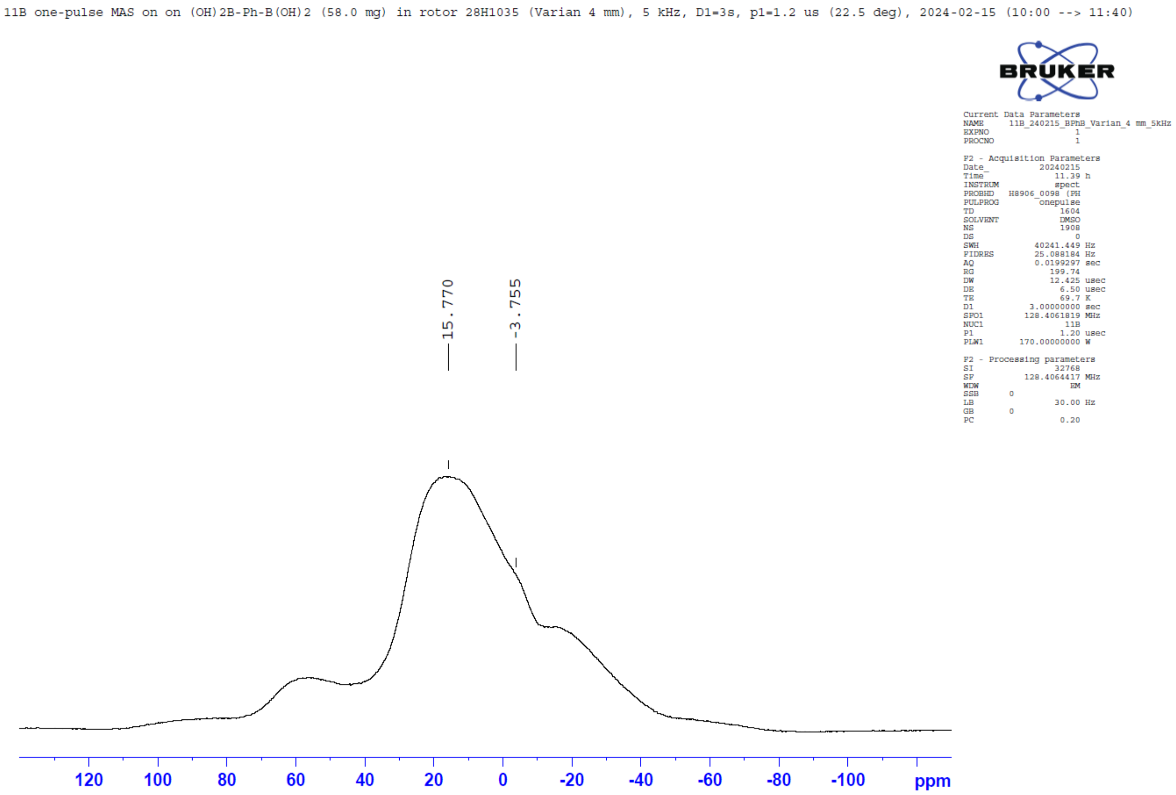

Figure S16. Single-pulse ^{11}B MAS NMR (5 kHz) spectrum of 1,4-phenylenediboronic acid.

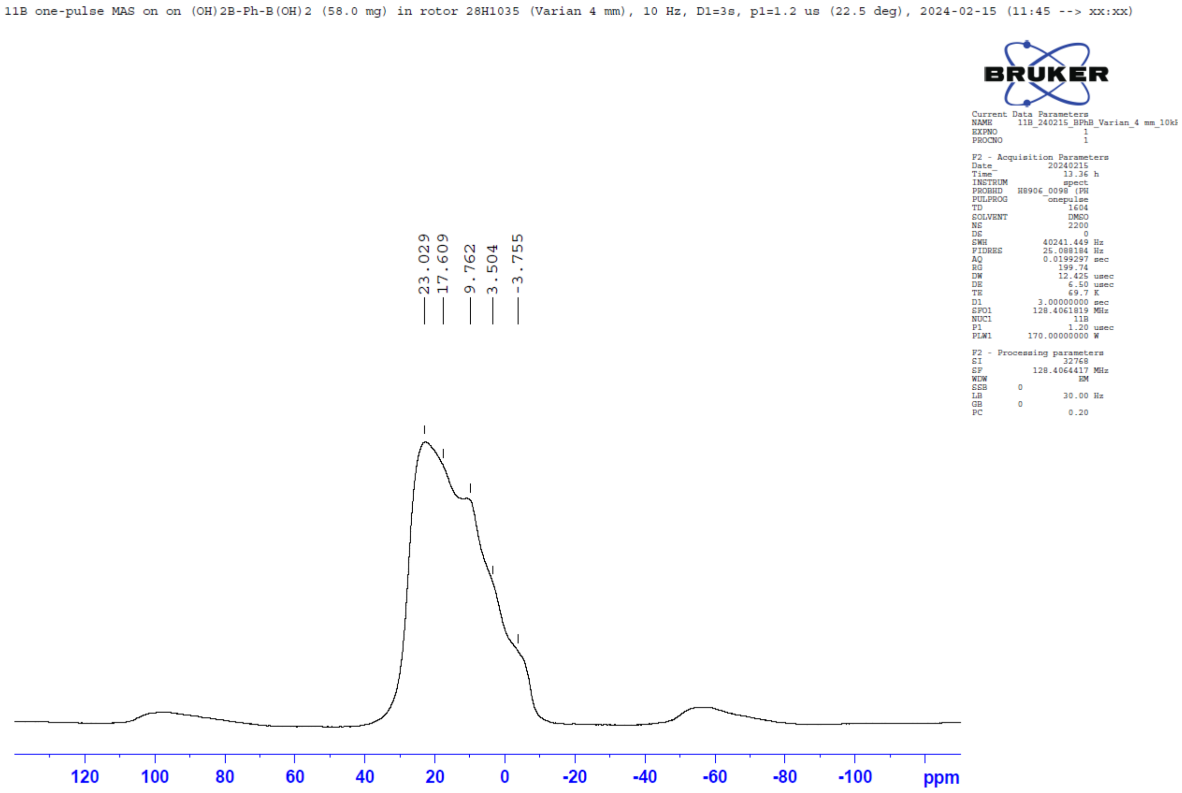

Figure S17. Single-pulse ^{11}B MAS NMR (10 kHz) spectrum of 1,4-phenylenediboronic acid. Peak assignments are given for singularities in the 2nd order quadrupolar pattern (spin I=3/2) not completely averaged out by MAS.

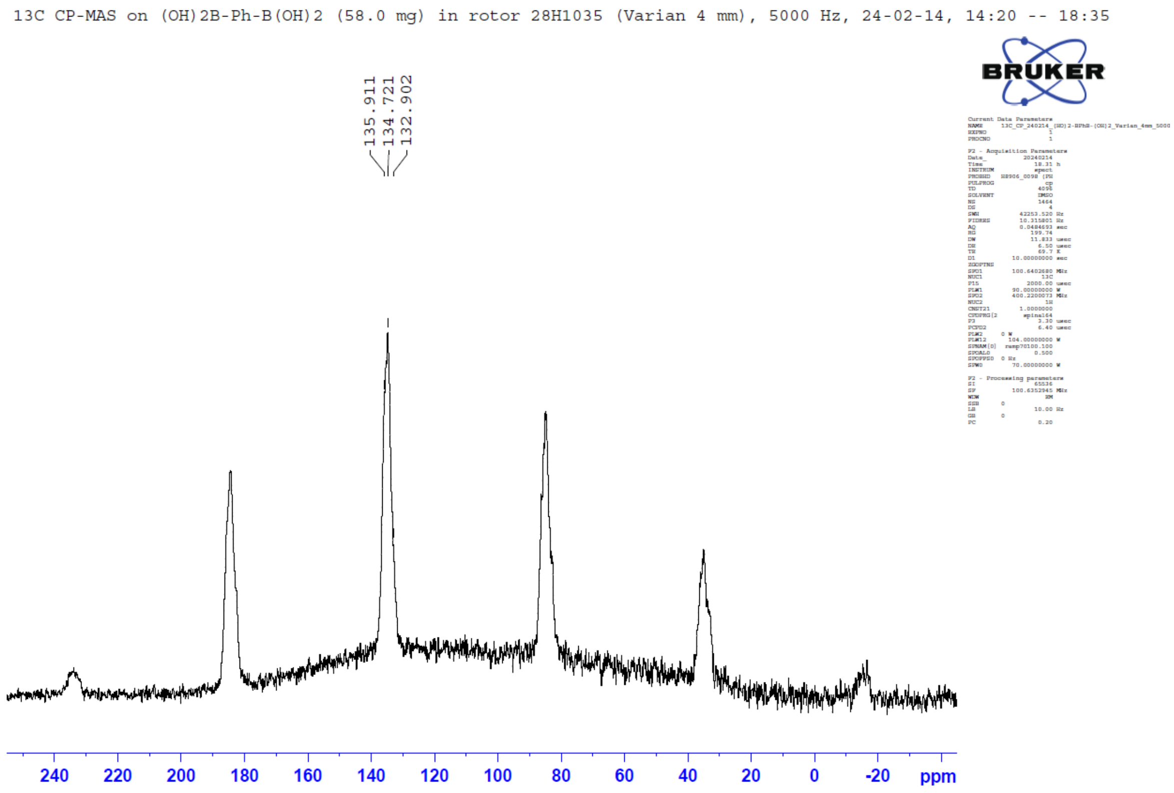

Figure S18. ^{13}C CP-MAS NMR (5 kHz) spectrum of 1,4-phenylenediboronic acid.

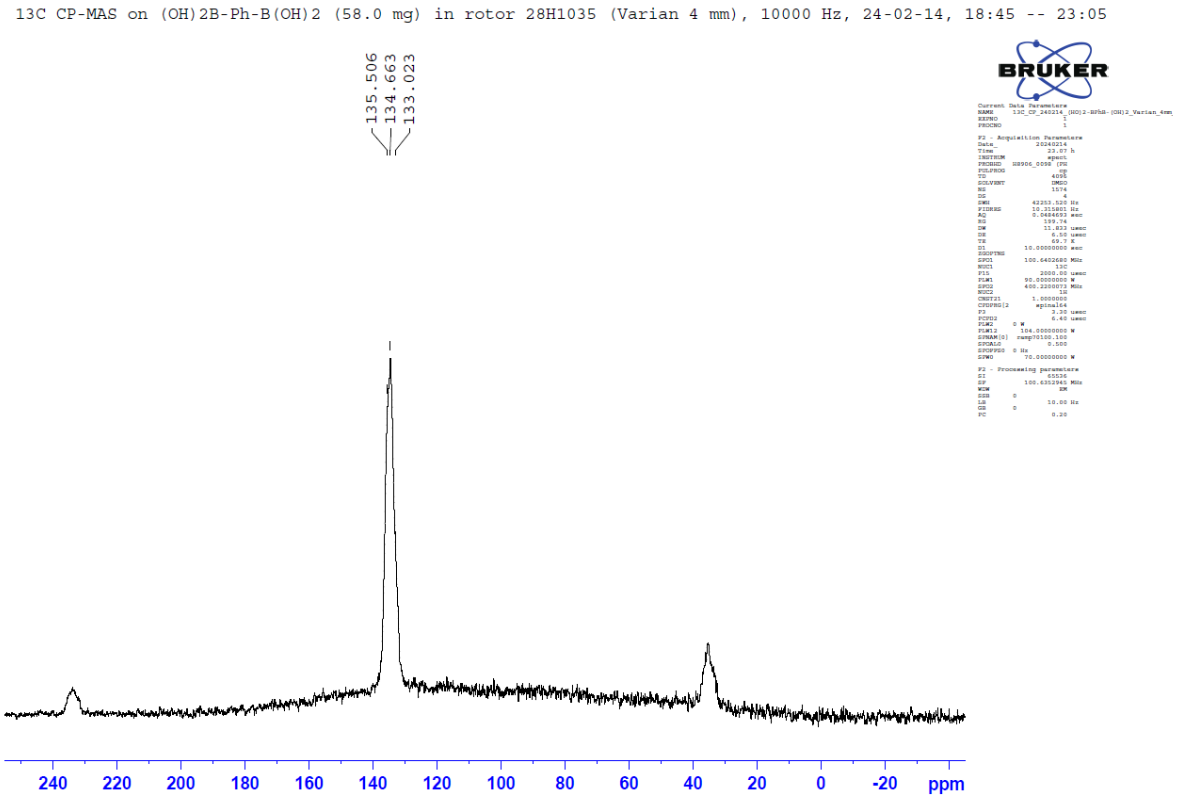

Figure S19. ^{13}C CP-MAS NMR (10 kHz) spectrum of 1,4-phenylenediboronic acid.

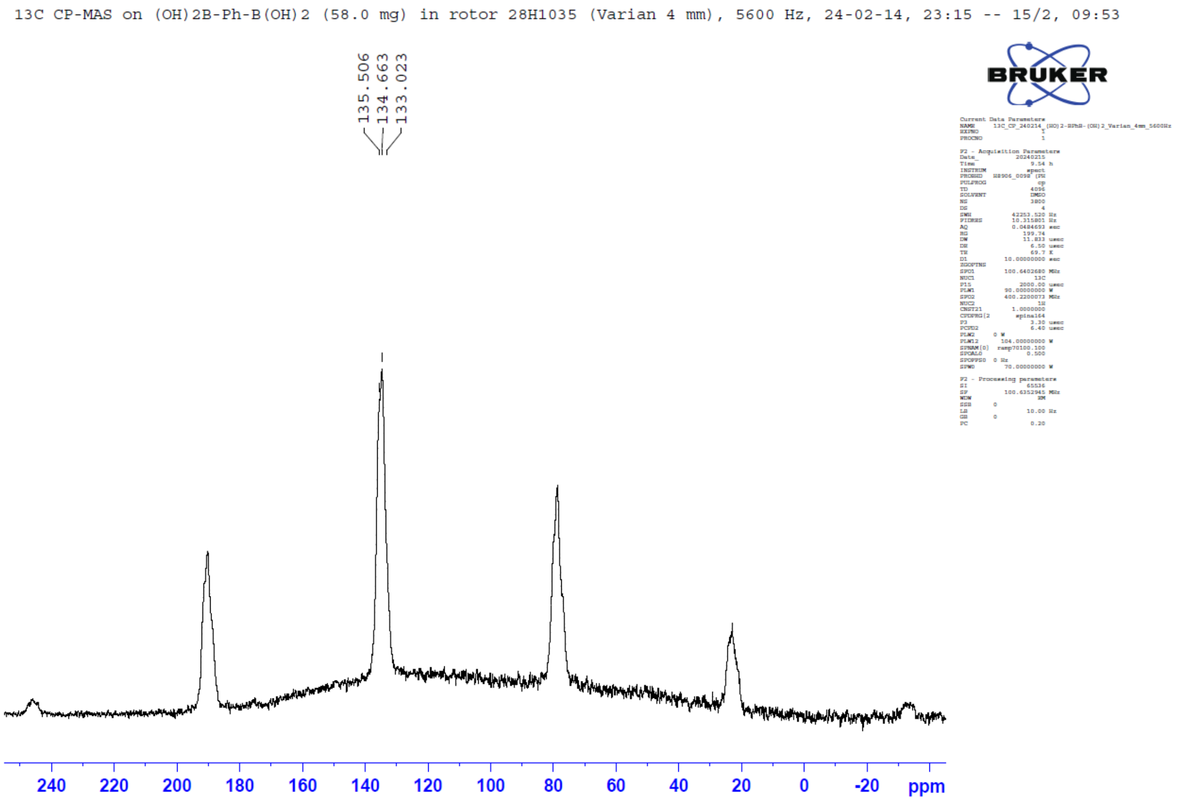

Figure S20. ^{13}C CP-MAS NMR (5.6 kHz) spectrum of 1,4-phenylenediboronic acid.

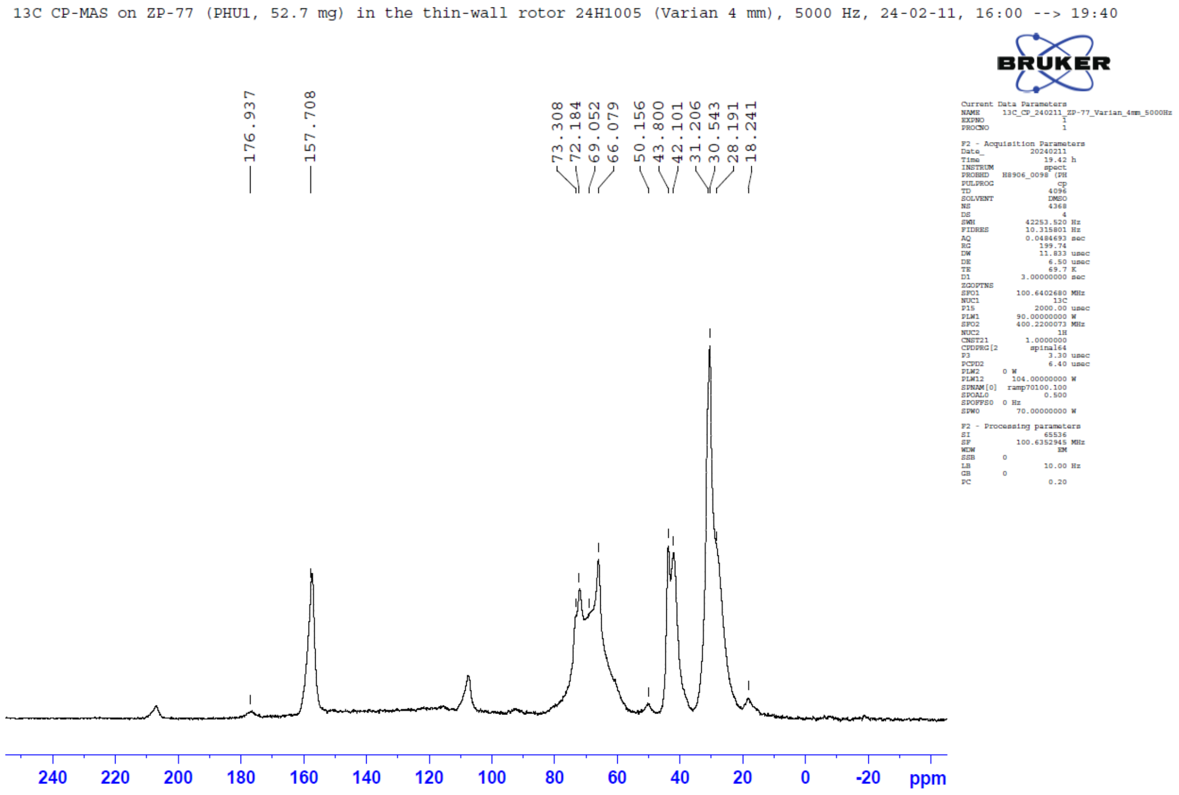

Figure S21. ^{13}C CP-MAS NMR (5 kHz) spectrum of **PHU$_1$**. Spinning sideband peaks are not assigned.

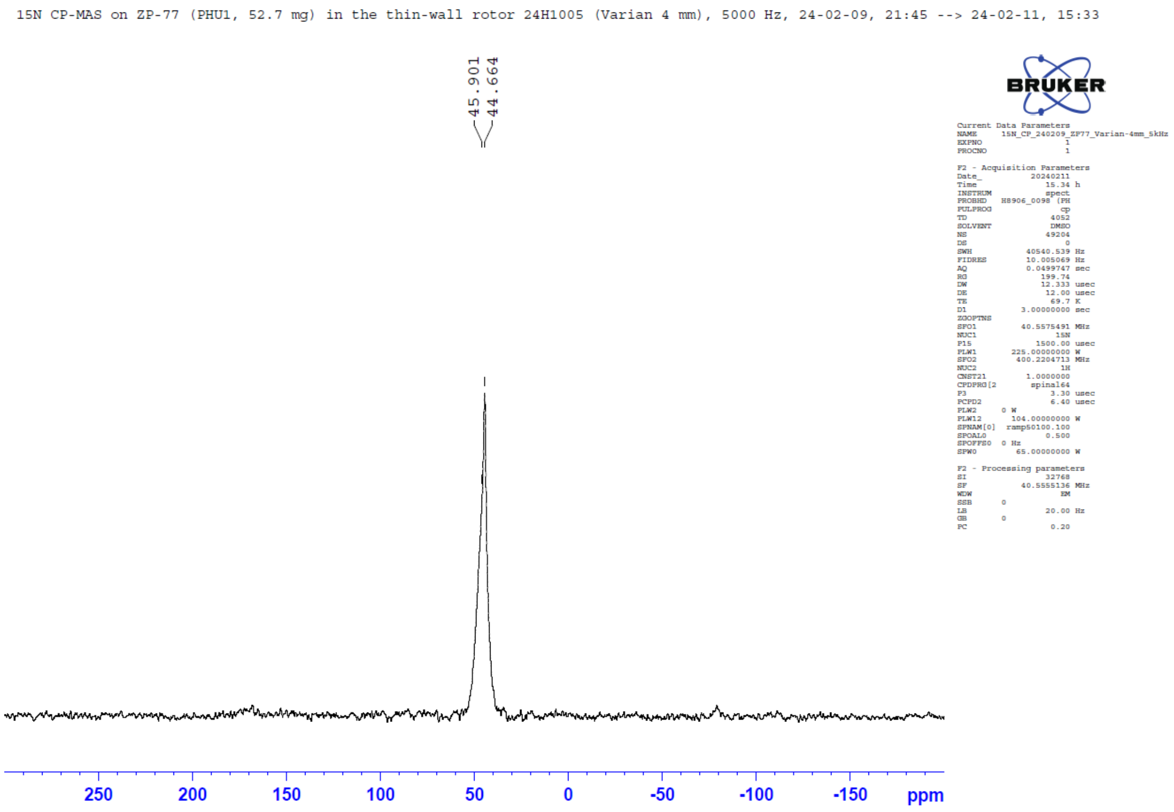

Figure S22. ^{15}N CP-MAS NMR (5 kHz) spectrum of **PHU$_1$**. ±1 spinning sideband peaks are not assigned.

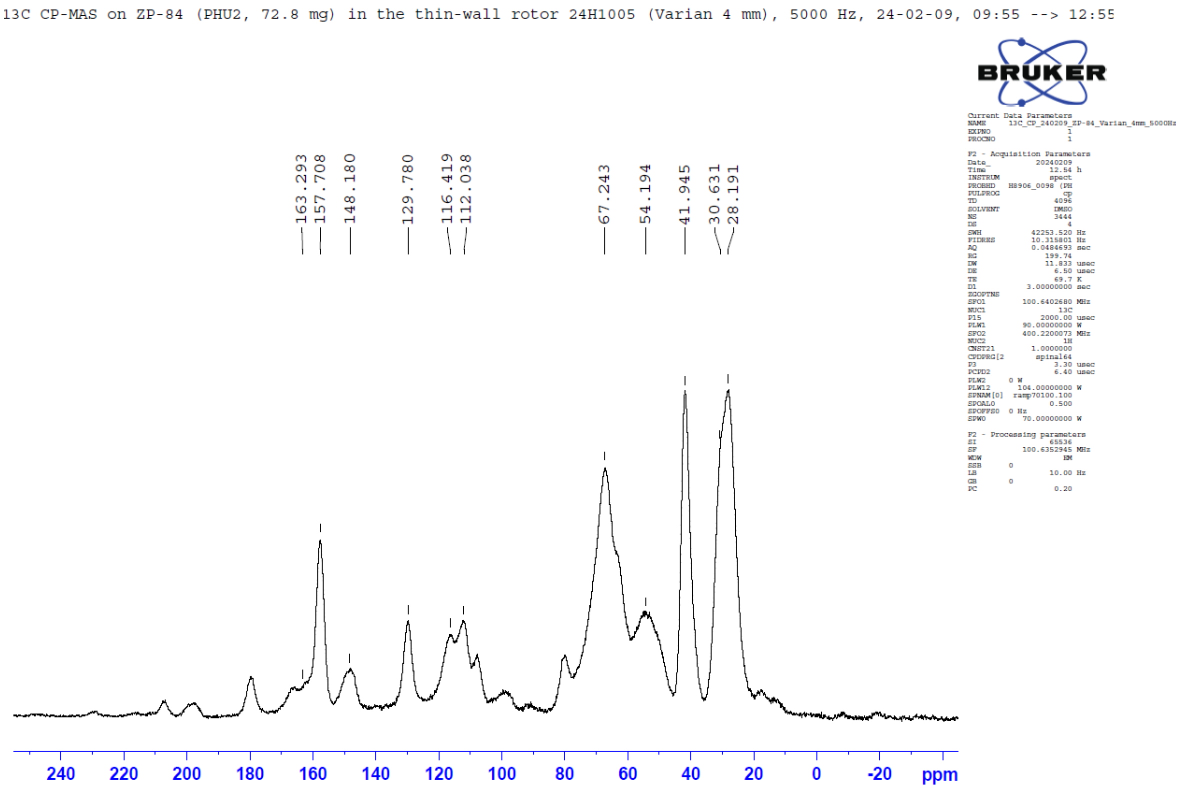

Figure S23. ^{13}C CP-MAS NMR (5 kHz) spectrum of **PHU$_2$**. Spinning sideband peaks are not assigned.

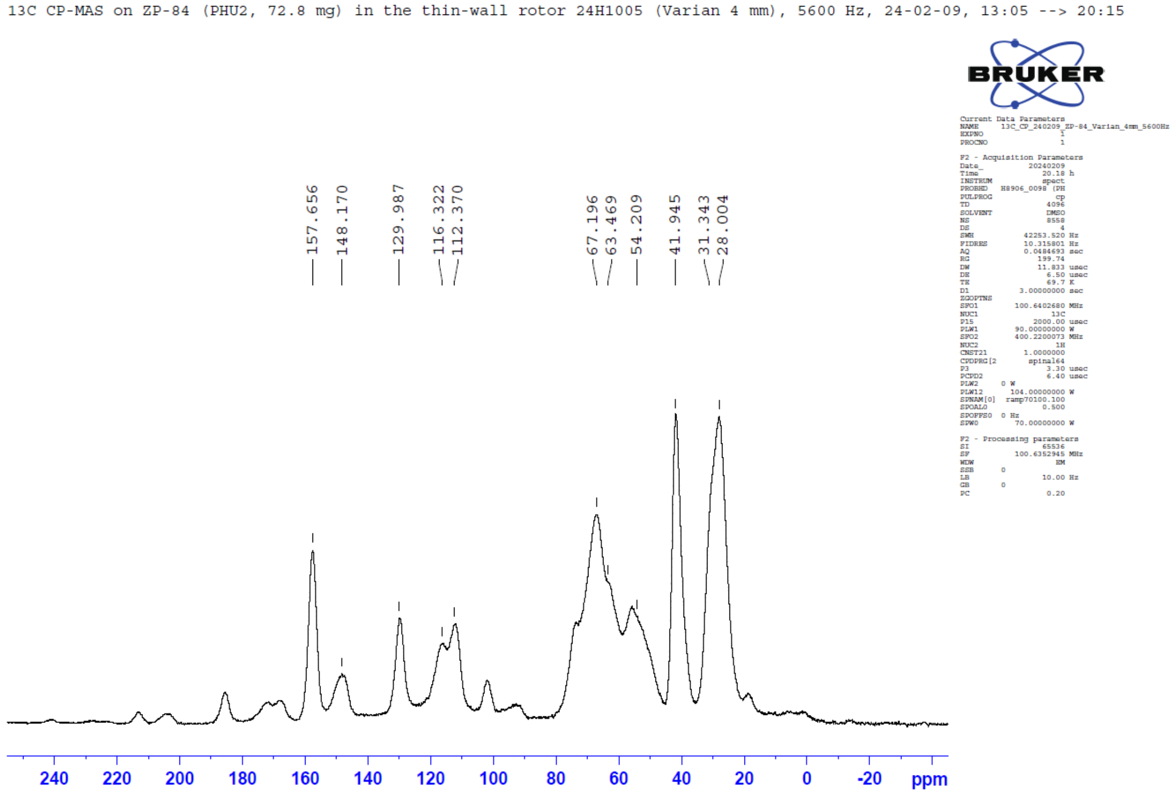

Figure S24. ^{13}C CP-MAS NMR (5.6 kHz) spectrum of **PHU₂**. Spinning sideband peaks are not assigned.

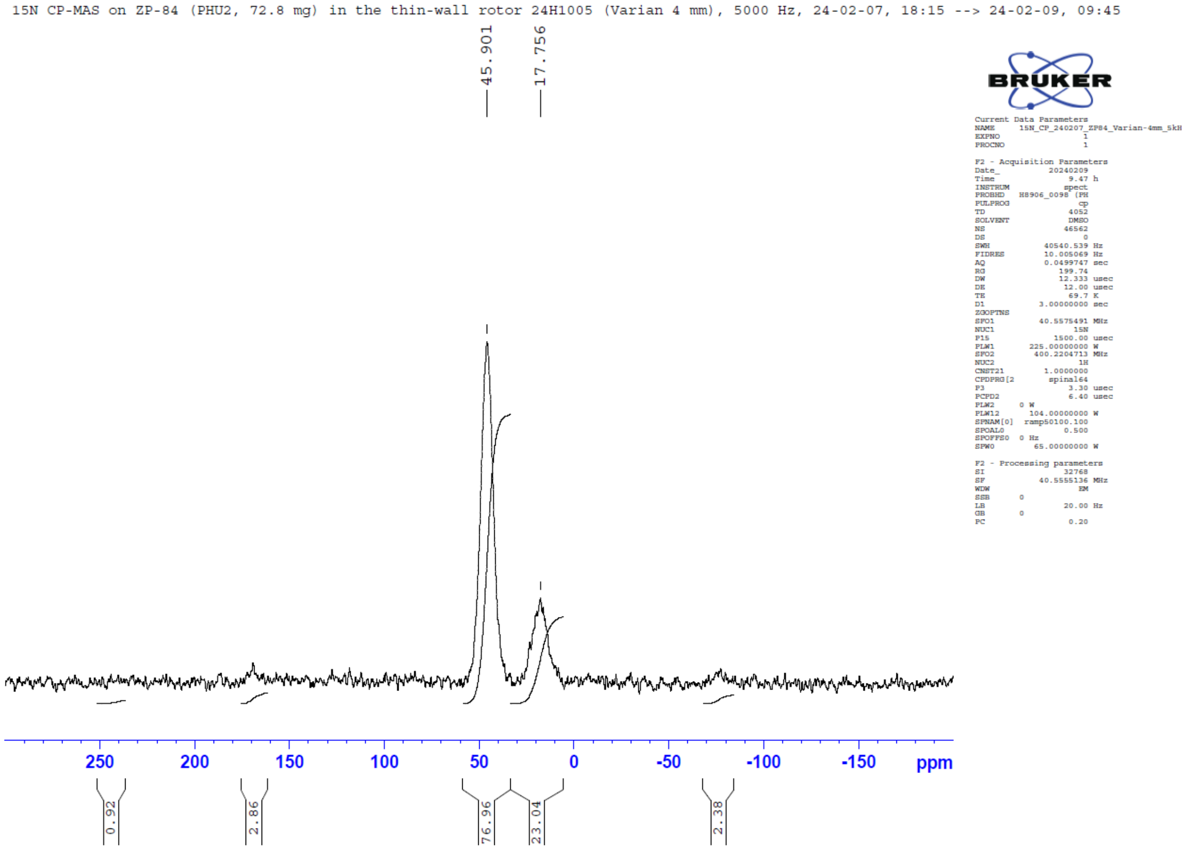

Figure S25. ^{15}N CP-MAS NMR (5 kHz) spectrum of **PHU$_2$**. ±1 spinning sideband peaks are not assigned but are integrated. The baseline is also integrated to estimate the signal-to-noise ratio for different resonances.

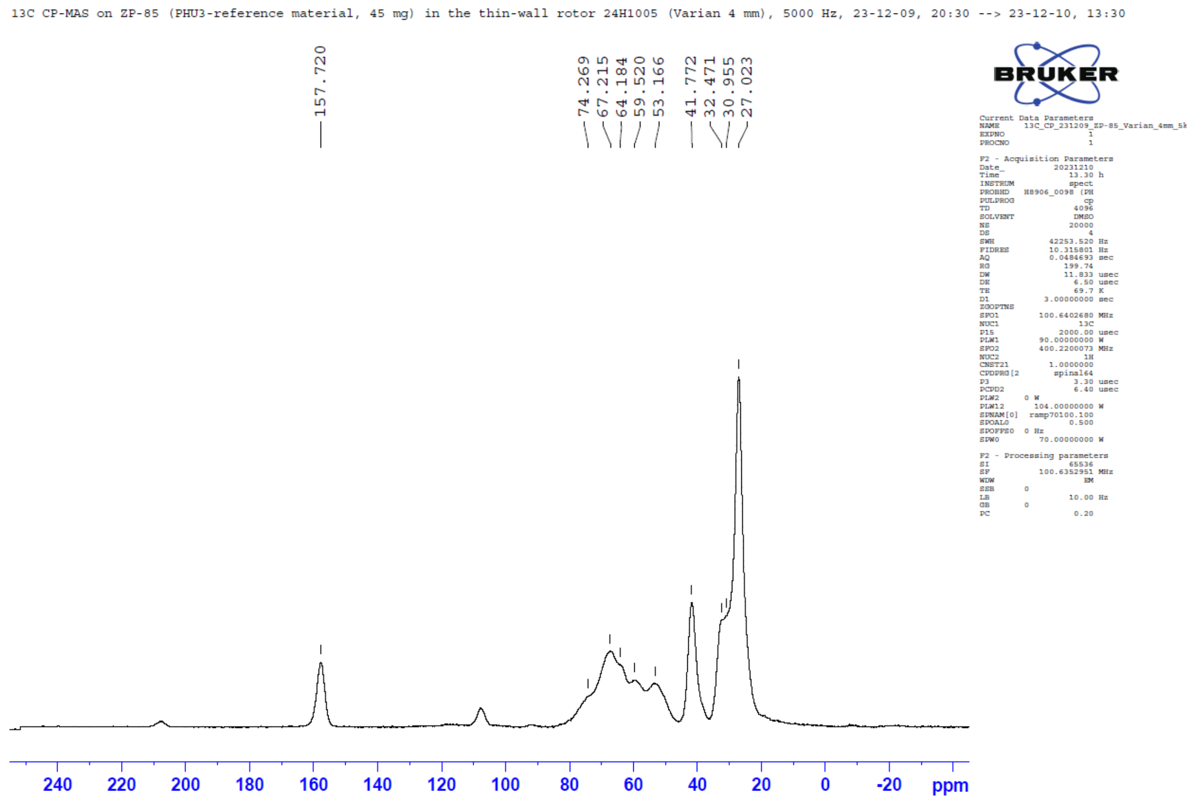

Figure S26. ^{13}C CP-MAS NMR (5 kHz) spectrum of **PHU₃**. Spinning sideband peaks are not assigned.

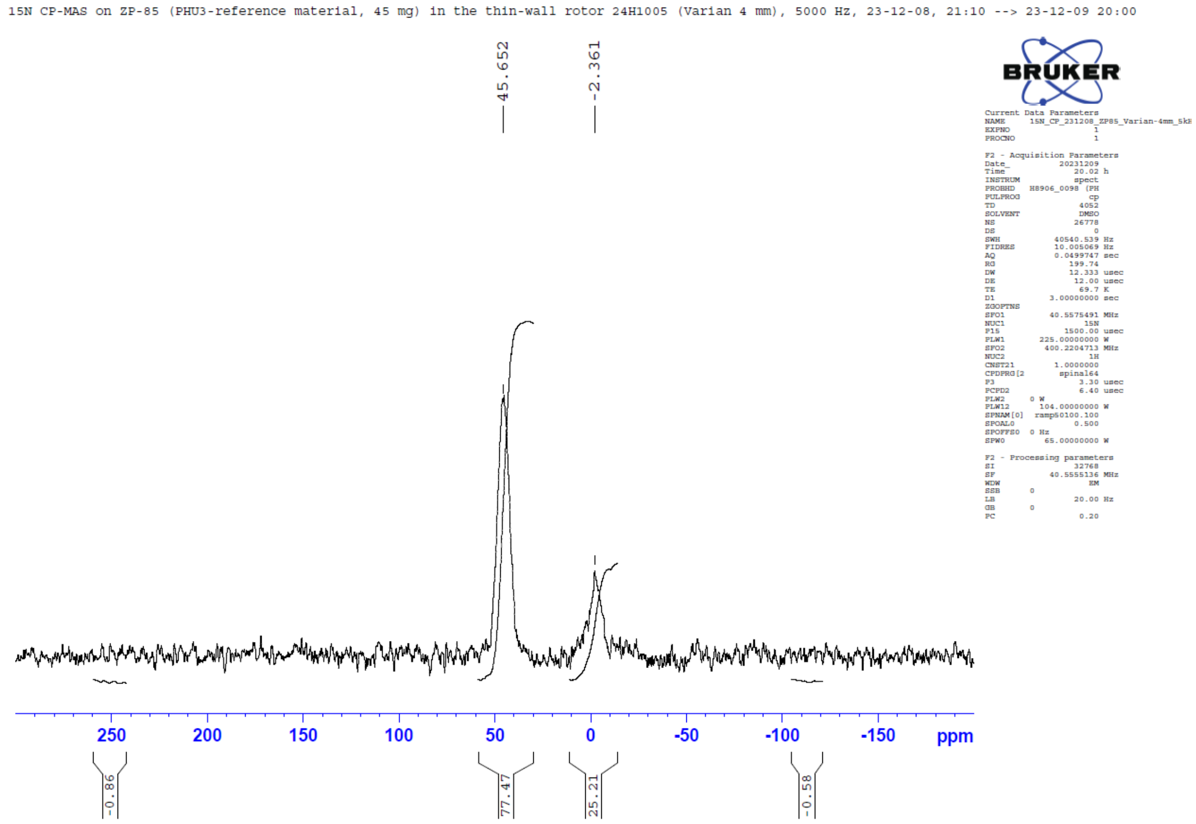

Figure S27. ^{15}N CP-MAS NMR (5 kHz) spectrum of **PHU₃**. The baseline is integrated in two places to estimate the signal-to-noise for different resonances.

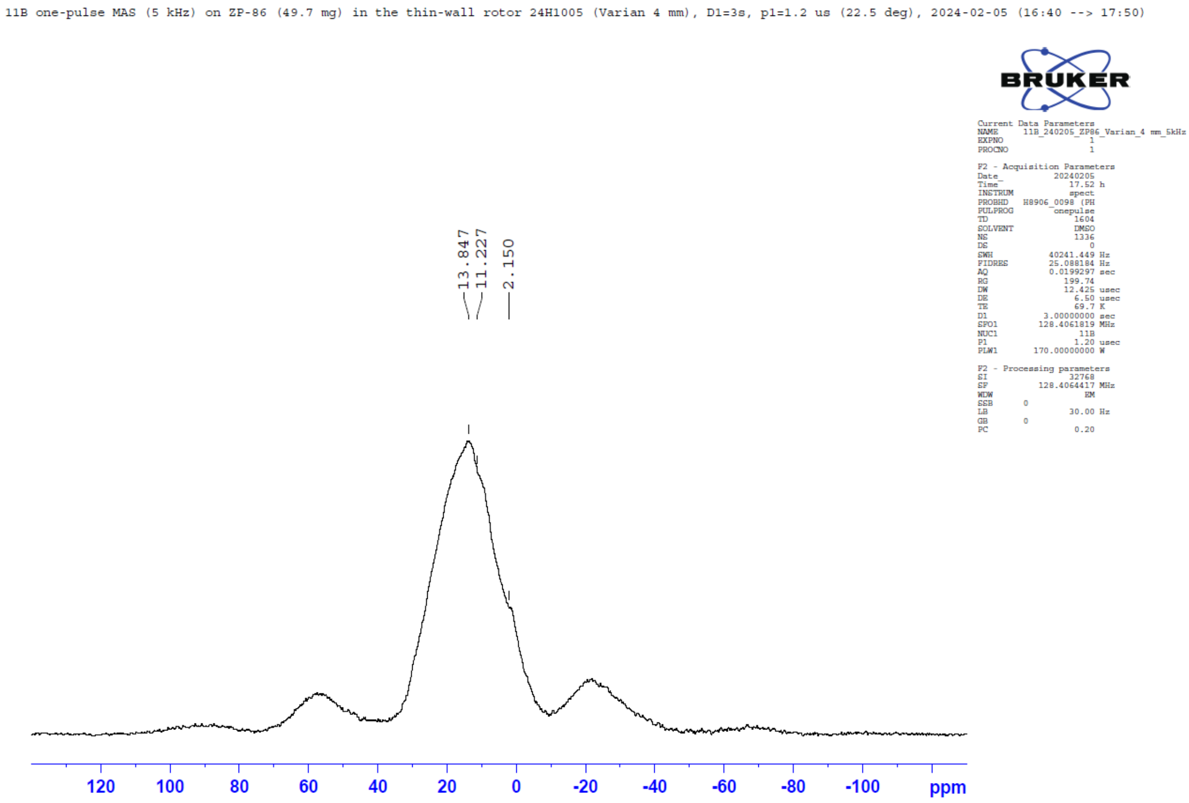

Figure S28. ^{11}B MAS NMR (5 kHz) spectrum of **PHU$_1$-V**.

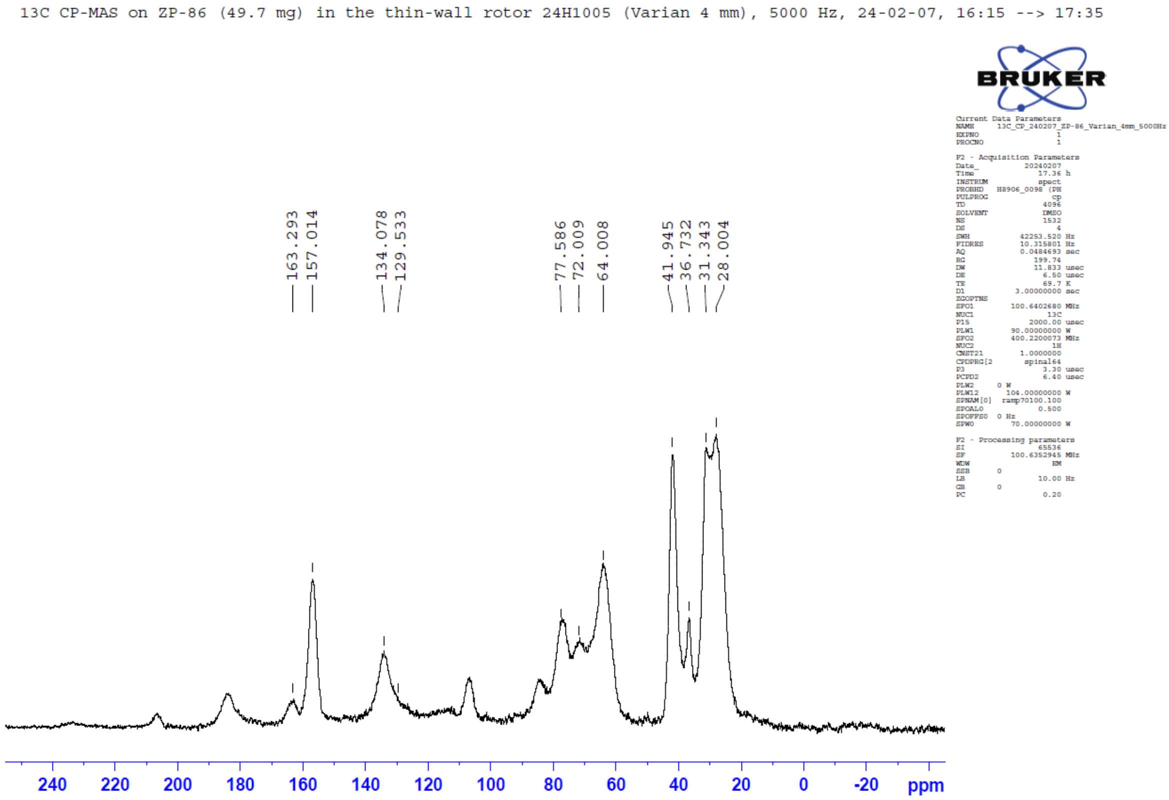

Figure S29. ^{13}C CP-MAS NMR (5 kHz) spectrum of **PHU$_1$-V**. Spinning sideband peaks are not assigned.

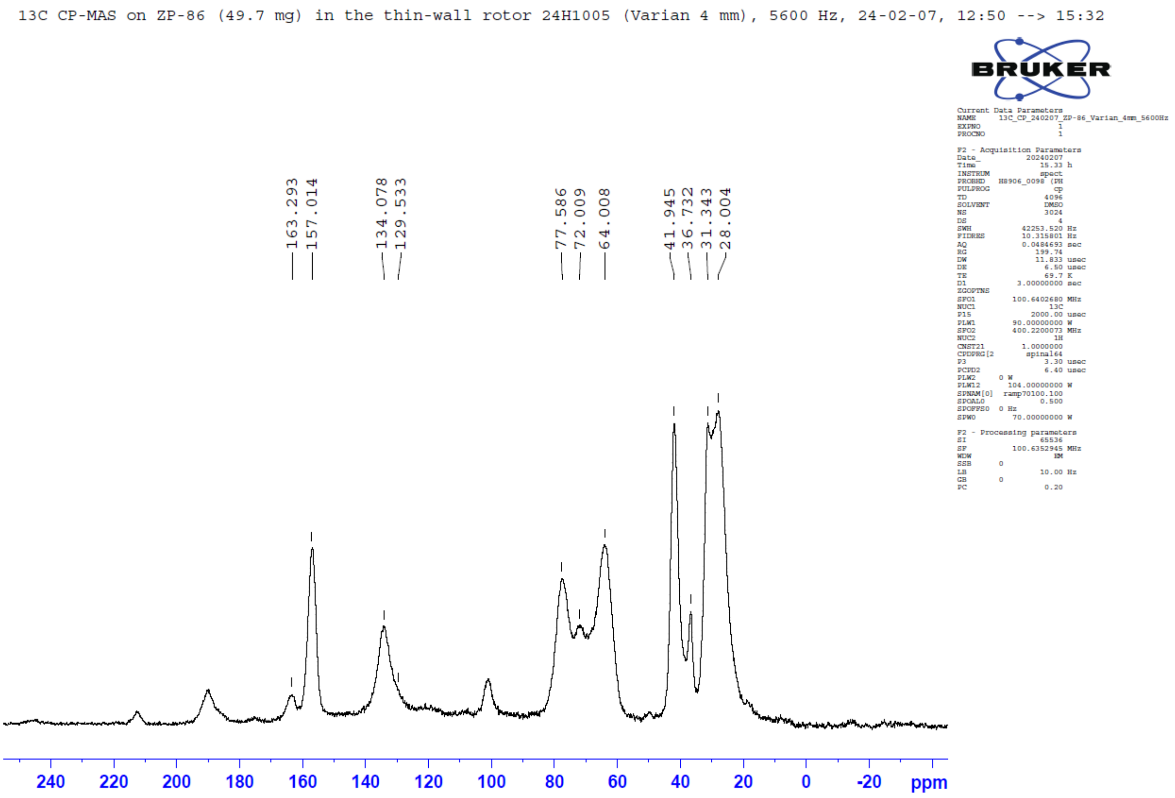

Figure S30. ^{13}C CP-MAS NMR (5.6 kHz) spectrum of **PHU$_1$-V**. Spinning sideband peaks are not assigned.

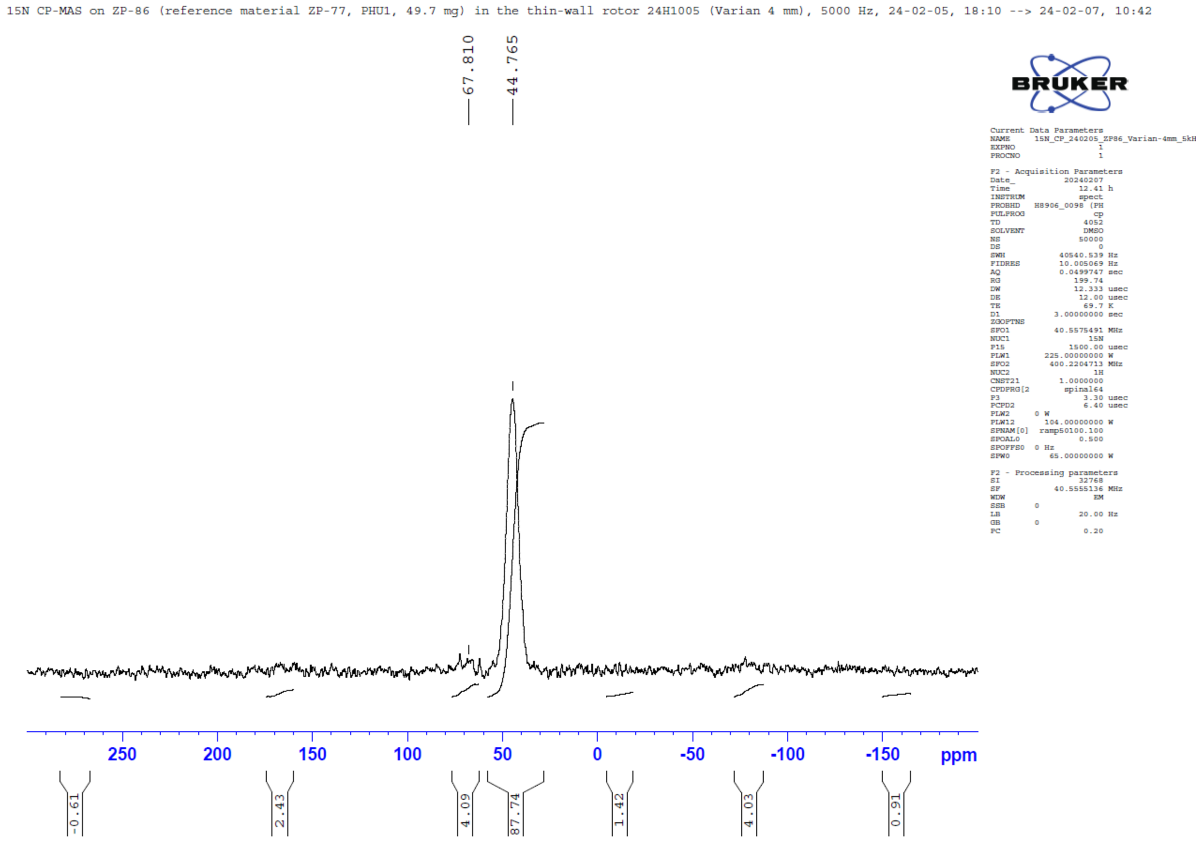

Figure S31. ^{15}N CP-MAS NMR (5 kHz) spectrum of **PHU$_1$-V**. ±1 spinning sideband peaks are not assigned but are integrated. The baseline is also integrated at three intervals to estimate the signal-to-noise for different resonances.

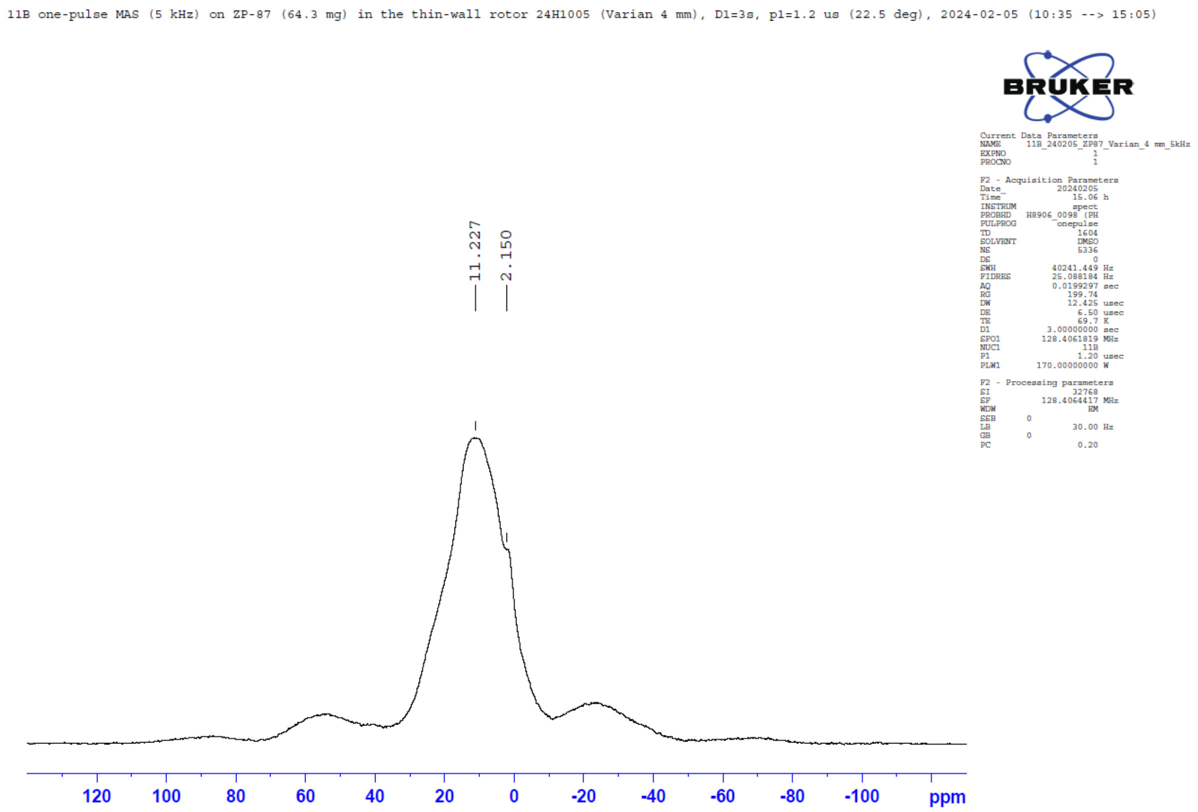

Figure S32. ^{11}B MAS NMR (5 kHz) spectrum of **PHU$_2$-V**.

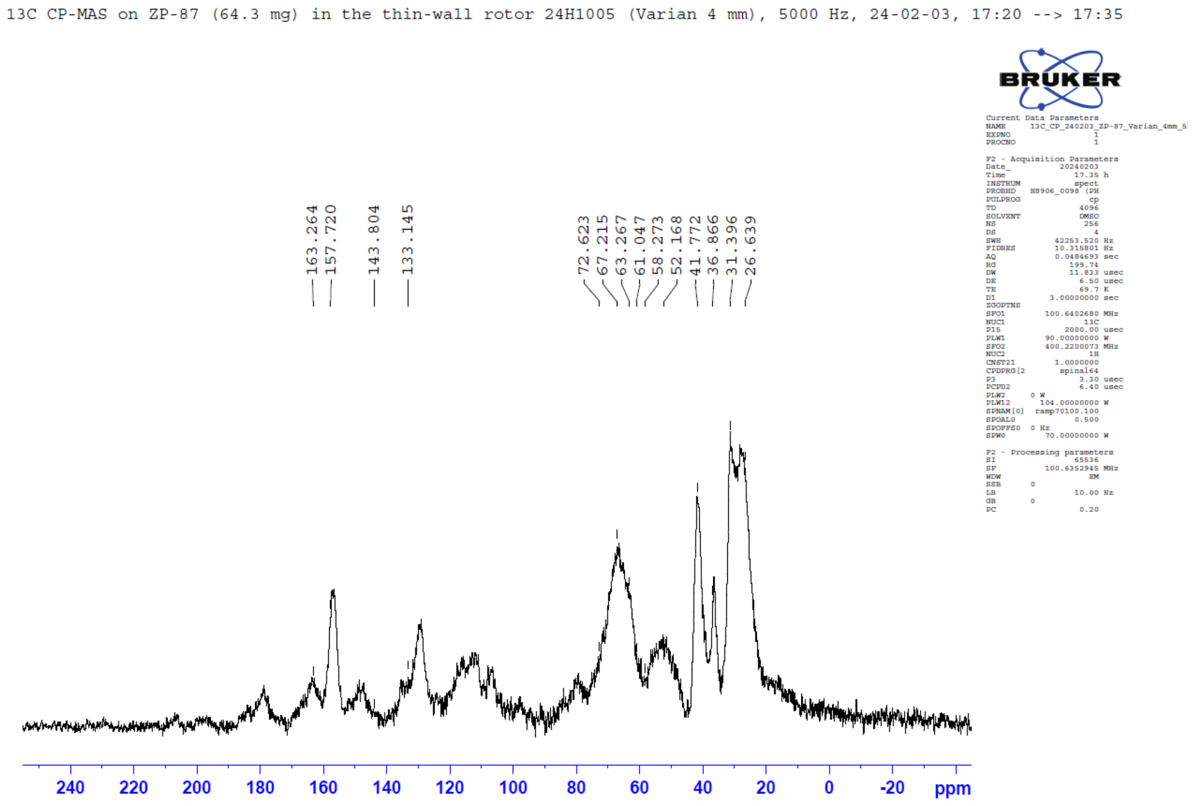

Figure S33. ^{13}C CP-MAS NMR (5 kHz) spectrum of **PHU$_2$-V**. Spinning sideband peaks are not assigned.

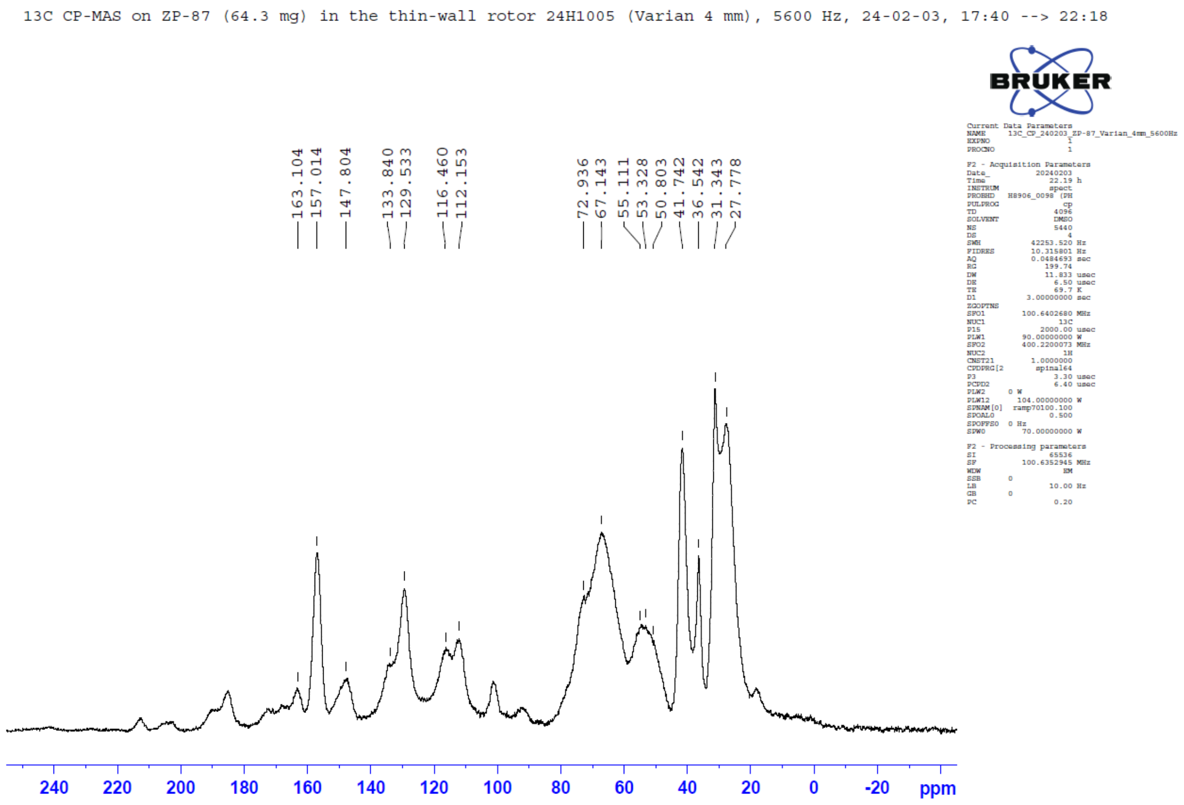

Figure S34. ^{13}C CP-MAS NMR (5.6 kHz) spectrum of **PHU$_2$-V**. Spinning sideband peaks are not assigned.

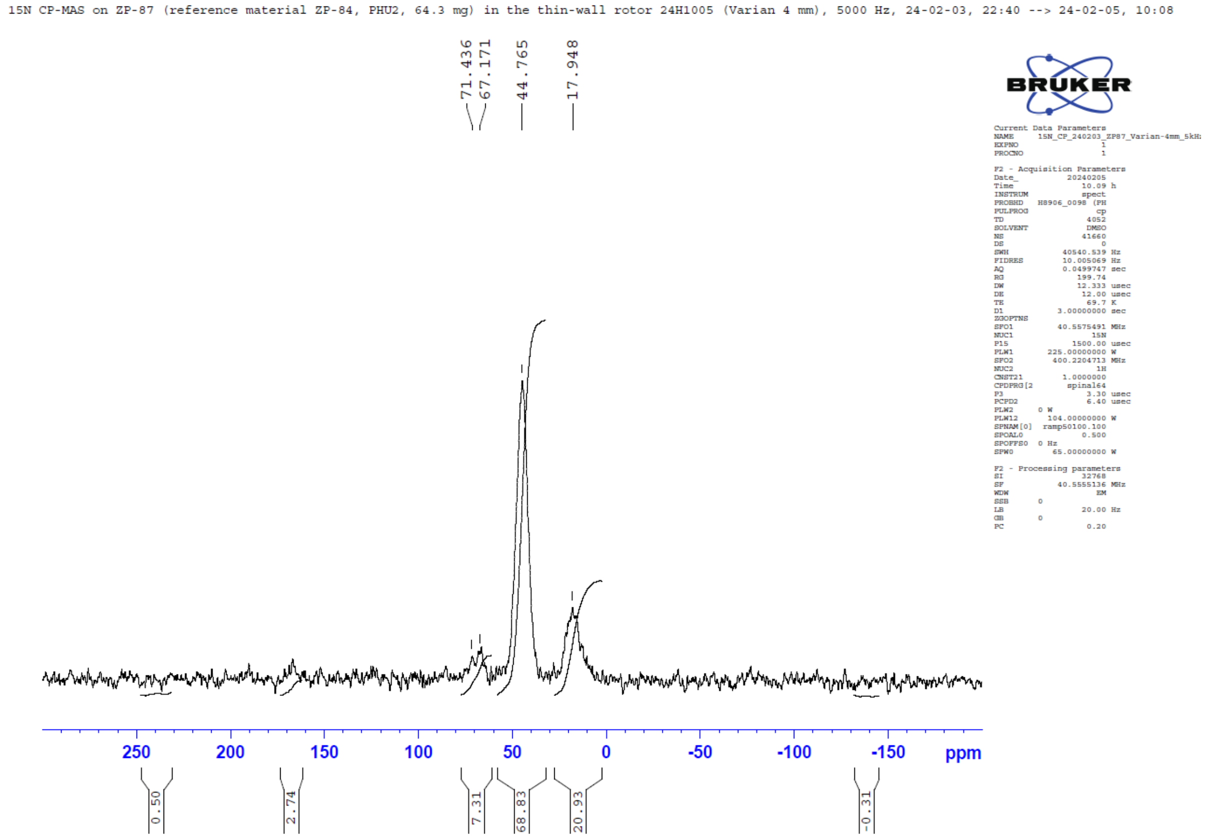

Figure S35. ^{15}N CP-MAS NMR (5 kHz) spectrum of **PHU$_2$-V**. The +1 spinning sideband peak is not assigned but is integrated. The baseline is also integrated at two intervals to estimate the signal-to-noise for different resonances.

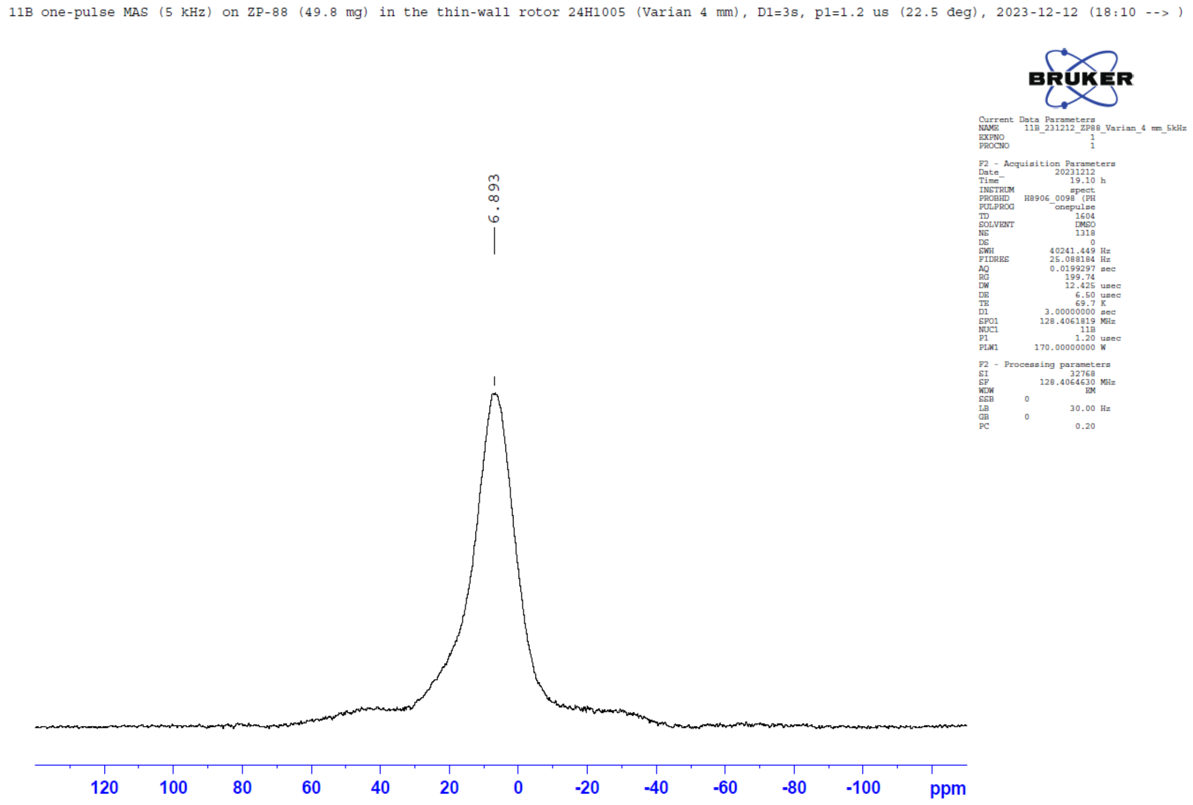

Figure S36. ^{11}B MAS NMR (5 kHz) spectrum of **PHU$_3$-V**.

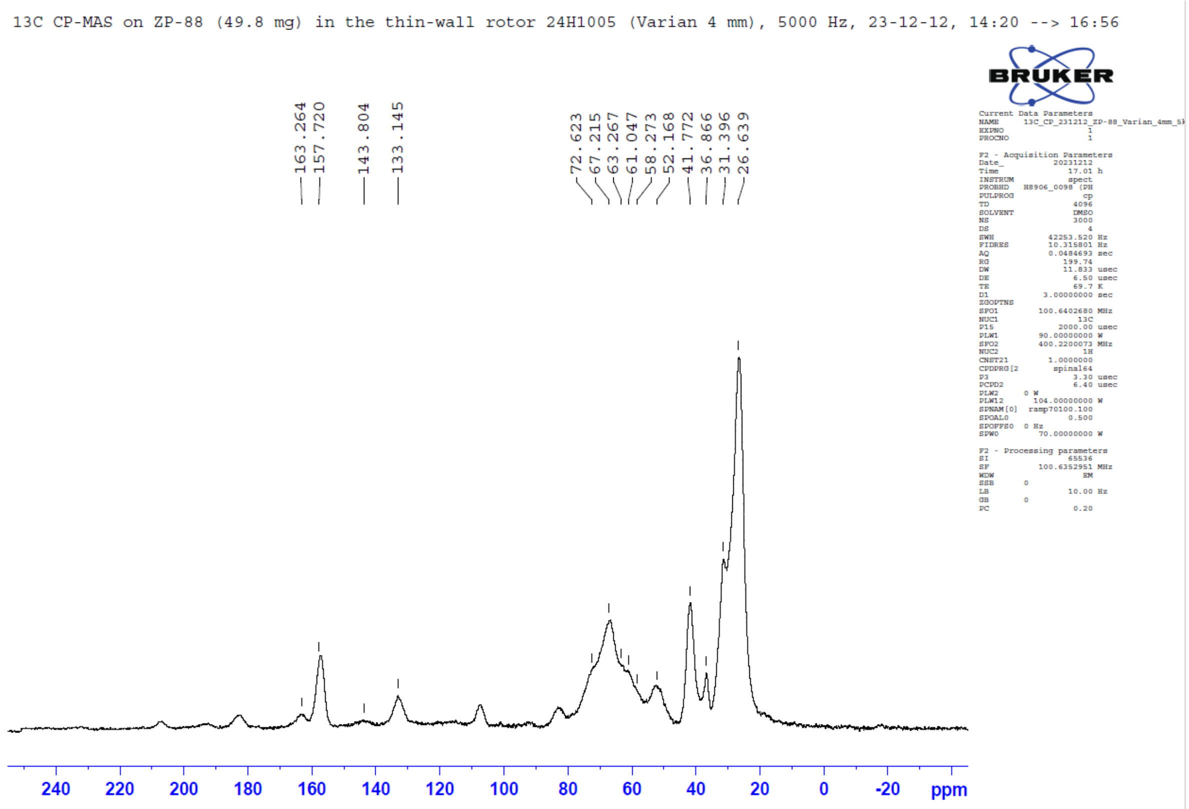

Figure S37. ^{13}C CP-MAS NMR (5 kHz) spectrum of **PHU$_3$-V**. Spinning sideband peaks are not assigned.

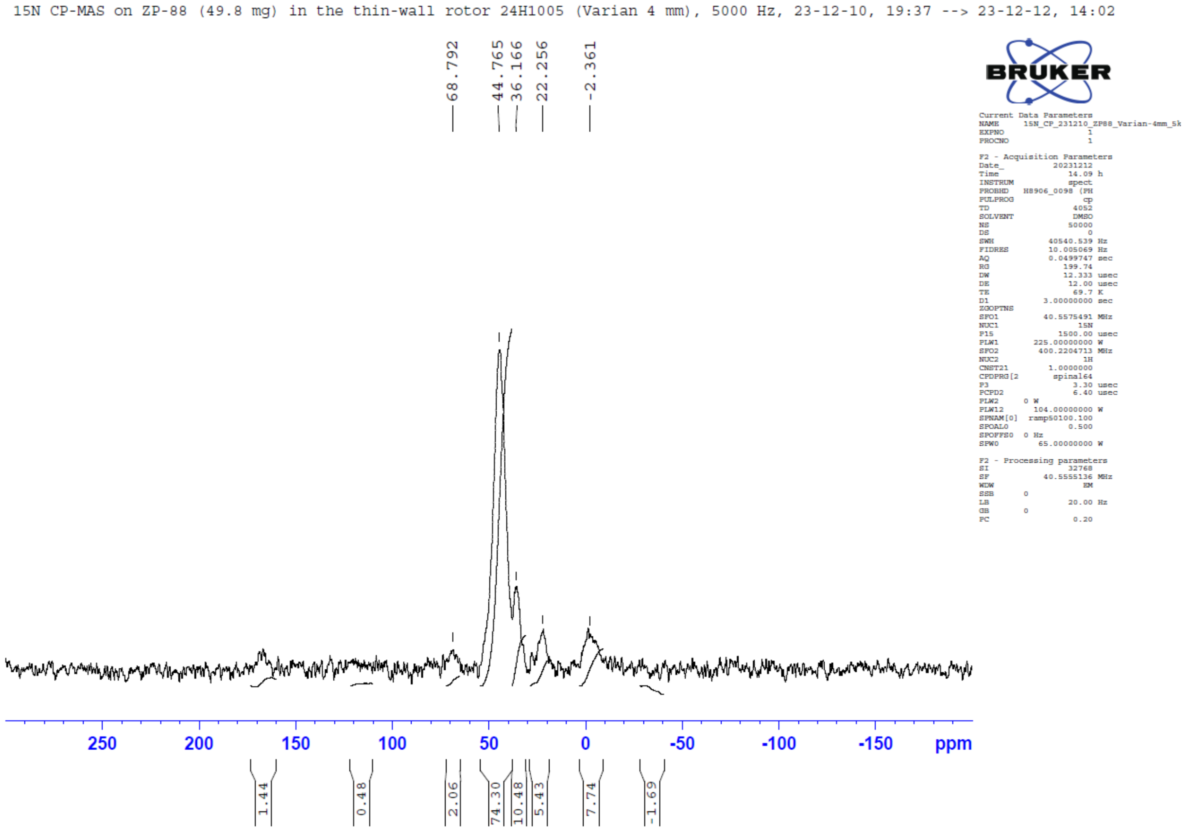

Figure S38. ^{15}N CP-MAS NMR (5 kHz) spectrum of **PHU$_3$-V**. The +1 spinning sideband peak is not assigned but is integrated. The baseline is also integrated at two intervals to estimate the signal-to-noise for different resonances.

VII. DSC plots of PHU polymers and vitrimers.

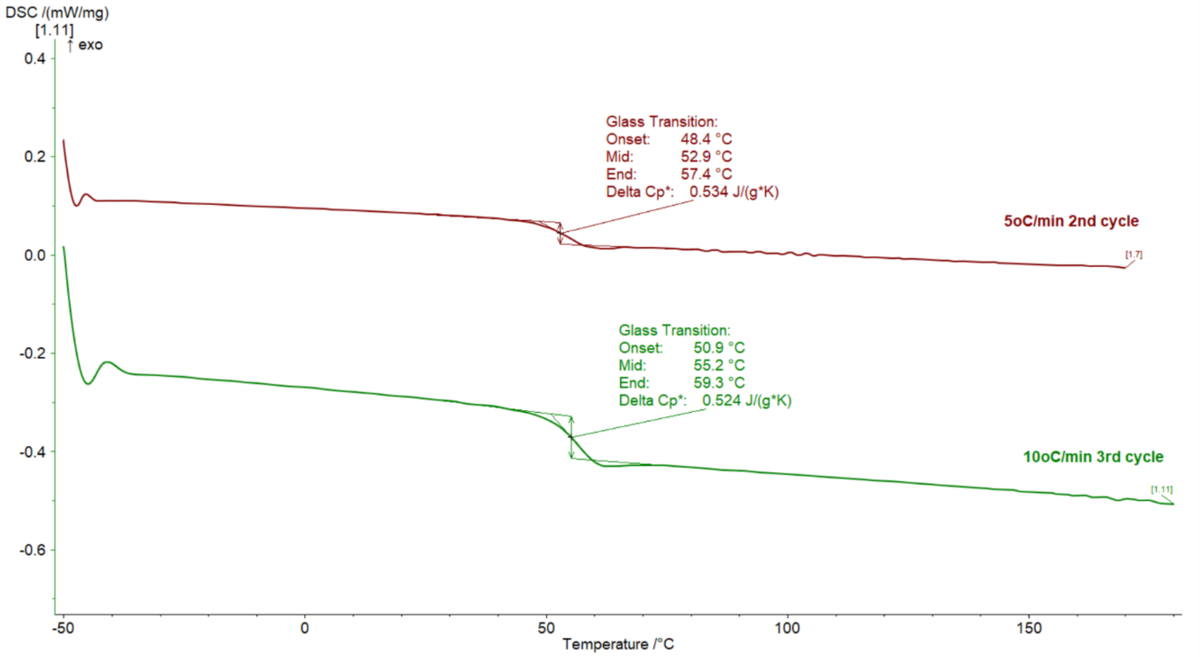

Figure S39. DSC plots of **PHU$_2$** at different heating rates.

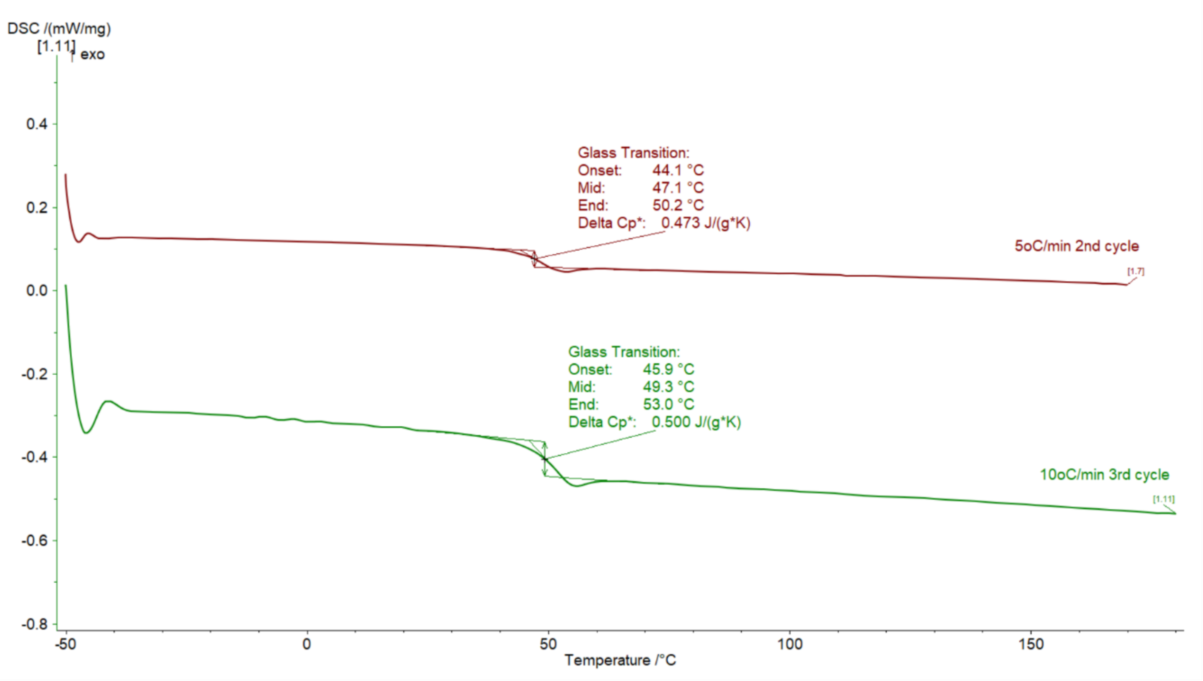

Figure S40. DSC plots of **PHU$_3$** at different heating rates.

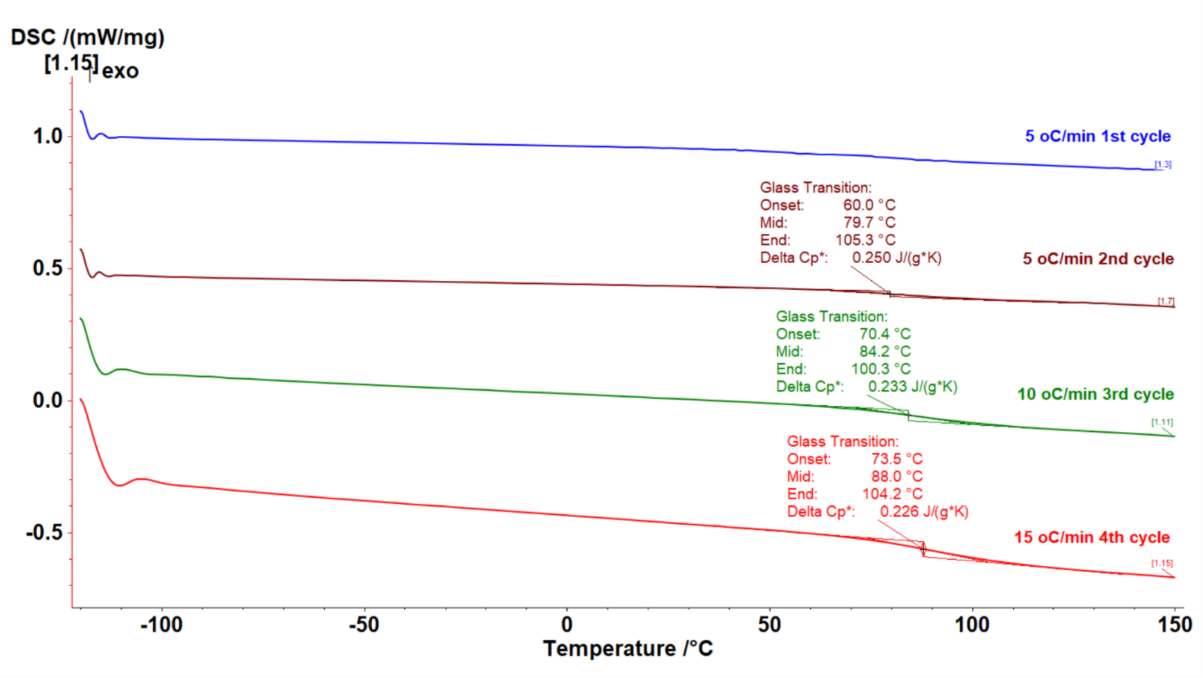

Figure S41. DSC plots of **PHU$_1$-V** at different heating rates.

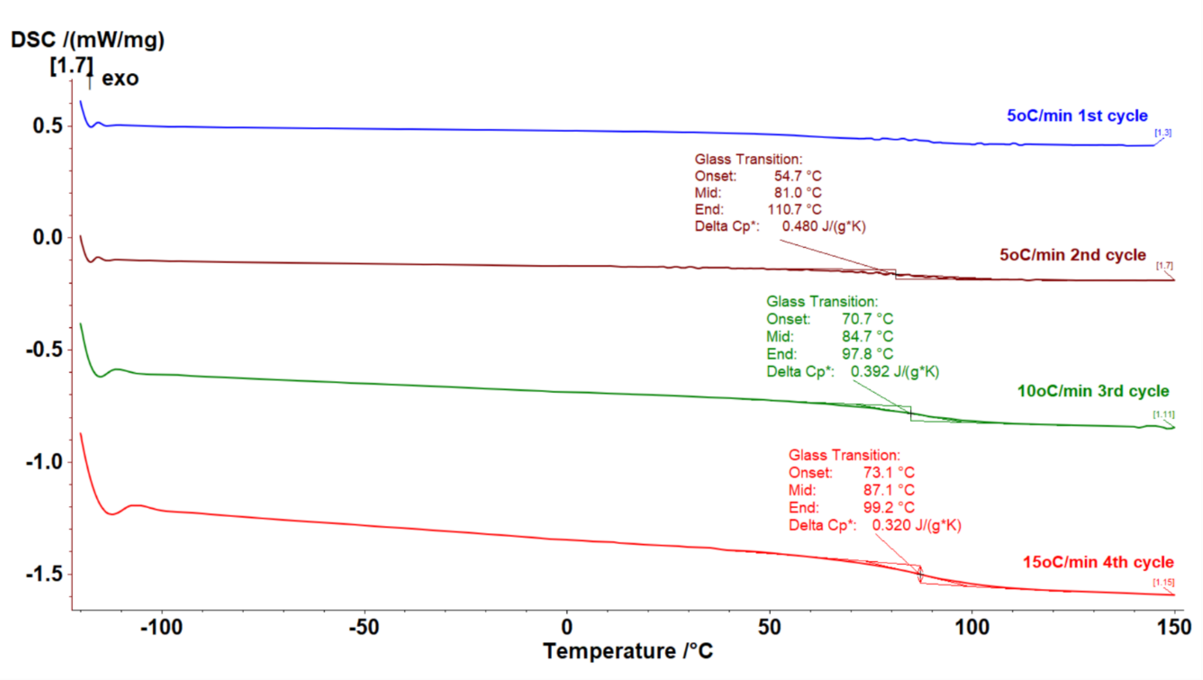

Figure S42. DSC plots of **PHU$_2$-V** at different heating rates.

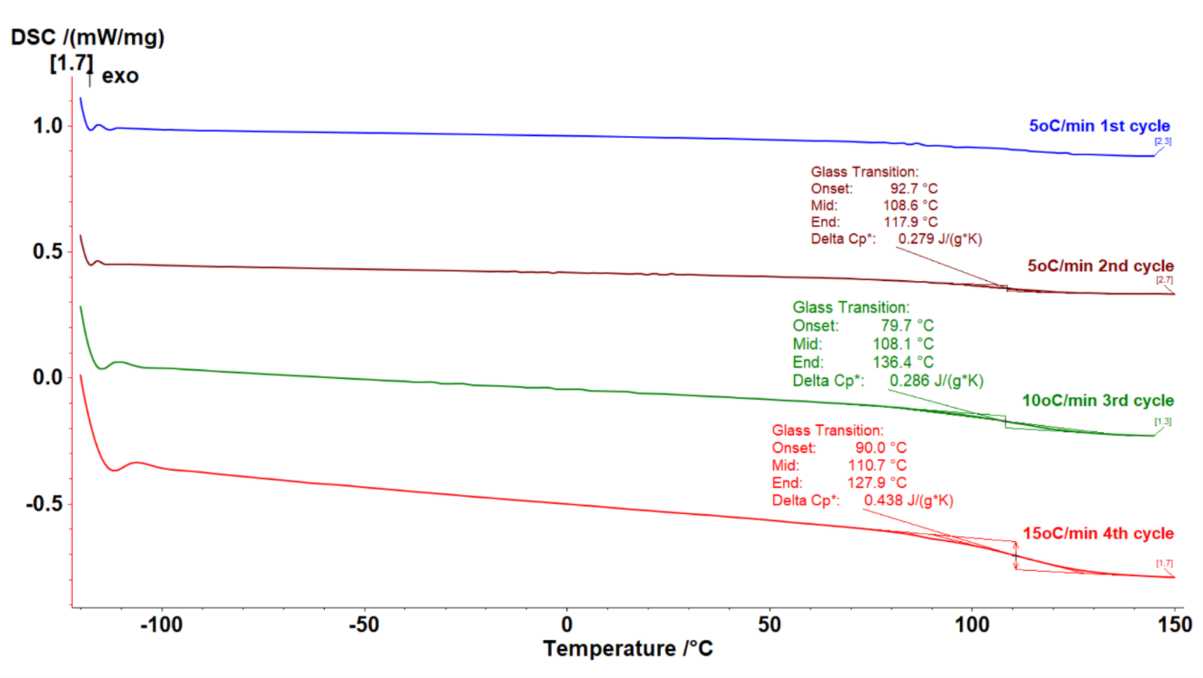

Figure S43. DSC plots of **PHU$_3$-V** at different heating rates.

VIII. Water absorption plots.

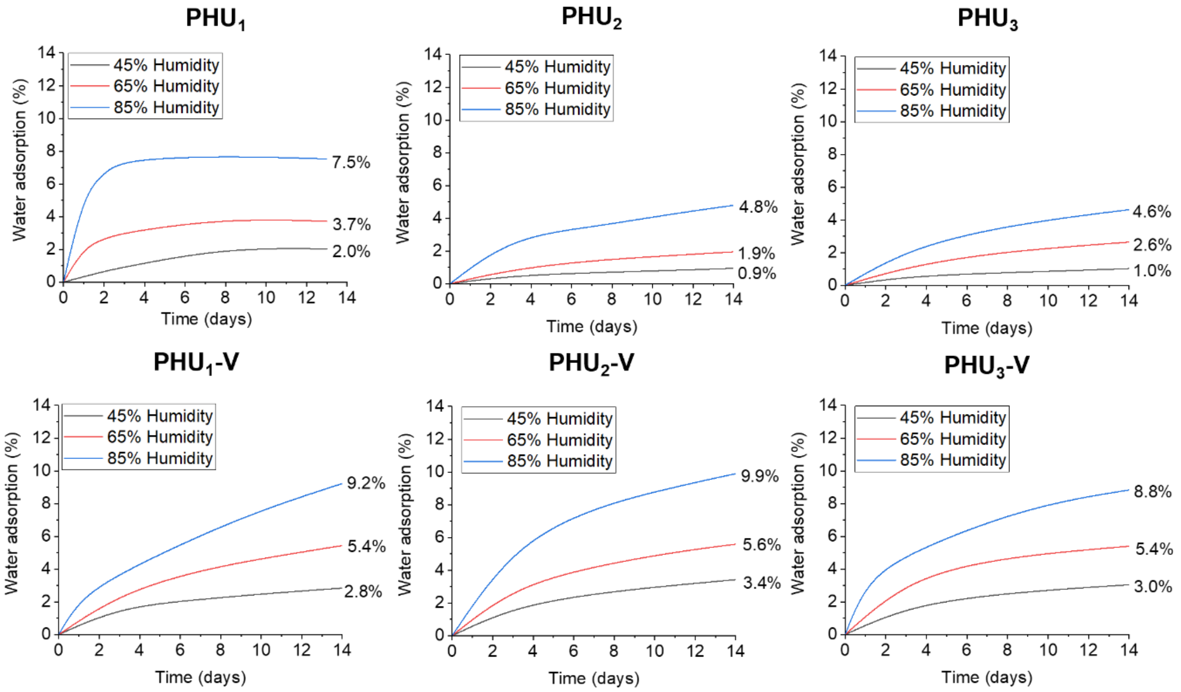

Figure S44. Water absorption plots for polymers **PHU$_1$ – PHU$_3$** and vitrimers **PHU$_1$-V – PHU$_3$-V** at 45, 65 and 85% relative humidity.

Table S1. Estimated T_α values for **PHU** polymers and **PHU-V** vitrimers as a function of water uptake.

Sample	***Dry***	***45% RH***		***65% RH***		***85% RH***	
	T_α (°C)[a]	Water (wt%)[b]	T_α (°C)[c]	Water (wt%)[b]	T_α (°C)[c]	Water (wt%)[b]	T_α (°C)[c]
**PHU$_1$**	69	2.0%	59	3.7%	51	7.5%	34
**PHU$_2$**	68	0.9%	63	1.9%	58	4.8%	45
**PHU$_3$**	63	1.0%	58	2.6%	51	4.6%	42
**PHU$_1$-V**	130	2.8%	109	5.4%	91	9.2%	68
**PHU$_2$-V**	121	3.4%	97	5.6%	83	9.9%	59
**PHU$_3$-V**	167	3.0%	139	5.4%	120	8.8%	95

[a] T_α (taken as peak maximum from the loss modulus (*E''*) curve) measured by DMTA at a heating rate of 5 °C/min; [b] Determined after 14 days of conditioning at selected humidity levels and 22°C; [c] Calculated using the Fox equation[8]: $\frac{1}{T_\alpha} = \frac{w_1}{T_{\alpha 1}} + \frac{w_2}{T_{\alpha 2}}$, where in this case $T_{\alpha 1}$, $T_{\alpha 2}$, w_1 and w_2 are the main relaxation temperatures and weight fractions of polymer and water, respectively, and where T_α for water is taken as 136 K.[9]

[8] Fox, T. G. Influence of Diluent and of Copolymer Composition on the Glass Temperature of a Polymer System. *Bull. Am. Phys. Soc.* **1956**, *1*, 123.

[9] Capaccioli, S.; Ngai, K. L. Resolving the Controversy on the Glass Transition Temperature of Water? *J. Chem. Phys.* **2011**, *135* (10). https://doi.org/10.1063/1.3633242.

IX. SEM of milled **PHU$_1$-V**, **PHU$_2$-V** and **PHU$_3$-V**.

Table S2. Average particle size distribution for ball milled PHU-V samples[a].

Sample	Average particle size, µm		
	Large fraction	Medium fraction	Small fraction
**PHU$_1$-V**	712 ± 84	156 ± 21	51 ± 14
**PHU$_2$-V**	148 ± 22	67 ± 9	25 ± 6
**PHU$_3$-V**	378 ± 62	102 ± 18	32 ± 7

[a] Calculated using SEM images, at least 10 particles were measured from 4 independent pictures for each fraction.

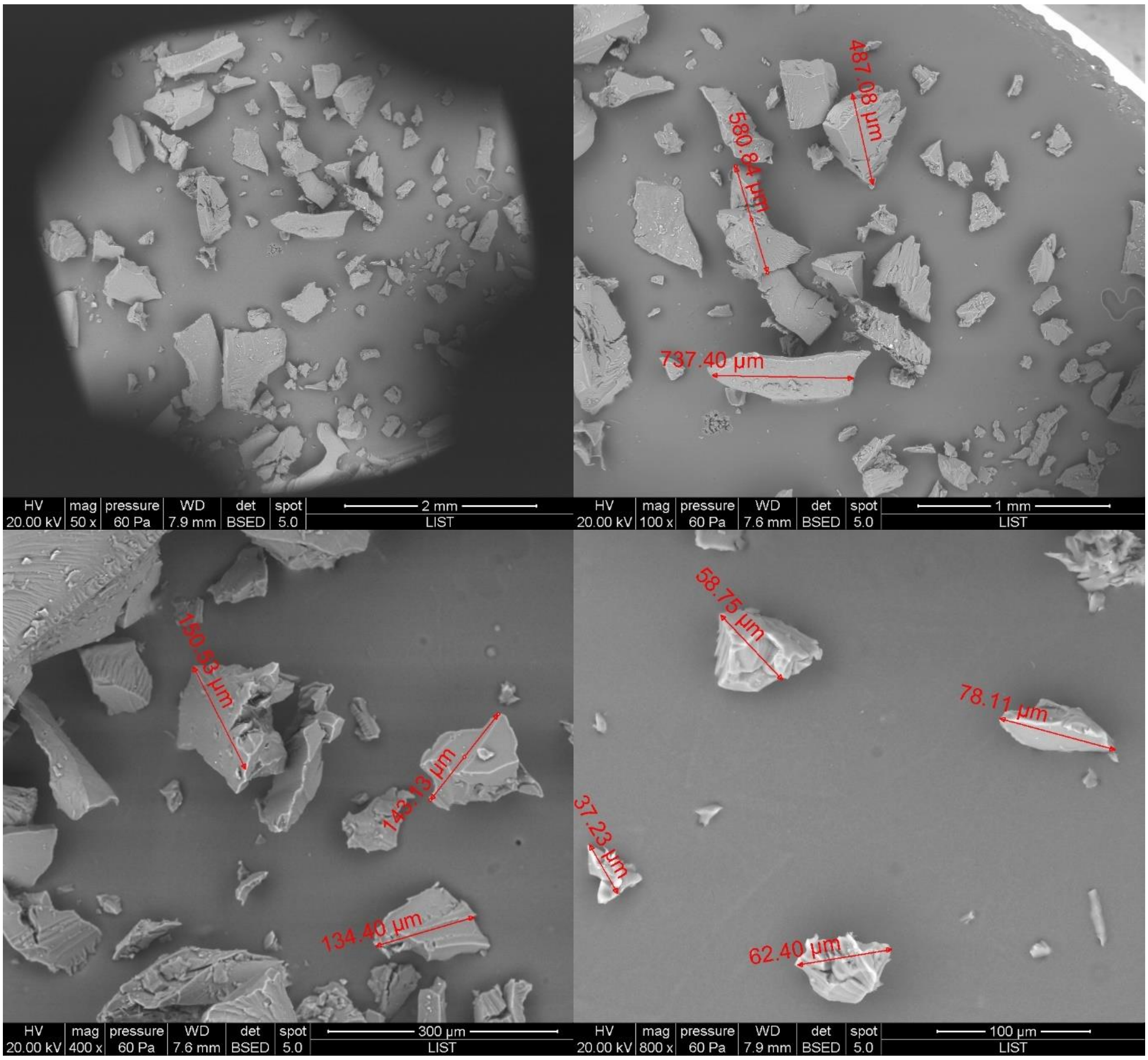

Figure S45. SEM images of milled PHU$_1$-V.

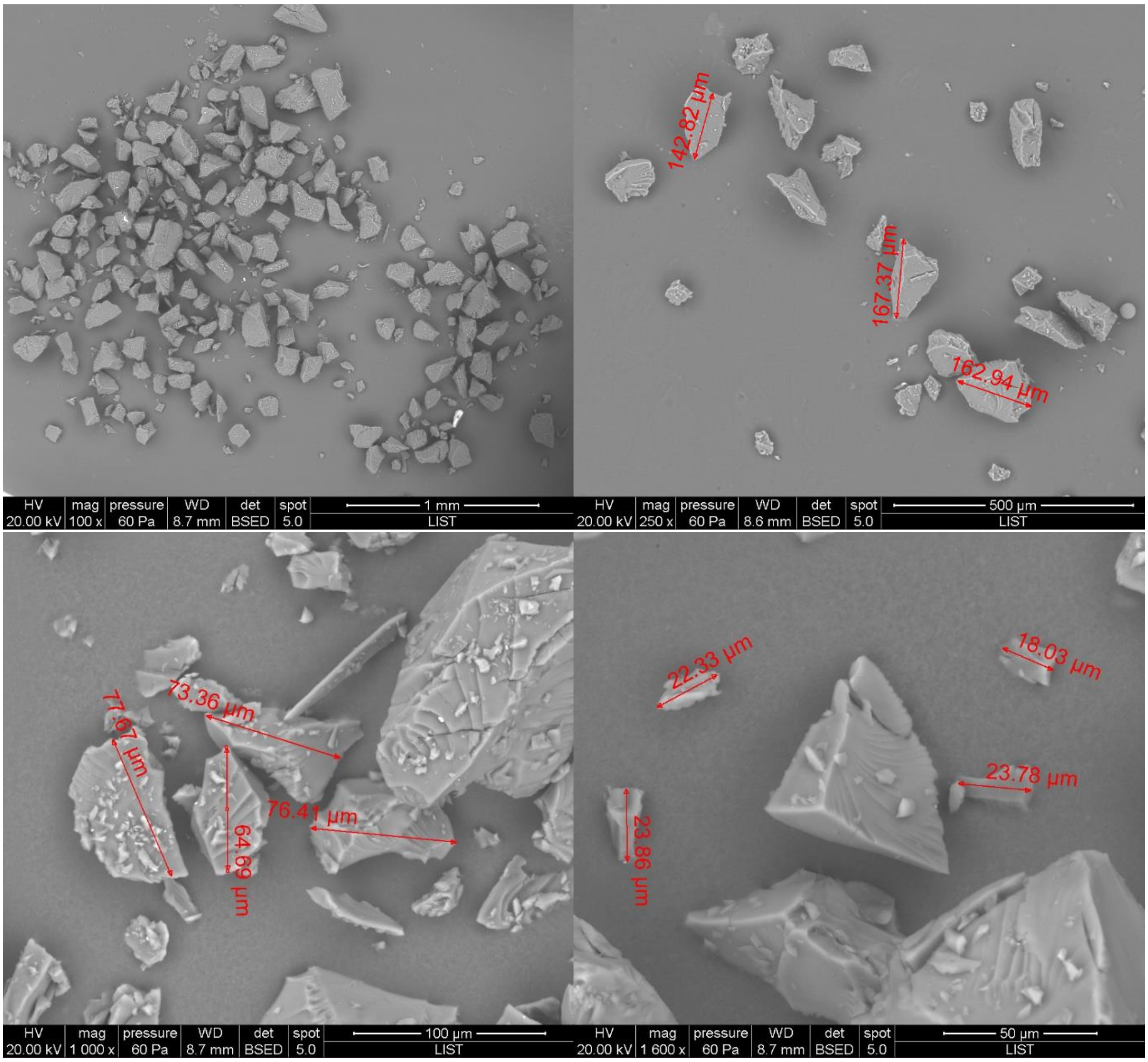

Figure S46. SEM images of milled PHU_2-V.

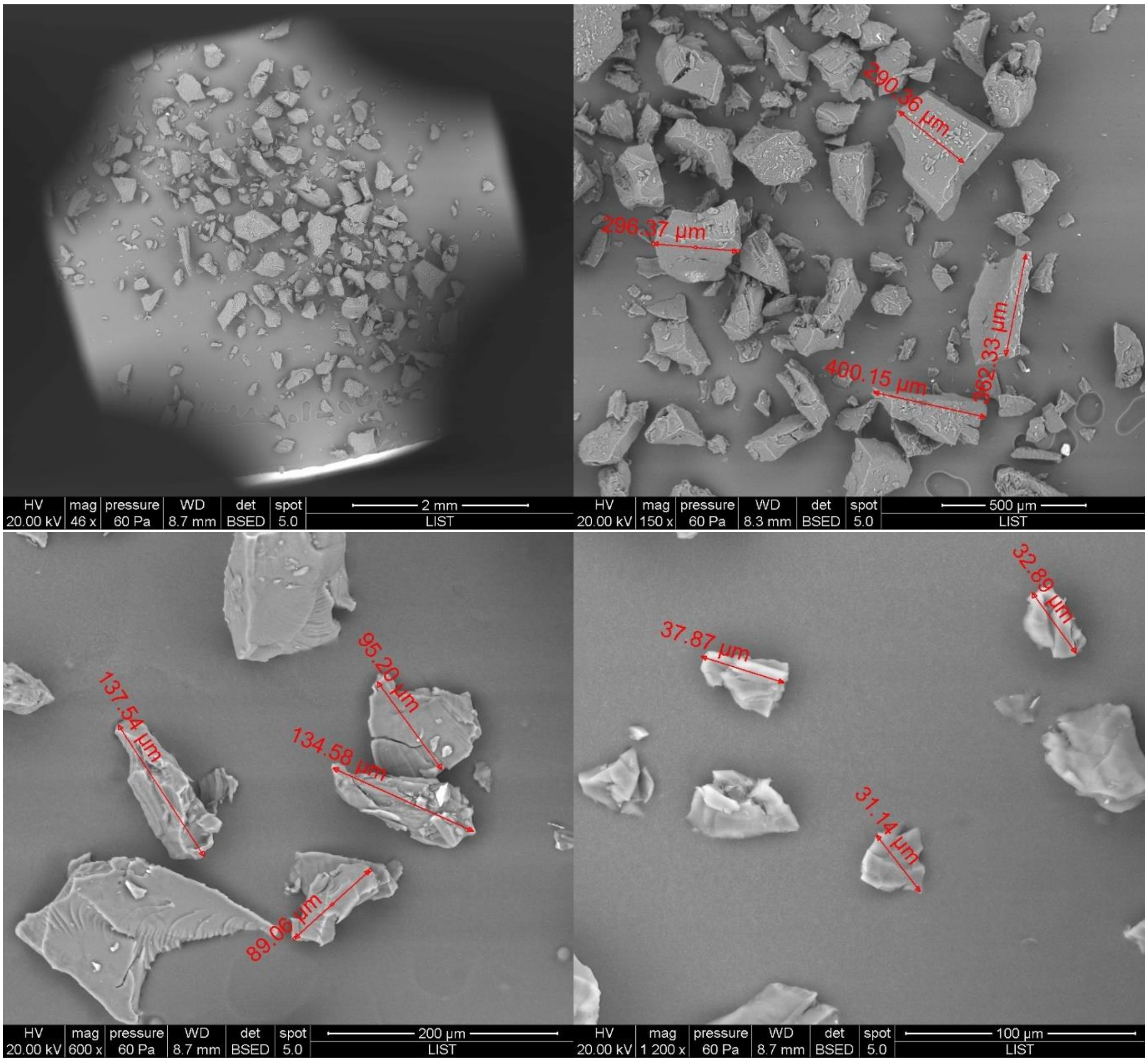

Figure S47. SEM images of milled PHU_3-V.

X. DMTA plots.

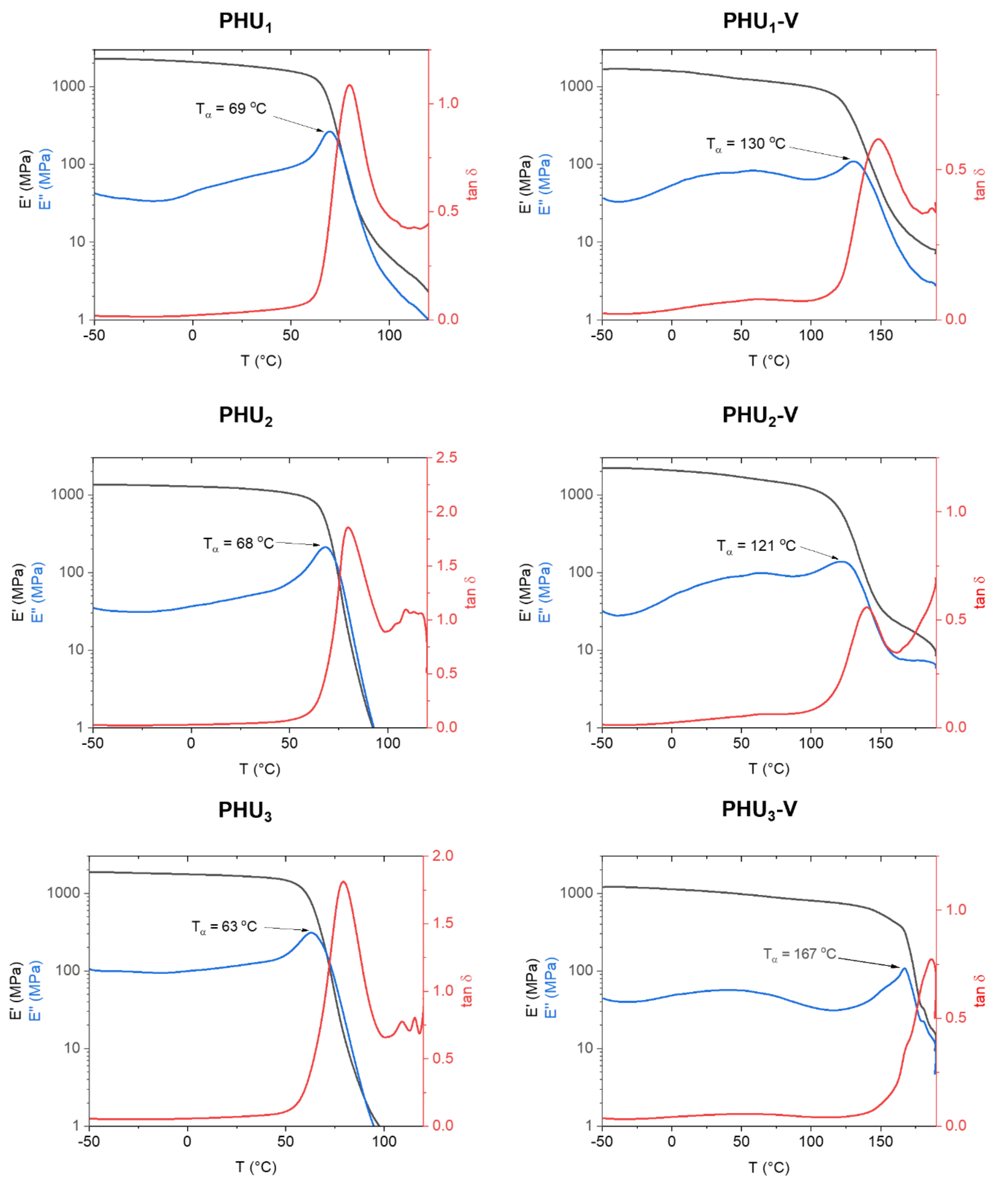

Figure S48. DMTA storage moduli (*E'*), loss moduli (*E''*) and tan *δ* plots for **PHU_1, PHU_2** and **PHU_3** polymers and **PHU_1-V**, **PHU_2-V** and **PHU_3-V** vitrimers.

XI. Solvent resistance and swelling.

Table S3. Swelling and gel content of PHU_3-V in different solvents[a].

Solvent	Swelling (wt%)[b]	Gel content (wt%)[c]
Dichloromethane	76	97
Cyclohexane	2	>99
Methanol	86	85
Dimethyl sulfoxide	67	>99
Toluene	7	99
Water	69	90

[a] Swelling tests performed at 25°C for 30 days; swelling and gel content were measured gravimetrically using samples swelled for 30 days, then dried in vacuum at 100 °C for 24 hours; [b] Swelling was determined using equation S2; [c] Gel content was determined using equation S3.

XII. Mechanical recycling.

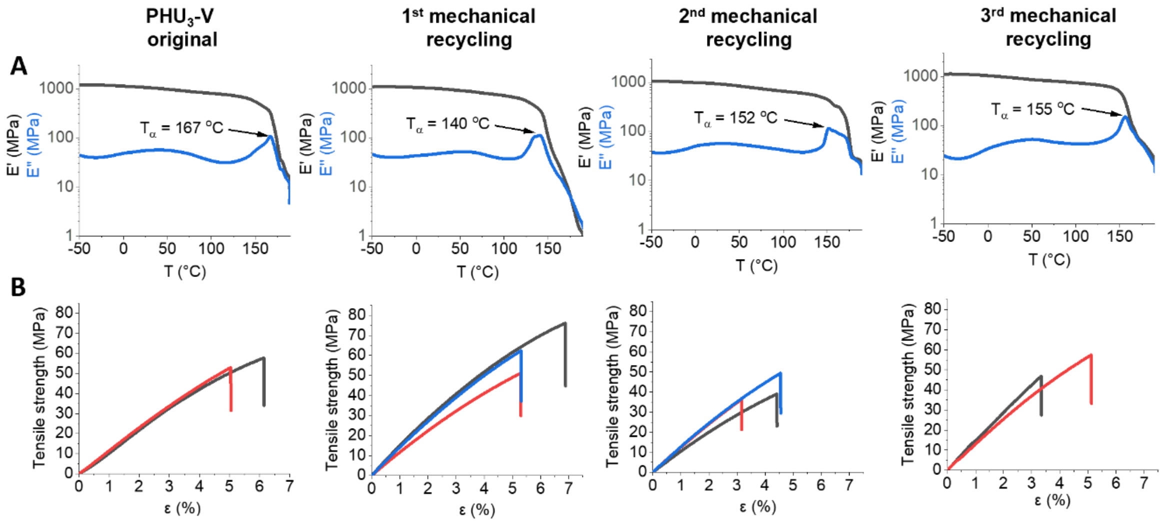

Figure S49. DMTA storage (*E'*) and loss (*E''*) moduli (A) and tensile stress-strain plots (B) for original **PHU$_3$-V** (leftmost column) and **PHU$_3$-V** after one, two or three mechanical recycles.

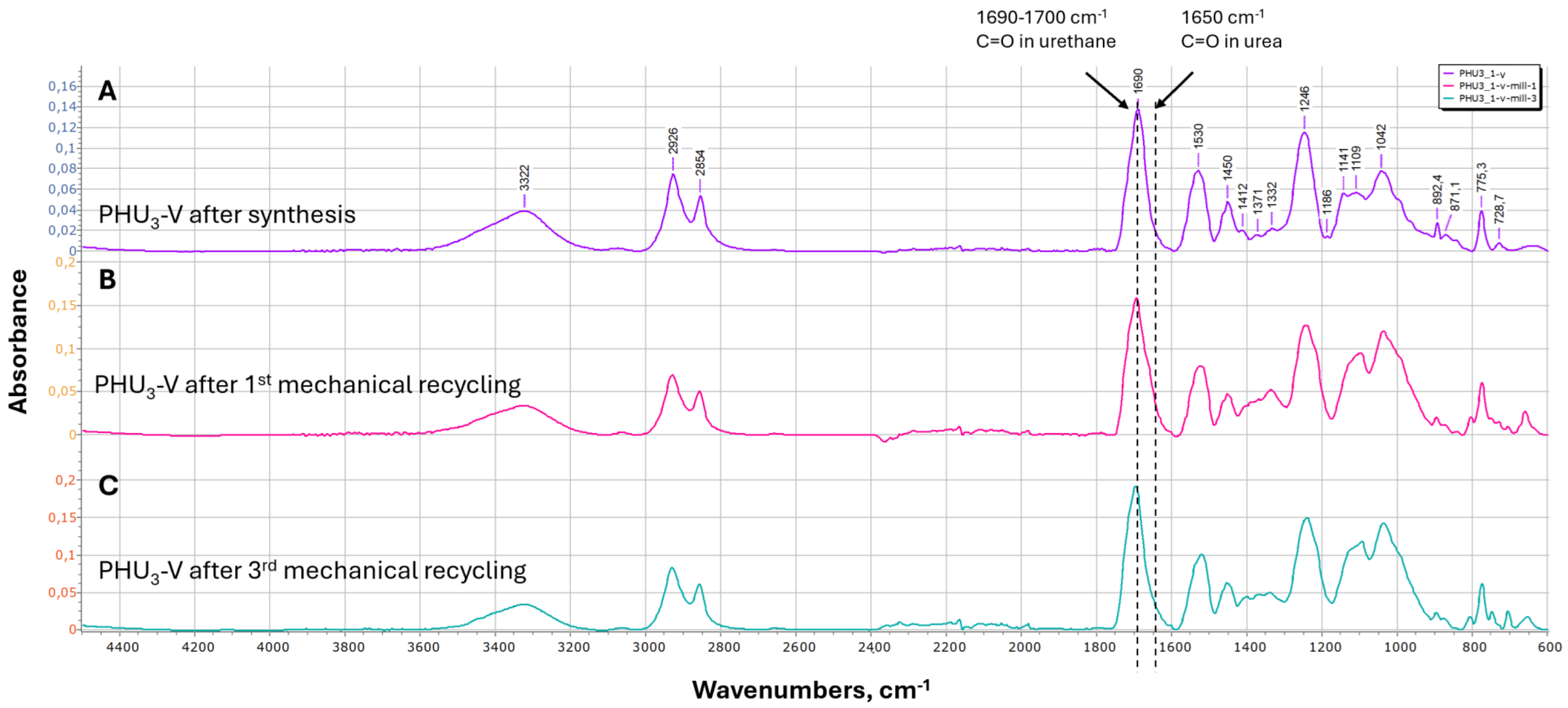

Figure S50. FTIR spectra of the **PHU$_3$-V** after synthesis, (A) 1st mechanical recycling (B) and 3rd mechanical recycling (C) (milling).

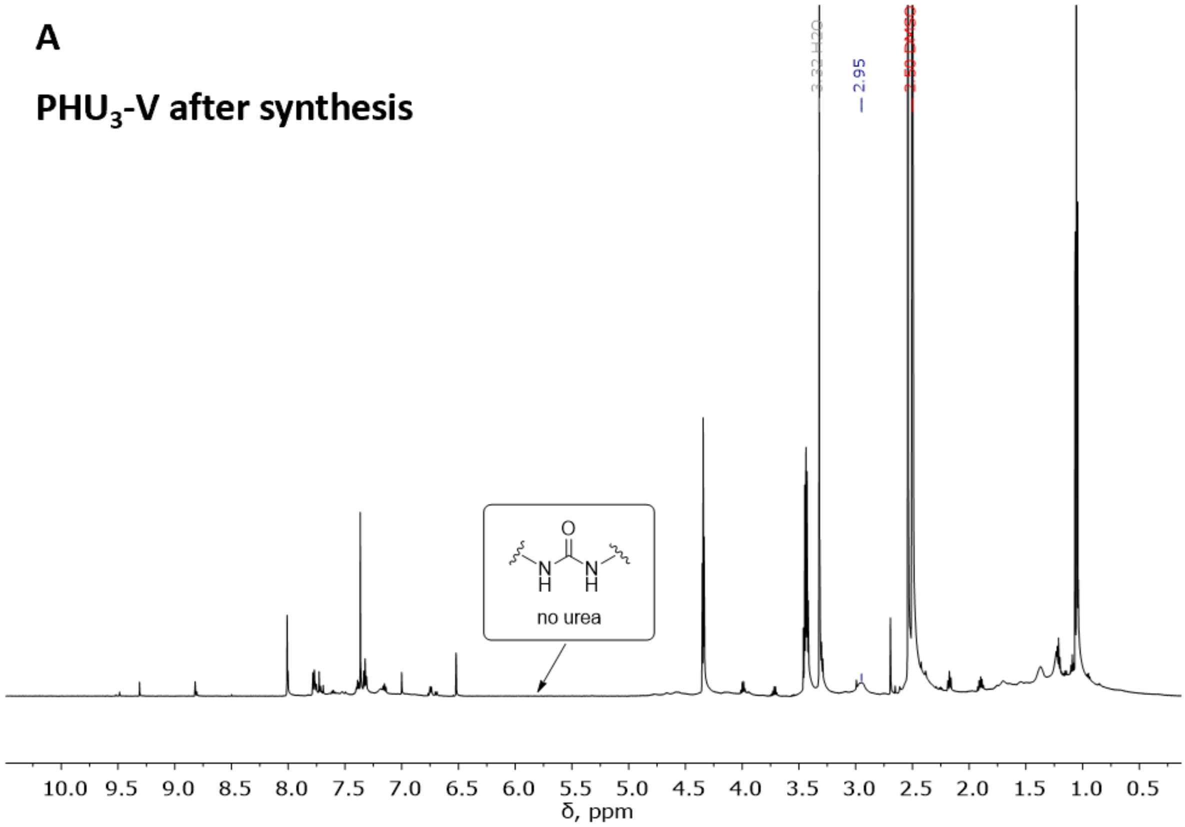
A
PHU3-V after synthesis
3.33 H2O
2.95
2.50 DMSO
no urea
10.0 9.5 9.0 8.5 8.0 7.5 7.0 6.5 6.0 5.5 5.0 4.5 4.0 3.5 3.0 2.5 2.0 1.5 1.0 0.5
δ, ppm

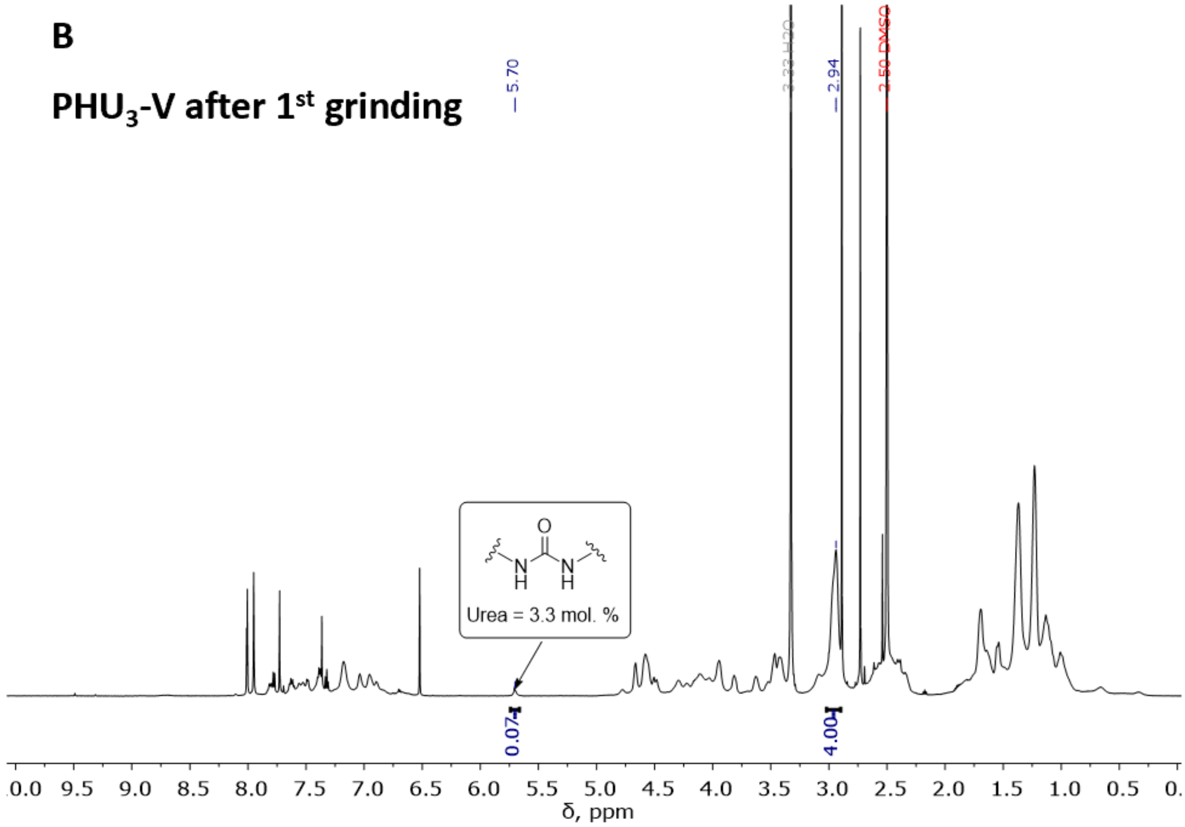
B
PHU3-V after 1st grinding
5.70
3.33 H2O
2.94
2.50 DMSO
Urea = 3.3 mol. %
0.07
4.00
0.0 9.5 9.0 8.5 8.0 7.5 7.0 6.5 6.0 5.5 5.0 4.5 4.0 3.5 3.0 2.5 2.0 1.5 1.0 0.5 0.
δ, ppm

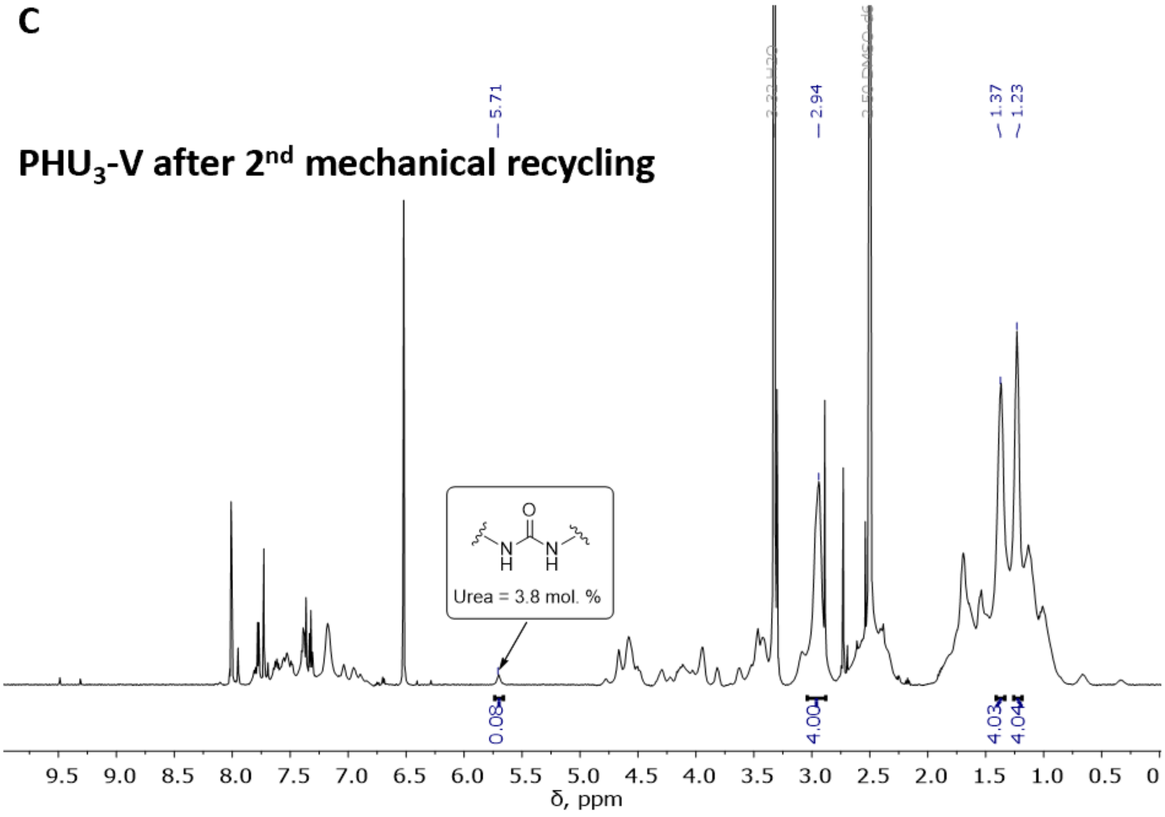

Figure S51. ^{1}H NMR spectra of **PHU$_3$-V** after synthesis (A), first grinding (B) and two subsequent mechanical recycles (C) in DMSO-d$_6$ (in a gel/swelled form).

XIII. Chemical recycling.

General method

PHU$_3$-V bars and dumbbells were broken into pieces and used for chemical recycling. Approximately 1.0 g of **PHU$_3$-V** was placed in a 250 mL round-bottom flask filled with 60 mL of ethanol and 40 mL of 0.1 M NaOH (aq.). The flask was fitted with a condenser, and the mixture was stirred at 70°C overnight (~12 hours). Under these conditions, the **PHU$_3$-V** vitrimer dissolved in the reaction medium. Afterwards, the flask was cooled to room temperature and the solvent was removed by rotary evaporation (40 °C, 175 mbar). As the ethanol fraction evaporated, the de-crosslinked polymer precipitated and was collected repeatedly. The isolated polymer was then washed three times with 100 mL of deionized water before being dissolved in DMSO. The recovered **PHU$_3$** was reprecipitated from DMSO into water, then separated by centrifugation and filtration. The resulting paste was dried under dynamic vacuum (~0.5 mbar) at 100 °C for 16 hours, yielding the recycled **PHU$_3$** polymer as a powder.

Table S4. Optimization of conditions for chemical recycling[a].

Entry	Solvent/reagent	V, mL	Acid/base	Quantity	Recycled **PHU$_3$** polymer			
					Yield, g	Yield, %	M_n (g·mol^{-1})	M_w/M_n
1[b]	THF/2,3-butanediol	40/60	-	-	Material remained intact			
2	EtOH	60	-	-	Material remained intact			
3	EtOH	60	Acetic acid + water	2 mL + 20 mL	Not fully soluble			
4	EtOH	60	0.1 M aq. HCl	40 mL	Not fully soluble			
5	EtOH	60	TBD (5 mol. %)	12.7 mg	Material remained intact			
6	EtOH	60	0.1 M aq. NaOH	40 mL	0.6	60%	15300[c]	4.2[c]
7	EtOH	60	0.1 M aq. KOH	40 mL	Material disintegrated into small particles, but was insoluble in DMSO after isolation			
8	EtOH	60	0.1 M aq. NH_3	40 mL	Material remained intact			

[a] Conditions of recycling as per general method: 70 °C, 12 h; [b] adopted from the literature;[10]
[c] original **PHU$_3$** polymer before cross-linking: M_n = 16900 g·mol^{-1}; M_w/M_n = 2.5.

[10] Anderson, L.; Sanders, E. W.; Unthank, M. G. Recyclable Thermosets Based on Modified Epoxy-Amine Network Polymers. *Mater. Horizons* **2023**, *10* (3), 889–898. https://doi.org/10.1039/D2MH01211A

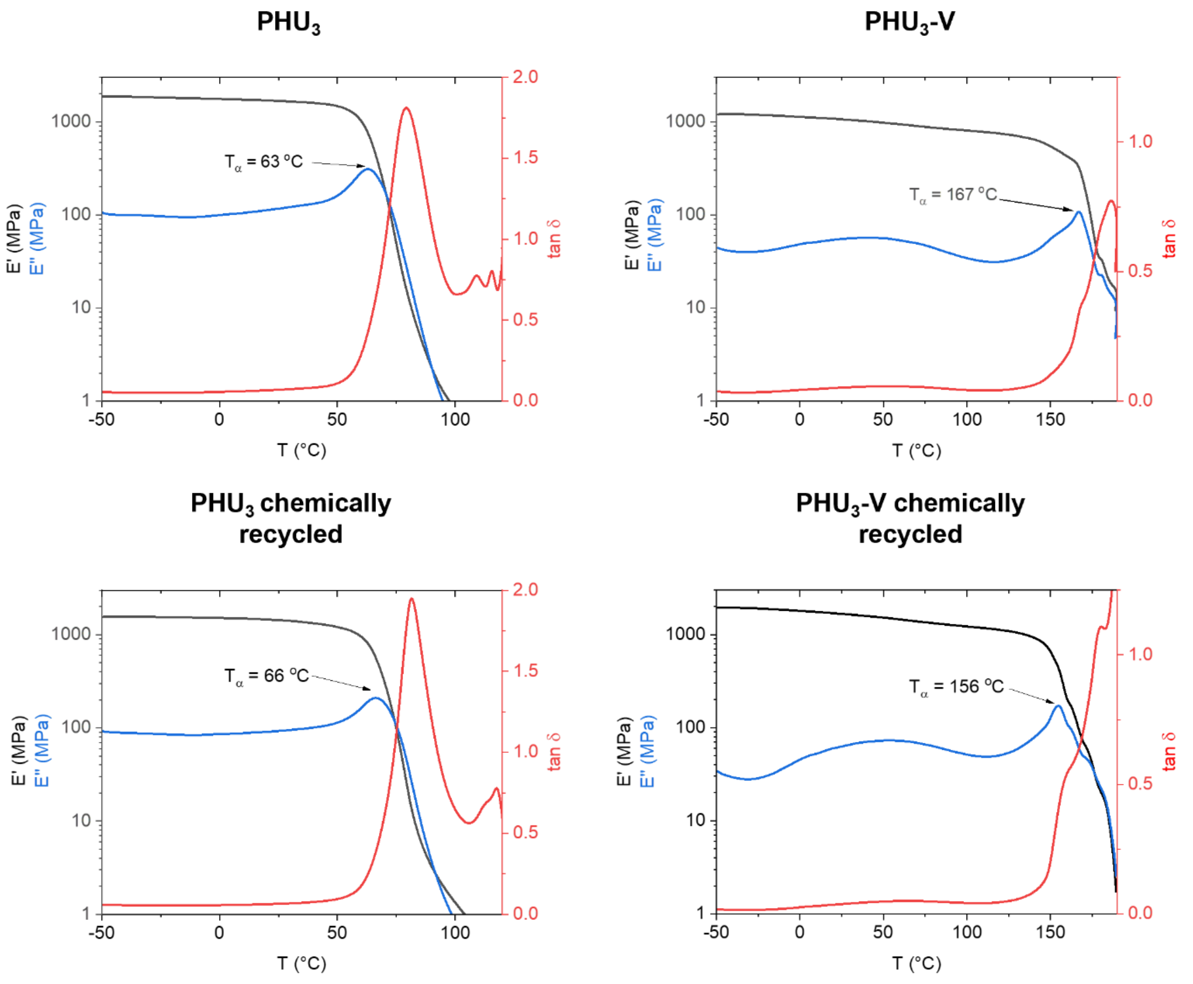

Figure S52. DMTA storage moduli (E'), loss moduli (E'') and tan δ plots for **PHU₃** polymer, **PHU₃-V** vitrimer and their chemically recycled counterparts.

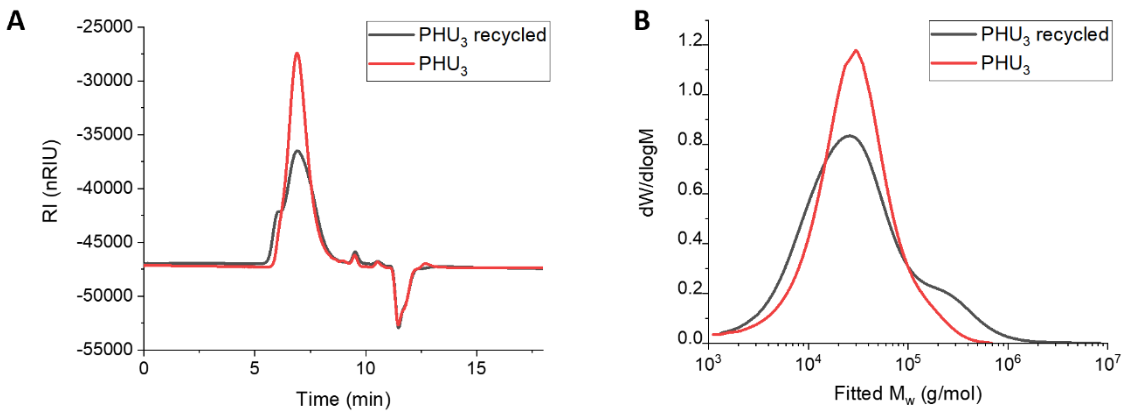

Figure S53. Raw (A) and fitted (B) GPC plots for original and recycled **PHU₃**.

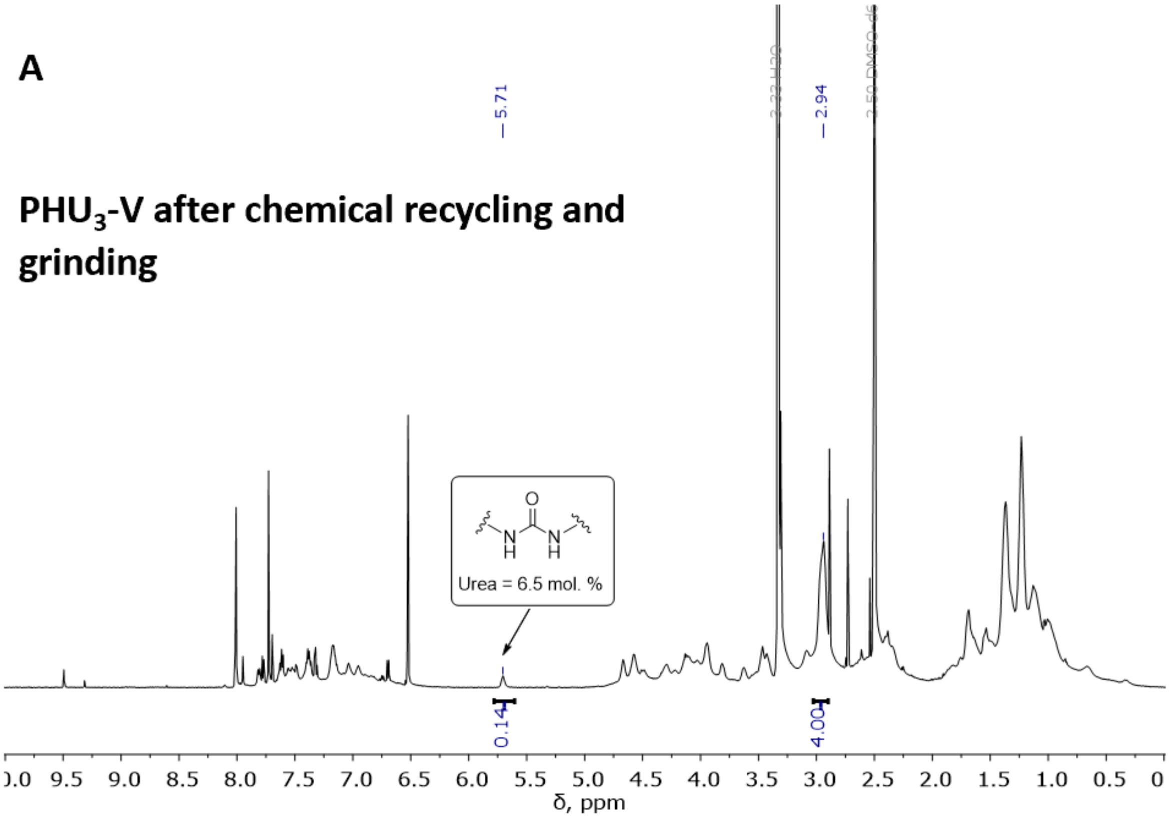

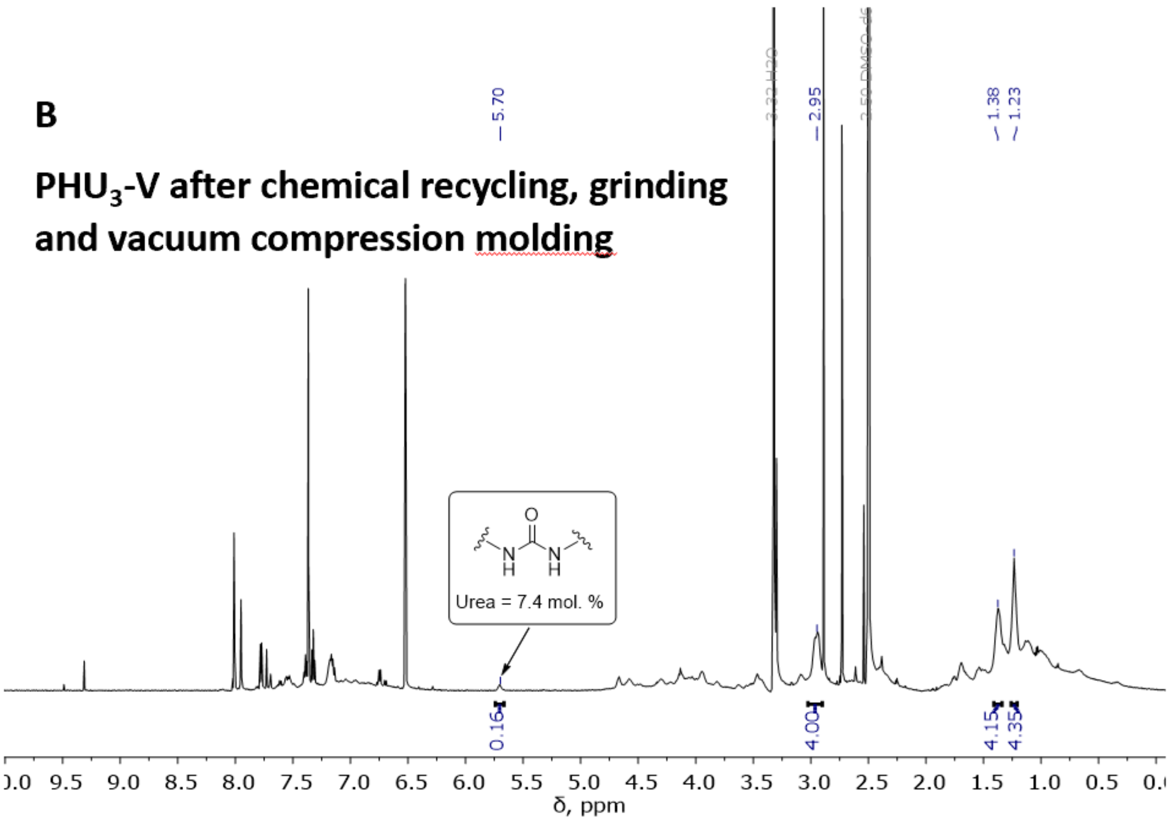

Figure S54. ^{1}H NMR spectra of **PHU3-V** after chemical recycling and grinding (A) and after subsequent vacuum compression molding (B) in DMSO-d_6 (in a gel/swelled form).

XIV. Evaluation of PHU_3-V hydrolytic stability in boiling water.

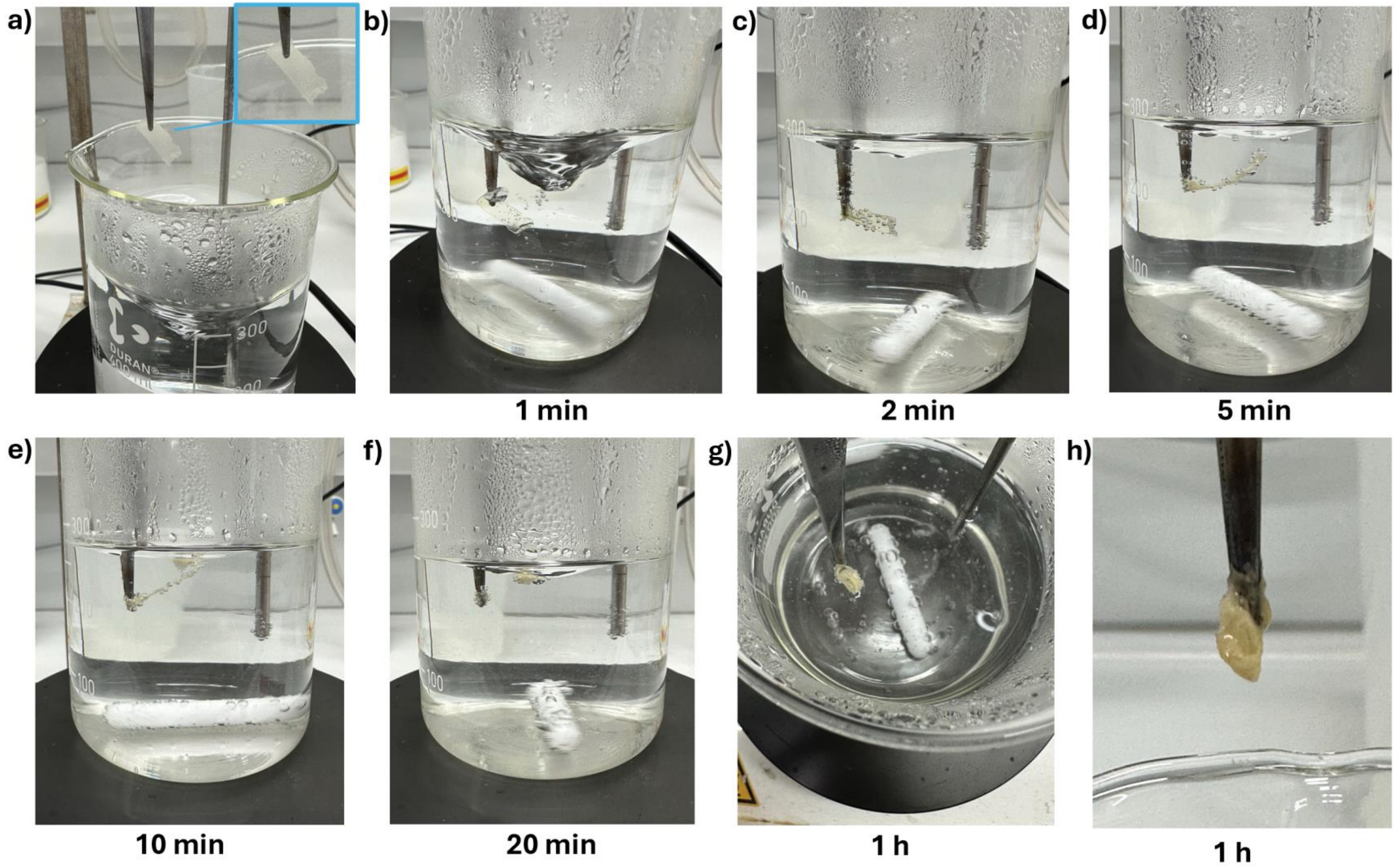

Figure S55. Appearance of the film prepared from **PHU_3-V** polymer before (A) and after boiling in water for 1 minute (B), 2 minutes (C), 5 minutes (D), 10 minutes (E), 20 minutes (F) and 1 hour (G and H).

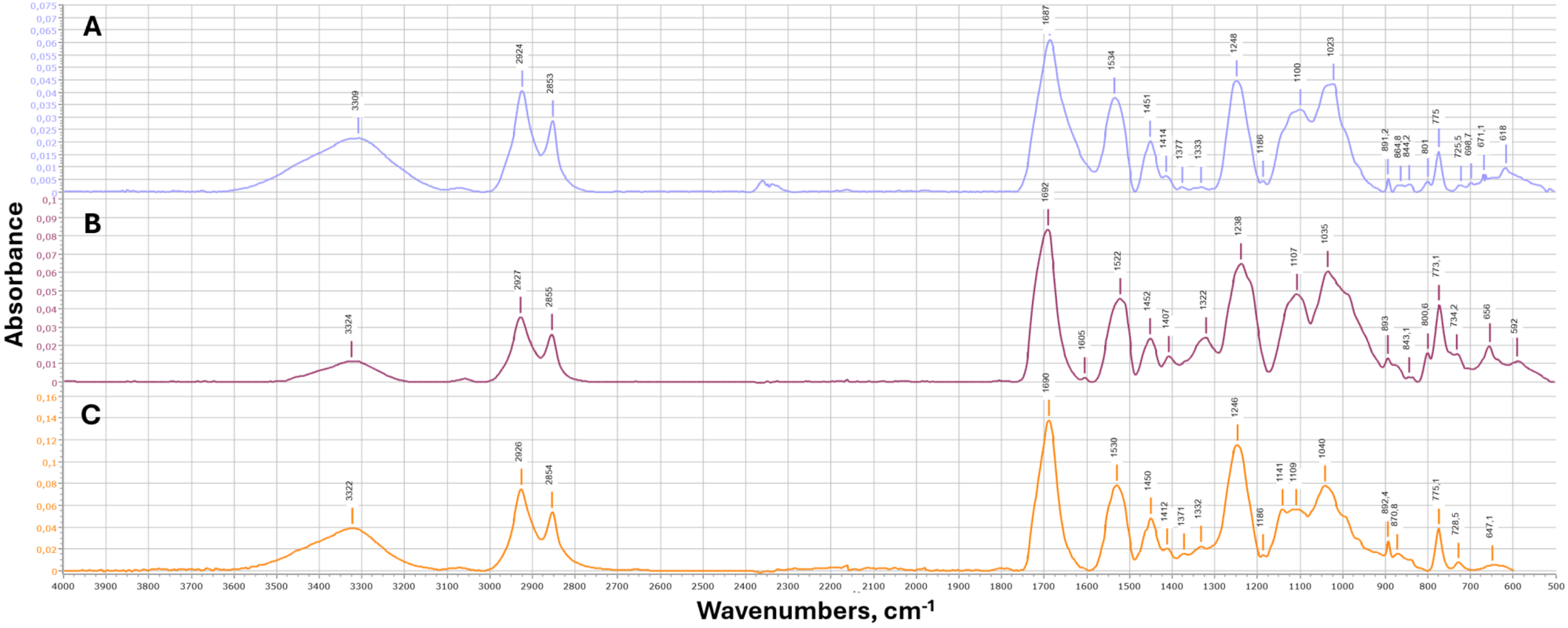

Figure S56. FTIR spectra of the **PHU$_3$-V** film boiled in water for 1 hour (A), **PHU$_3$-V** neat film (B) and **PHU$_3$-V** milled powder (C).

XV. Comparison of thermo-mechanical properties and reprocessability of PHU vitrimers

Table S5. Comparison of the dynamic covalent bonds and thermal mechanical properties for PHU-based vitrimers reported in literature and for those obtained in the present work (updated on July, 2025).

№	PHU-based vitrimer				Reprocessing		Properties				Reference
	Formula	Dynamic bond	Catalyst	E_a (kJ·mol^{-1})	T (°C)	*Time* (min)	T_g (°C) [a]	σ_t (MPa)[b]	ε_t (%)[b]	E (MPa)[b]	
1	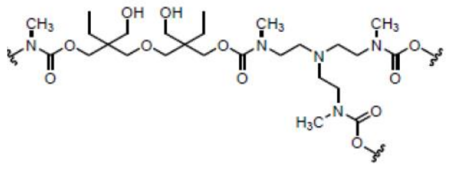	carbamate	internal catalyst	111 ±10	as prepared		54	72.1 ±11.1	7.9 ±3.8	2170 ±370	Ref.[11]
					160	480 (8 h)		53.1 ±8.1	4.8 ±0.8	1640 ±190	
					170	320		43.1 ±3.3	7.3 ±1.0	1380 ±170	
2	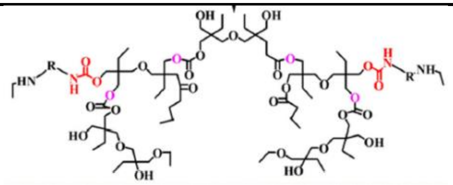	carbonate	-	55	as prepared		-45 – -35	0.8 – 2.1	1342 – 1909	0.01 – 0.03	Ref.[12]
					130	120	-	-	-	-	
3	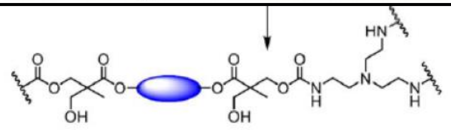	carbamate	-	108 - 136	as prepared		35 – 54	30 ±6 - 82 ±10	2.2 ±0.4 - 198 ±15	600 ±50 - 3000 ±400	Ref.[13]
				132 ±9	as prepared		45	82 ±7	4.5 ±0.4	2300 ±300	
					160	580	-	37 ±17	1.9 ±0.8	2400 ±200	

[11] (b) Fortman, D. J.; Brutman, J. P.; Cramer, C. J.; Hillmyer, M. A.; Dichtel, W. R. Mechanically Activated, Catalyst-Free Polyhydroxyurethane Vitrimers. *J. Am. Chem. Soc.* **2015**, *137* (44), 14019–14022. https://doi.org/10.1021/jacs.5b08084

[12] Wu, H.; Jin, B.; Wang, H.; Wu, W.; Cao, Z.; Wu, J.; Huang, G. A Degradable and Self-Healable Vitrimer Based on Non-Isocyanate Polyurethane. *Front. Chem.* **2020**, *8*. https://doi.org/10.3389/fchem.2020.585569

[13] Fortman, D. J.; Brutman, J. P.; Hillmyer, M. A.; Dichtel, W. R. Structural Effects on the Reprocessability and Stress Relaxation of Crosslinked Polyhydroxyurethanes. *J. Appl. Polym. Sci.* **2017**, *134* (45). https://doi.org/10.1002/APP.44984

4	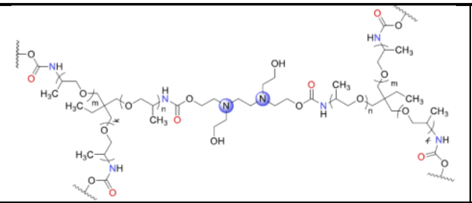	carbamate	internal catalyst	118 ±2	as prepared		-20 – -16	0.40 ±0.05	132 ±21	0.45 ±0.02	Ref.[14]
					130	30	n.d.	0.28 ±0.03	135 ±17	0.41 ±0.07	
5	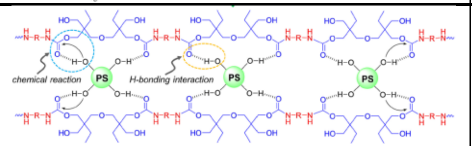 Polysaccharide (chitin, chitosan and sodium alginate)-PHU hybrid vitrimers	carbamate	-	n.d. [d]	as prepared		6.3 – 9.5	1.8 – 10.7	40 - 505	0.9 – 18.6	Ref.[15]
					as prepared		7.9	3.2	406	2.7	
					60	120	n.d.	~3.0	390	n.d.	
6	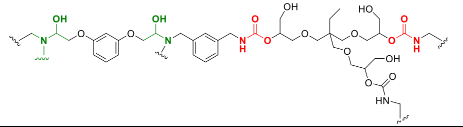	carbamate	-	135.1 ±9.5	as prepared		93.6[c]	102.9 ±6.8	3.9 ±0.3	3061 ±71	Ref.[16]
					180	20	93.2	31.9 ±3.2	1.1 ±0.1	2800 ±100	
7	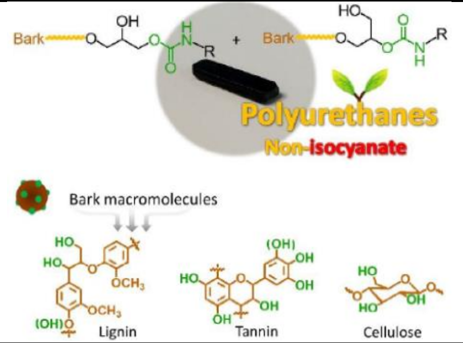	carbamate	-	n.d.	as prepared		20 – 30	19 – 69	15 – 179	n.d.	Ref.[17]
				No recycling							

[14] Hernández, A.; Houck, H. A.; Elizalde, F.; Guerre, M.; Sardon, H.; Du Prez, F. E. Internal Catalysis on the Opposite Side of the Fence in Non-Isocyanate Polyurethane Covalent Adaptable Networks. *Eur. Polym. J.* **2022**, *168*, 111100. https://doi.org/10.1016/j.eurpolymj.2022.111100

[15] Feng, Z.; Zhao, W.; Jin, L.; Zhang, J.; Xue, B.; Ni, Y. Environmentally Friendly Strategy to Access Self-Healable, Reprocessable and Recyclable Chitin, Chitosan, and Sodium Alginate Based Polysaccharide-Vitrimer Hybrid Materials. *Int. J. Biol. Macromol.* **2023**, *240*, 124531. https://doi.org/10.1016/j.ijbiomac.2023.124531

[16] Seychal, G.; Ximenis, M.; Lemaur, V.; Grignard, B.; Lazzaroni, R.; Detrembleur, C.; Sardon, H.; Aranburu, N.; Raquez, J. Synergetic Hybridization Strategy to Enhance the Dynamicity of Poorly Dynamic CO 2 -derived Vitrimers Achieved by a Simple Copolymerization Approach. Adv. Funct. Mater. 2025, 35 (2). https://doi.org/10.1002/adfm.202412268

[17] Chen, H.; Chauhan, P.; Yan, N. "Barking" up the Right Tree: Biorefinery from Waste Stream to Cyclic Carbonate with Immobilization of CO 2 for Non-Isocyanate Polyurethanes. *Green Chem.* **2020**, *22* (20), 6874–6888. https://doi.org/10.1039/D0GC02285C

8	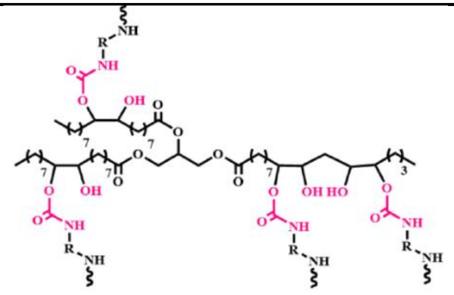	carbamate	-	n.d.	as prepared		20.5 – 30.9	19.3 ±2.6 – 69.2 ±5.8	178.9 ±4.3 – 15.4 ±2.5	137.8 ± 12.5 - 801.9 ± 40.7	Ref.[18]
				No recycling							
9	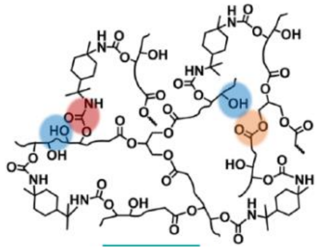	carbamate	-	74.4	as prepared		25	2.2	102	n.d.	Ref.[19]
					140	480	n.d.	1.8	90	n.d.	
10	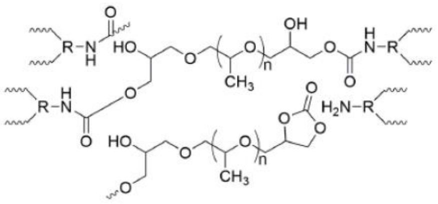	carbamate	-	n.d.	as prepared		-16	1.7 ±0.1	105 ±9	n.d.	Ref.[20]
					140	120	-20	1.6 ±0.1	113 ±13	n.d.	
					as prepared		31	9.7 ±1.1	200 ±18	n.d.	
					140	120	31	9.4 ±1.3	198 ±30	n.d.	

[18] Wang, T.; Deng, H.; Li, N.; Xie, F.; Shi, H.; Wu, M.; Zhang, C. Mechanically Strong Non-Isocyanate Polyurethane Thermosets from Cyclic Carbonate Linseed Oil. *Green Chem.* **2022**. https://doi.org/10.1039/D2GC02910C

[19] Fan, X.; Lin, X.; Shang, H.; Guo, L.; Xu, X.; Liu, H.; Dong, F. Industrial Crops & Products A Cellulose Nanocrystals / Tung Oil-Based Multifunctional Polymer with Good Mechanical Properties, Thermal Stability , Adhesive , Self-Healing , and Recyclable Properties. **2023**, *197* (March). https://doi.org/10.1021/acssuschemeng.1c00033

[20] Chen, X.; Li, L.; Jin, K.; Torkelson, J. M. Reprocessable Polyhydroxyurethane Networks Exhibiting Full Property Recovery and Concurrent Associative and Dissociative Dynamic Chemistry via Transcarbamoylation and Reversible Cyclic Carbonate Aminolysis. *Polym. Chem.* **2017**, *8* (41), 6349–6355. https://doi.org/10.1039/C7PY01160A

11	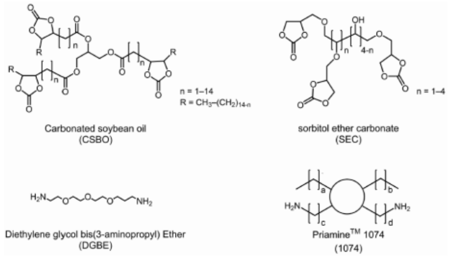	carbamate	-	n.d.	as prepared		-21	0.9 ±0.1	145 ±3	0.9 ±0.0	19[21]
					110	40	-21	0.8 ±0.1	156 ±34	0.8 ±0.1	
					as prepared		-14	0.9 ±0.1	233 ±8	1.1 ±0.3	
					110	40	-14	1.1 ±0.1	209 ±26	1.3 ±0.1	
12	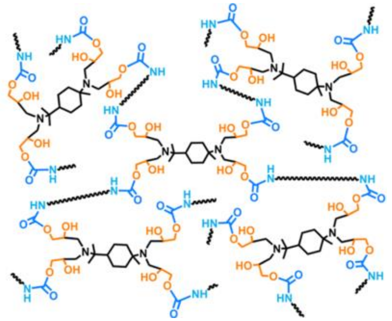	carbamate	-	108.4	as prepared		52.7	35	44	n.d.	Ref.[22]
					160	80	n.d.	26	43	n.d.	
13	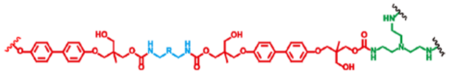	carbamate	-	85.7 - 105.1	as prepared		45 - 74	41 - 65	174 - 452	n.d.	Ref.[23]
				105.1	as prepared		45	47	452	n.d.	
					200	180	37	41	409	n.d.	

[21] Hu, S.; Chen, X.; Torkelson, J. M. Biobased Reprocessable Polyhydroxyurethane Networks: Full Recovery of Crosslink Density with Three Concurrent Dynamic Chemistries. *ACS Sustain. Chem. Eng.* **2019**, *7* (11), 10025–10034. https://doi.org/10.1021/acssuschemeng.9b01239

[22] Hu, S.; Chen, X.; Torkelson, J. M. Biobased Reprocessable Polyhydroxyurethane Networks: Full Recovery of Crosslink Density with Three Concurrent Dynamic Chemistries. *ACS Sustain. Chem. Eng.* **2019**, *7* (11), 10025–10034. https://doi.org/10.1021/acssuschemeng.3c01181

[23] Miao, P.; Jiao, Z.; Liu, J.; He, M.; Song, G.; Wei, Z.; Leng, X.; Li, Y. Mechanically Robust and Chemically Recyclable Polyhydroxyurethanes from CO 2 -Derived Six-Membered Cyclic Carbonates. *ACS Appl. Mater. Interfaces* **2022**, *15* (1), 2246–2255. https://doi.org/10.1021/acsami.2c19251

14	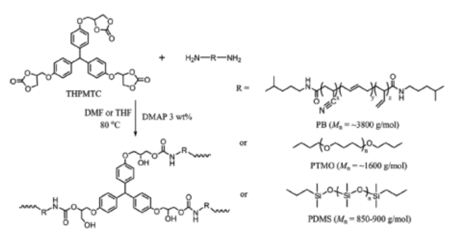	carbamate	-		as prepared		-39	1.5 ±0.2	250 ±45	2.3 ±0.2	Ref.[24]
					140	90	-39	1.7 ±0.1	275 ±30	2.4 ±0.1	
					as prepared		-40	1.1 ±0.1	255 ±15	2.2 ±0.2	
					140	180	-40	1.0 ±0.1	210 ±35	2.7 ±0.3	
15	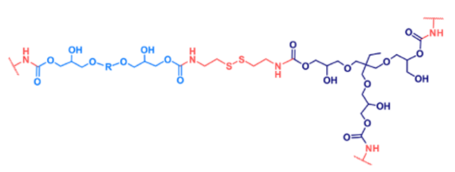	carbamate/disulfide	-	47-62	as prepared		-9 - 44	12.7 ±0.7 – 19.1 ±1.8	1.2 ±0.2 – 2.1 ±0.3	1180 ±80 – 1700 ±310	Ref.[25]
				62	as prepared		40	19.1 ±1.8	2.1 ±0.3	1180 ±80	
					100	20	n.d.	15.3 ±2.2	1.9 ±0.2	1210 ±250	
16	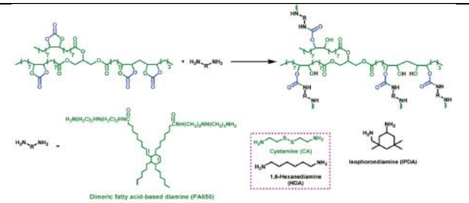	carbamate/disulfide	-	195.9 – 225.8[e]	as prepared		13.6 – 35.7[c]	1.2 - 6.2	152 - 389	n.d.	Ref.[26]
					80	240	n.d.	2.2	270	n.d.	
17	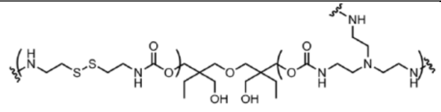	carbamate/disulfide	-	62-126	as prepared		49 – 59	22 ±8 – 43 ±7	2.0 ±0.7 – 4.9 ±1.4	1600 ±100 – 1900 ±300	Ref.[27]
					as prepared		56	34 ±11	2.0 ±0.7	1900 ±100	
					150	30	n.d.	21 ±11	2.1 ±1.1	2200 ±300	

[24] Chen, X.; Li, L.; Wei, T.; Torkelson, J. M. Reprocessable Polymer Networks Designed with Hydroxyurethane Dynamic Cross-links: Effect of Backbone Structure on Network Morphology, Phase Segregation, and Property Recovery. *Macromol. Chem. Phys.* **2019**, *220* (13), 1900083. https://doi.org/10.1002/macp.201900083

[25] Pronoitis, C.; Hakkarainen, M.; Odelius, K. Structurally Diverse and Recyclable Isocyanate-Free Polyurethane Networks from CO2-Derived Cyclic Carbonates. *ACS Sustain. Chem. Eng.* **2022**, *10* (7), 2522–2531. https://doi.org/10.1021/acssuschemeng.1c08530

[26] Dong, J.; Liu, B.; Ding, H.; Shi, J.; Liu, N.; Dai, B.; Kim, I. Bio-Based Healable Non-Isocyanate Polyurethanes Driven by the Cooperation of Disulfide and Hydrogen Bonds. *Polym. Chem.* **2020**, *11* (47), 7524–7532. https://doi.org/10.1039/D0PY01249A

[27] Fortman, D. J.; Snyder, R. L.; Sheppard, D. T.; Dichtel, W. R. Rapidly Reprocessable Cross-Linked Polyhydroxyurethanes Based on Disulfide Exchange. *ACS Macro Lett.* **2018**, *7* (10), 1226–1231. https://doi.org/10.1021/acsmacrolett.8b00667

18	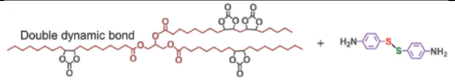	carbamate/disulfide	-	74.2	as prepared		33.5	2.0	160	4.3	Ref.[28]
					100	60	n.d.	1.8	160	n.d.	
19	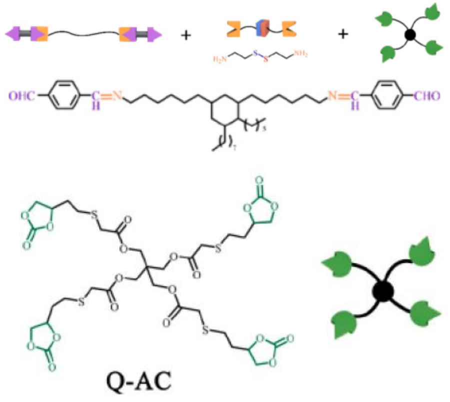	carbamate/disulfide/imine	-	n.d.	as prepared		1.6	2.3 – 4.3	158 - 390	n.d.	Ref.[29]
					140	10	n.d.	3.2 – 4.3	107 - 135	n.d.	
20	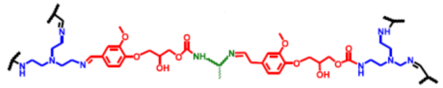	carbamate/ imine	-	81.8–101.8	as prepared		34.5 – 70.4	8 – 79	18 – 296	161 – 642	Ref.[30]
				101.8	as prepared		70.4	79	18	642	
					140	60	46.1	42	10	n.d.	
21	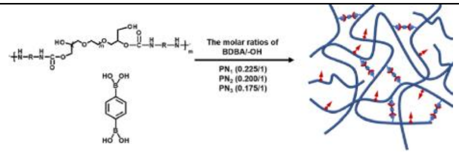	dioxaborolane	-	154.7	as prepared		37.8	16.5	200	n.d.	Ref.[31]
					120	5	n.d.	12.5	240	n.d.	
				147.1	as prepared		49.8	11	350	n.d.	
					120	5	n.d.	7	500	n.d.	

[28] Yang, X.; Wang, S.; Liu, X.; Huang, Z.; Huang, X.; Xu, X.; Liu, H.; Wang, D.; Shang, S. Preparation of Non-Isocyanate Polyurethanes from Epoxy Soybean Oil: Dual Dynamic Networks to Realize Self-Healing and Reprocessing under Mild Conditions. *Green Chem.* **2021**, *23* (17), 6349–6355. https://doi.org/10.1039/D1GC01936H

[29] Mou, X.; Yang, Z.; Lai, X.; Ding, J.; Chen, Y.; Li, H.; Zeng, X. Self-Healing and Reprocessable Biobased Non-Isocyanate Polyurethane Elastomer with Dual Dynamic Covalent Adaptive Network for Flexible Strain Sensor. *Chem. Eng. J.* **2024**, *493*, 152876. https://doi.org/10.1016/j.cej.2024.152876

[30] Fan, M.; Wang, Y.; Zhao, J. High-Strength, Reprocessable, and Degradable Bio-Based Non-Isocyanate Polyurethanes with Dual Dynamic Covalent Bonds. *J. Clean. Prod.* **2025**, *513*, 145732. https://doi.org/10.1016/j.jclepro.2025.145732

[31] Ding, Y.; Xu, W.; Kuang, T.; Wen, Q.; Cheng, C.; Chen, F. Re-Produced and Moisture-Sensitive Covalent Adaptable Networks Based on Non-Isocyanate Polyurethanes. *Polymer (Guildf).* **2023**, *272*, 125845. https://doi.org/10.1016/j.polymer.2023.125845

22	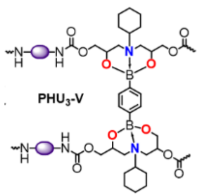	dioxazaborocane	-	141 ±4.3	as prepared		109<br>(by DSC)	55.3 ±2.3.1	5.6 ±0.6	1170 ±35	**This work**
					170	10	n.d.	63.2 ±8.6	5.8 ±0.7	1365 ±125	

[a] Glass transition temperature by DSC.
[b] Tensile strength (σ_t), elongation at break (ε_t) and Young's modulus (E) at RT.
[c] Determined by DMTA.
[d] Not determined.
[e] Determined by DMTA
